# Epigenetic Variance, Performing Cooperative Structure with Genetics, Is Associated with Leaf Shape Traits in Widely Distributed Populations of Ornamental Tree *Prunus mume*

**DOI:** 10.3389/fpls.2018.00041

**Published:** 2018-01-30

**Authors:** Kaifeng Ma, Lidan Sun, Tangren Cheng, Huitang Pan, Jia Wang, Qixiang Zhang

**Affiliations:** ^1^Beijing Key Laboratory of Ornamental Plants Germplasm Innovation and Molecular Breeding, National Engineering Research Center for Floriculture, Beijing Laboratory of Urban and Rural Ecological Environment, Key Laboratory of Genetics and Breeding in Forest Trees and Ornamental Plants of Ministry of Education, School of Landscape Architecture, Beijing Forestry University, Beijing, China; ^2^Beijing Advanced Innovation Center for Tree Breeding by Molecular Design, Beijing Forestry University, Beijing, China

**Keywords:** DNA methylation, molecular marker, genetic/epigenetic diversity, genetic/epigenetic structure, environmental factors, association analysis, *Prunus mume*

## Abstract

Increasing evidence shows that epigenetics plays an important role in phenotypic variance. However, little is known about epigenetic variation in the important ornamental tree *Prunus mume*. We used amplified fragment length polymorphism (AFLP) and methylation-sensitive amplified polymorphism (MSAP) techniques, and association analysis and sequencing to investigate epigenetic variation and its relationships with genetic variance, environment factors, and traits. By performing leaf sampling, the relative total methylation level (29.80%) was detected in 96 accessions of *P*. *mume*. And the relative hemi-methylation level (15.77%) was higher than the relative full methylation level (14.03%). The epigenetic diversity (*I*^∗^ = 0.575, *h*^∗^ = 0.393) was higher than the genetic diversity (*I* = 0.484, *h* = 0.319). The cultivated population displayed greater epigenetic diversity than the wild populations in both southwest and southeast China. We found that epigenetic variance and genetic variance, and environmental factors performed cooperative structures, respectively. In particular, leaf length, width and area were positively correlated with relative full methylation level and total methylation level, indicating that the DNA methylation level played a role in trait variation. In total, 203 AFLP and 423 MSAP associated markers were detected and 68 of them were sequenced. Homologous analysis and functional prediction suggested that the candidate marker-linked genes were essential for leaf morphology development and metabolism, implying that these markers play critical roles in the establishment of leaf length, width, area, and ratio of length to width.

## Introduction

*Prunus mume* Sieb. et Zucc. (2*n* = 2*x* = 16), also known as mei, was domesticated in China more than 3,000 years ago as an ornamental plant and fruit tree (Zhang et al., 2012). Its distribution is centered around the borders of northwestern Yunnan Province, southwestern Sichuan Province, and southeastern Tibet Autonomous Region (Bao, 1993; Bao and Chen, 1994). The wild form of *P*. *mume* can also be found across a wide region south of Changjiang River (Zhang et al., 2010). Genetic diversity, population structure, and genetic linkage mapping analyses on *P. mume* have investigated the molecular markers that support the hypothesis that the genetic diversity center of the species is southwest China (Yang et al., 2007). A high-density linkage map of *P*. *mume* was constructed and used to detect quantitative trait loci (QTLs) (Sun et al., 2014), and specific-locus amplified fragment sequencing (SLAF-seq) marker located on linkage group 7 was associated with branch weeping (Zhang J. et al., 2015; Zhang et al., 2017). Molecular identification also proved that fruiting mei and Japanese flowering mei originated in different locations (Shen et al., 2011). Importantly, the complete genome sequence of *P*. *mume* is now available (Zhang et al., 2012), and this has provided new sights into the genetics of this species. However, little is known about the variation or regulation mechanisms involved in epigenetics in *P*. *mume*.

In eukaryotes, genetic variation and epigenetic variation both play important roles in determining phenotypic characteristics by regulating gene expression (Massicotte et al., 2011; Mirouze and Paszkowski, 2011; Heyn et al., 2013). Increasing evidence has revealed that epigenetic modifications, such as patterns of DNA methylation (Dubrovina and Kiselev, 2016), histone modifications (Thorstensen et al., 2011), histone variants (Chen et al., 2011), and small RNAs (Heo et al., 2013), can be passed from one generation to the next via mitosis or meiosis, or can change either spontaneously or in response to external signals (Pérez et al., 2006; Fang and Chao, 2007; Becker and Weigel, 2012; Geoghegan and Spencer, 2012; Li et al., 2017). And the variations in the epigenome are of utmost importance during development and in response to changing environmental conditions (Feng et al., 2010; Feng and Jacobsen, 2011; Schmitz and Ecker, 2012; Nicotra et al., 2015).

DNA methylcytosine, which suppresses transposable elements (Lisch and Bennetzen, 2011), changes flower symmetry (Cubas et al., 1999), and influences fruit ripening (Manning et al., 2006; Seymour et al., 2008), is a widely studied and common epigenetic feature in plant genomes (Bennetzen and Zhu, 2011). Methylcytosine, which comprises 6–30% of the total cytosine in a genome (Chen and Li, 2004), generally occurs in the symmetrical sequence CG, but can also be found in CHG or CHH (H = A, T, or C) sequences (Gruenbaum et al., 1981; Lister et al., 2008). Methylcytosine also gives rise to epialleles that are potentially reversible and often exist in metastable states (Vaughn et al., 2007; Foerster et al., 2011), which provides the opportunity for uncovering epigenetic diversity and structure within or between populations (Liu et al., 2012; Ma et al., 2013; Foust et al., 2016) and epigenetic linkage mapping (Zhang et al., 2008; Long et al., 2011), and as well as increases the efficiency of breeding programs and achieving crop improvement (Manning et al., 2006; Seymour et al., 2008). However, compared with the application of population genetics that has revealed how genetic diversity, structure and linkage/association analysis can be performed (Fisher et al., 2013; Ye et al., 2015; Du et al., 2016), the significance of variation in epigenetic states at the population level presents a complex challenge and remains largely unexplored (Richards, 2008; Cao et al., 2011). This difference exists because epialleles as a cause of phenotypic variance are more difficult to identify than genetic variation associated with DNA mutations (Chodavarapu et al., 2012).

Herein, in order to investigate epigenetic indices of diversity and structure, as well as the relationships between epigenetic variants and genetic variations, environmental factors, and traits, we used the amplified fragment length polymorphism (AFLP) and methylation-sensitive amplified polymorphism (MSAP) techniques together with multivariate statistics and association analysis, as well as short fragment sequencing to search for markers in the *P. mume* genome. We also explored the function of candidate marker-linked genes based of the sequences of candidate markers. Our results will help breeders to understand and use epigenetic variation to accelerate the improvement of *P*. *mume* plants.

## Materials and Methods

### Plant Material and DNA Isolation

**FIGURE 1:**
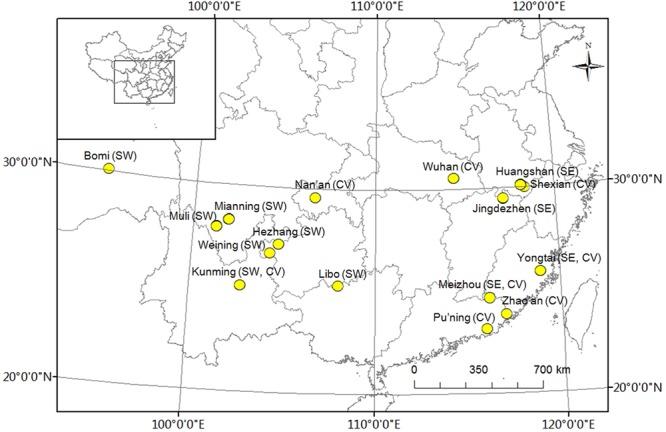
Distribution of experimental *Prunus mume* samples. ‘SW’ represents samples collected in southwest China, ‘SE’ represents samples collected in southeast China, and ‘CV’ represents cultivated varieties.

Three groups of two people each collected and measured leaf samples from 1a branches of 96 accessions of *P*. *mume*. The leaves were snap-frozen in liquid nitrogen for DNA extraction and genotyping. Thirty leaves of each genotype from the three populations were harvested with three replications and this collection was performed in the main areas where wild plants of *P*. *mume* are distributed, namely, southwest China and southeast China, as well as sites where they are cultivated (**Figure 1**), on 11–21 September 2013. Leaf shape traits, including length, width, area, and ratio of length to width, were measured using a portable laser leaf area meter (CI-202, CID Bio-Science, Inc., United States) and tested using one-way analysis of variance (ANOVA). The geographic coordinates and altitude were recorded using a handheld GPS (UniStrong, Co., Ltd., China) (Supplementary Table 1). Plant materials were ground in liquid nitrogen and total DNA was isolated using the CTAB method, detected using a NanoVue UV/visible spectrophotometer (GE Healthcare Limited, Sweden), and stored at -80°C (Ma et al., 2013).

### Meteorological Data

Meteorological data, including average, lowest, and highest daily temperatures, average and minimum daily relative humidity, total daily precipitation, and total daily sunshine time, between 01 September 2012, and 31 August 2013, were acquired from a Chinese meteorological data sharing service^1^. Parameters of annual and monthly average temperatures, average daily lowest and average daily highest temperatures, average relative humidity, average minimum relative humidity, total precipitation, and total sunshine time, were then calculated (Supplementary Table 1).

### Detection of Genetic Markers

To detect genetic markers, an AFLP technique including digestion with the combination of restriction endonucleases *Eco*RI and *Mse*I, ligation, and pre- and selective-amplification was adopted, in accordance with the work of Vos et al. (1995). The 15 fluorescently labeled primer pairs used for *Eco*RI/*Mse*I enzyme combination AFLP selective-amplifications were shown in Supplementary Table 2. Instead of silver staining, a fluorescent capillary electrophoresis detection method (Beijing Microread Gene Technology, Co., Ltd., Beijing, China) that is more sensitive, faster, and safer was used to resolve the selective amplification products. Marker bands were revealed by GeneMarker V1.7.1 and transformed into a binary character matrix with “0” for absence and “1” for presence (Ma et al., 2013).

### Detection of Genomic Methylation Markers

The MSAP marker detection procedure was adapted from Ma et al. (2013) and was similar to the AFLP technique described above, except for the double digestion with the restriction endonuclease combinations *Eco*RI/*Hpa*II and *Eco*RI/*Msp*I and the use of corresponding adapters and fluorescently labeled primers instead of *Eco*RI/*Mse*I. The 15 fluorescently labeled primer pairs used for the MSAP selective amplifications with the *Eco*RI/*Hpa*II and *Eco*RI/*Msp*I enzyme combinations were shown in Supplementary Table 3. Epigenetic bands generated from GeneMarker V1.7.1 were transformed into a binary character matrix with “0” for absence and “1” for presence.

### Statistical Analysis of Diversity, Methylcytosine Level, and Structure

Environmental factors included geographic coordinates and meteorological information, and the annual (01 September 2012 to 31 August 2013) and monthly (01 August 2013 to 31 August 2013) values of which were estimated using the daily data downloaded from the database of a Chinese meteorological data sharing service (Supplementary Table 1). These data were transformed in ADE-4 software as:

xij↦(xij−mj)/sjwith mj=∑ipixijandsj=∑ipi(xij−mj)2

where *x_ij_* represents the value of the *i*th and *j*th columns and *p_i_* represents the *i*th row weight (Thioulouse et al., 1996).

The AFLP markers were used to investigate genetic diversity and structure. These genetic markers were analyzed using STRUCTURE v.2.3.4 software to detect uniformity within each population based on Bayesian clustering (Evanno et al., 2005). Genetic diversity (*I*, Shannon’s information index; *h*, genetic diversity; *uh*, unbiased genetic diversity) was estimated using GenAlEx 6.5 (Peakall and Smouse, 2012), the genetic differentiation coefficient was calculated as *G_ST_* = (*h_total_* -*h_pop_*)/*h_total_* and the gene flow was estimated as *Nm* = (1 -*G_ST_*)/4 *G_ST_* (McDermott and McDonald, 1993; Bussell, 1999).

The among-population variances were also structured and maximized using a between-group eigenanalysis, namely, between-group principal component analysis (BPCA)-PCA among groups based on the PCA among individuals (Parisod and Christin, 2008). Based on Euclidean distances, this method can divide the genetic variance into within- and between-population components, enabling *β_ST_*, equal to the ratio of the inertia between populations to the total inertia and analogous to the *F*-statistic, to be obtained (Parisod et al., 2005). The significance of differences among populations was determined by a Romesburg randomization test (9999 permutations) in ADE-4 software (Thioulouse et al., 1996).

The MSAP markers were used to calculate epigenetic variance and the “0, 1” matrix was transformed and redefined. Four patterns of methylation were defined according to the emerging bands: (i) present in both *Eco*RI/*Hpa*II and *Eco*RI/*Msp*I (1,1), non-methylation; (ii) absent in *Eco*RI/*Hpa*II but present in *Eco*RI/*Msp*I (0,1), full methylation; (iii) present in *Eco*RI/*Hpa*II but absent in *Eco*RI/*Msp*I (1,0), hemi-methylation; and (iv) absent in both *Eco*RI/*Hpa*II and *Eco*RI/*Msp*I (0,0), uninformative methylation. We defined an additional pattern of methylation as, (v) total methylation, the summation of full methylation and hemi-methylation (Ma et al., 2012; Ci et al., 2015).

The methylation/non-methylation levels were defined as the ratio of the number of bands with one pattern to the total number of bands in one genotype or population. These methylation/non-methylations are relative because only 5′-CCGG sites, not all methylcytosine residues, can be detected using MASP. Within each population, relative full methylation and hemi-methylation levels, and relative total methylation and non-methylation levels were compared by Wilcoxon’s rank-sum test. Among populations, the relative total methylation and non-methylation levels, and relative full methylation and hemi-methylation levels were both examined using a Kruskal–Wallis *H* test (Lira-Medeiros et al., 2010).

Scoring epiloci with (i) non-methylation and (iv) uninformative methylation as “0,” and epiloci with (ii) full methylation and (iii) hemi-methylation as “1,” we reconstructed a methylation-sensitive polymorphism (MSP) profile and calculated the epigenetic diversity (*I*^∗^, Shannon’s information index; *h*^∗^, epigenetic diversity; *uh*^∗^, unbiased epigenetic diversity) by using GenAlEx 6.5 (Foust et al., 2016). Significant differences in epigenetic diversity among populations were detected by the Kruskal–Wallis *H* test. The epigenetic differentiation coefficient was calculated as *G_ST_*^∗^ = (*h_total_*^∗^ -*h_pop_*^∗^)/*h_total_*^∗^ (McDermott and McDonald, 1993; Bussell, 1999).

Based on the MSP profile, we determined the epigenetic variance and structure among populations using a between-group eigenanalysis, similar to the method described above. The contributions of epigenetic and genetic profiles to variance of the structured population were evaluated using a symmetrical co-inertia analysis. The test of significant differences in epigenetic and genetic structures was performed by 9999 Monte Carlo permutations in ADE-4 software (Thioulouse et al., 1996). Similarly, a cooperative structure with matching information between the epigenetic matrix and environmental factors was also established according to the processes of ADE-4.

### Association Analysis and Sequencing of the Candidate Markers

To investigate the correlation between relative methylation level and phenotype, we performed a linear correlation analysis (Ma et al., 2012), as well as an association analysis using an MLM model within the software package TASSEL 2.1 (Bradbury et al., 2007). Parameters of membership probability (Q-matrix) and pairwise kinship (K-matrix), which were used to evaluate the effects of population genetic structure and relatedness between each pair of individuals, were estimated based on 1,864 AFLP markers following the operating processes of the STRUCTURE v.2.3.4 and TASSEL 2.1 software packages, respectively. Epimarker association analysis was performed similarly to genetic marker association using the same Q-matrix and K-matrix, but with the four epigenotype patterns: (i) non-methylation, (ii) full methylation, (iii) hemi-methylation, and (iv) uninformative site.

The products generated from the AFLP and MSAP selective amplifications were monitored using polyacrylamide gel electrophoresis (PAGE) and silver staining techniques as described by Bassam et al. (1991), respectively. Candidate markers were recycled based on the PAGE results, and reamplified, transformed, and cloned (Ma et al., 2012). Positive clones were detected using the selective-amplification protocol described above, and sequenced at Ruibiotech Co., Ltd. (Beijing, China). Sequences homology analysis and function prediction were performed using the NCBI^2^ and JGI^3^ databases.

## Results

### Genetic Diversity and Structure

We used an AFLP technique with 15 selective primer-pair combinations to detect polymorphic sites in the *P. mume* genome. A total of 1,864 polymorphic markers were detected out of 2,254 total bands (Supplementary Table 2). The genetic diversity of each of the 96 accessions was calculated and showed a high level of genetic diversity, as evaluated globally by Shannon’s diversity index (*I* = 0.484), and Nei’s gene diversity index (*h* = 0.319). However, the gene diversity index (*h*) among the three populations, measured as 0.305 (cultivated population), 0.291 (wild population in southwest China), and 0.293 (wild population in southeast China), did not differ significantly (*P* = 0.105). This might be caused by a downward bias produced by related or inbred individuals within the accessions (Degiorgio et al., 2010). Therefore, another parameter, the gene unbiased diversity (*uh* = 0.322) that showed a significant difference (*P* = 0.006) among the populations, was introduced. A low genetic differentiation coefficient (*G_ST_* = 0.044) and high gene flow (*Nm* = 5.432) of the cultivated population indicated there was strong gene exchange among the cultivated individuals, possibly a result of selective breeding from objective traits. In contrast, the high *G_ST_* and low *Nm* of wild types indicated a high level of population differentiation and low gene exchange, respectively (**Table 1**).

**Table 1:** The genetic diversity, differentiation coefficient and gene flow across the three populations^a^.

Populations	Number	*Na*	*Ne*	*I*	*h*	*uh*	*G_ST_*	*Nm*
Cultivated population	59	1.967	1.506	0.466	0.305	0.310	0.044	5.432
Wild population in southwest China	23	1.852	1.485	0.442	0.291	0.304	0.089	2.559
Wild population in southeast China	14	1.776	1.490	0.444	0.293	0.316	0.081	2.836
Mean	32	1.865	1.494	0.451	0.296	0.310	—	—
Total	96	2	1.535	0.484	0.319	0.322	0.071	3.271

*^*a*^The significance of differences among populations was analyzed by Kruskal–Wallis *H* test (*I*, chi-square = 4.230, *df* = 2, *P* = 0.121; *h*, chi-square = 4.505, *df* = 2, *P* = 0.105; *uh*, chi-square = 10.299, *df* = 2, *P* = 0.006).*

**FIGURE 2:**
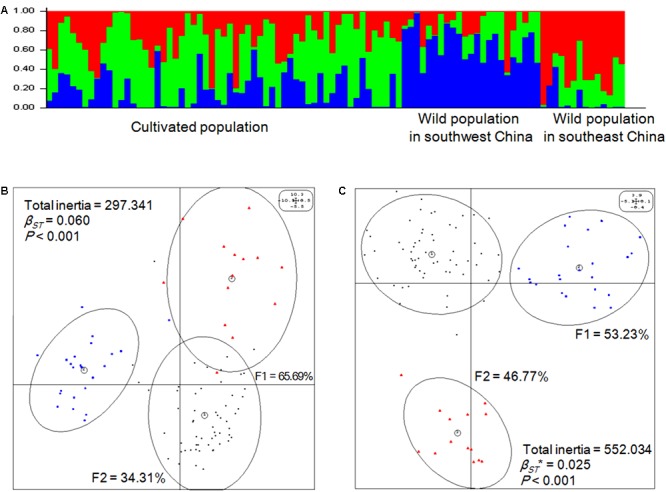
Genetic and epigenetic structures of *Prunus mume*. **(A)** Genetic populations were detected using STRUCTURE (v.2.3.4) software [*K* = 3, Ln Pr(X| K) = -83680.1] based on Bayesian clustering. **(B)** Eigenanalysis among the three populations of *P*. *mume* using principal component analysis (PCA) values generated from genetic covariance matrices based on the AFLP profile. F1 and F2 values show the contributions of the first two principal components summarizing the total variance of each data set. *β_ST_* was calculated by between-group PCA (BPCA) for genetic profiles and tested with Romesburg randomization permutations. Numbers within circles represent the populations: (1) cultivated population, each individual genotype is represented by a small dark circle; (2) wild population in southwest China, each individual genotype is represented by a blue quadrate; (3) wild population in southeast China, each individual genotype is represented by a red triangle. **(C)** Eigen analysis among the three populations of *P*. *mume* using PCA values generated from epigenetic covariance matrices based on the methylation-sensitive polymorphism (MSP) profile. *β_ST_*^∗^ was calculated by BPCA for epigenetic profiles and tested with Romesburg randomization permutations. Numbers within circles represent the populations: (1) cultivated population, each individual epigenotype is represented by a small dark circle; (2) wild population in southwest China, each individual epigenotype is represented by a blue quadrate; (3) wild population in southeast China, each individual epigenotype is represented by a red triangle.

Genetic structure was analyzed first using a Bayesian clustering test (*K* = 3, Ln Pr(X|K) = -83680.1), which showed that the 96 accessions could be divided into three populations that were significantly separated from each other (**Figure 2A**). Then, the same molecular profile was used for eigenanalysis (BPCA-PCA among groups based on the PCA among individuals), which, as expected, produced the same division (**Figure 2B**). According to eigenanalysis, the molecular genetic variation based on each individual, and visualized as a two-dimensional plot produced by PCA using the first two principal components (F1 = 65.69%, F2 = 34.31%), could be divided into two parts, namely, between (inertia = 17.95) and within (inertia = 279.39) populations (*P* < 0.001), similar to *F*-statistics. These results verified the reliability of the classification of the 96 accessions into three populations.

### Relative Genomic Methylation Levels

The MSAP approach identified methylation sites by double digestion of total DNA with *Eco*RI/*Hpa*II or *Eco*RI/*Msp*I. A total of 2,849 markers which ranged in length from 59 to 712 bp and had 2,810 polymorphic sites were identified (Supplementary Table 3). Only 5′-CCGG sites, not all methylcytosines, were identified in the whole genome. Therefore, the methylation and non-methylation levels in the genome of *P. mume* are relative and were classified as relative full methylation (14.03%), relative hemi-methylation (15.77%), relative total methylation (29.80%), relative non-methylation (31.83%), and uninformative sites (38.37%). The results showed that relative hemi-methylation was significant higher than relative full methylation (*P* = 0.005), but the difference between the relative total methylation level and relative non-methylation level was not significant (**Table 2**). These results suggested that different patterns of DNA methylation levels may play important roles within the *P. mume* genome.

**Table 2:** Relative genomic methylation/non-methylation levels in the three populations of *Prunus mume*.

	Mean	*SD*	CV (%)	Min	Max	Significant difference among populations^a^		
						χ^2^	*df*	*P*-value
Hemi-methylation^b^	15.77	3.41	21.62	7.72	27.69	4.586	2	0.101
Full methylation^b^	14.03	3.56	25.35	7.69	24.29	3.466	2	0.177
Total methylation^b^	29.80	4.33	14.53	17.97	40.75	7.732	2	0.021
Non-methylation^b^	31.83	7.11	22.35	18.85	59.50	1.189	2	0.552
Uninformative locus	38.37	6.11	15.93	18.74	49.84	5.240	2	0.073

*^*a*^The significance of differences among the three populations was examined by Kruskal–Wallis *H* test. ^*b*^Significant difference between relative hemi-methylation and full methylation levels was examined by Wilcoxon’s rank-sum test (*Z* = -2.817, *df* = 1, *P* = 0.005). Significant difference between relative total methylation and non-methylation levels was examined by Wilcoxon’s rank-sum test (*Z* = -1.678, *df* = 1, *P* = 0.093).*

A significant difference in relative total methylation among the three populations was detected by Kruskal–Wallis *H* test (*P* = 0.021) (**Table 2** and Supplementary Figure 1). In addition, the relative total methylation level of the cultivated plants was higher than the relative total methylation levels of the wild plants in the southwest and southeast China populations (Supplementary Figure 1). In each population, no significant difference was detected between relative hemi-methylation and full methylation levels, or no significant difference was found between relative total methylation and non-methylation levels indicating the environmental plasticity of DNA methylation levels (Supplementary Figure 1).

### Epigenetic Diversity and Its Comparison with Genetic Diversity

Based on the MSAP markers of each individual, the three parameters, Shannon’s diversity index (*I*^∗^), epigenetic diversity index (*h*^∗^) and epi-gene unbiased diversity (*uh*^∗^), were assessed and showed that the epigenetic diversity was significant difference among the three populations (*P* < 0.001). Compared with the results obtained using the genetic information, all three parameters were higher (*I*^∗^ > *I, h*^∗^ > *h, uh*^∗^ > *uh*) although they showed similar tendencies (**Tables 1, 3**). Together with the parameters *I* and *h* displayed no significant differences among the three populations, the results indicated that the epigenetic diversity (*I*^∗^ and *h*^∗^) of *P*. *mume* was high with a low epigenetic differentiation coefficient (*G_ST_*^∗^ = 0.033) and was easily affected by the environment (or cultivation). Further, the epigenetic diversity index of the wild population in southwest China was lower than the epigenetic diversity index of the wild population in southeast China. A high epigenetic differentiation coefficient (0.064) was obtained for the wild population in southwest China (**Table 3**), and this phenomenon occurred within epigenetic markers, indicating that high epigenetic variation was present in the population of southwest China.

**Table 3:** Epigenetic diversity and differentiation coefficient among the three populations^a,b^.

Populations	Number	*N*a^∗^	*Ne*^∗^	*I*^∗^	*h*^∗^	*uh*^∗^	*G_ST_*^∗^
Cultivated population	59	1.992	1.683	0.570	0.389	0.396	0.004
Wild population in southwest China	23	1.959	1.634	0.545	0.368	0.385	0.064
Wild population in southeast China	14	1.925	1.680	0.560	0.383	0.413	0.025
Mean	32	1.958	1.666	0.558	0.380	0.398	—
Total	96	2	1.690	0.575	0.393	0.397	0.033

*^*a*^Among-population significance test of parameters, *I*^∗^ (chi-square = 65.890, *df* = 2, *P* < 0.001), *h*^∗^ (chi-square = 69.279, *df* = 2, *P* < 0.001), and *uh*^∗^ (chi-square = 217.872, *df* = 2, *P* < 0.001), was carried out by Kruskal–Wallis *H* test. ^*b*^Comparison of diversity parameters between genetic and epigenetic levels was carried out by Kruskal–Wallis *H* test (*I*^∗^, chi-square = 254.628, *df* = 1, *P* < 0.001; *h*^∗^, chi-square = 254.628, *df* = 1, *P* < 0.001; *uh*^∗^, chi-square = 254.628, *df* = 1, *P* < 0.001).*

### Epigenetic Structure and Its Relationships with Genetic Structure, and Environment

Based on the MSP profile, a between-group analysis was carried out and the epigenetic variance was visualized in a two-dimensional plot based on the first two principal components explaining 100% of the variance, which was separated into between- (inertia = 13.79) and within-group components (inertia = 538.24). The analysis revealed that the 96 accessions of *P. mume* significantly separated into three populations based on epigenetic markers (*P* < 0.001) (**Figure 2C**), which was similar to the result based on genetic markers. Thus, the population division based on epigenetic markers was consistent with population division based on genetic markers.

**FIGURE 3:**
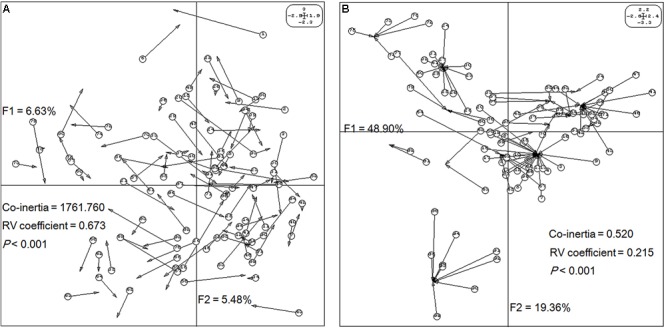
Structure matching analysis of *Prunus mume* based on genetic variables, epigenetic variables, and environmental variables. F1 and F2 values show the contributions of the first two principal components summarizing the total variance of each data set. **(A)** Co-inertia analysis of *P*. *mume* using principal component analysis (PCA) scores based on a methylation-sensitive polymorphism (MSP) covariance matrix (epigenetic variables) and AFLP covariance matrix (genetic variables). Each arrow positions an individual by its epigenetic variables (arrow start and number in a circle) and its genetic variables (arrow end). **(B)** Co-inertia analysis of *P*. *mume* using PCA scores based on a MSP covariance matrix (epigenetic variables) and environmental covariance matrix (environmental variables). Each arrow positions an individual by its epigenetic variables (arrow start and number in a circle) and its environmental variables (arrow end).

Next, the relationships between epigenetic structure and genetic structure, and environmental factors were revealed by co-inertia analysis, respectively. The markers and environment factors were transformed into multi-dimensional spatial based on Euclidean distance (Thioulouse et al., 1996), to reveal the distribution of MSAP marker within the spatial constitution of AFLP, and environmental factors. The space matching information of epigenetic and genetic markers was visualized using the first two principal components explaining 12.11% of the co-inertia, which revealed notable cooperativity (*P* < 0.001) in their structures (**Figure 3A**). The relationship between epigenetic structure and environmental factors also exhibited cooperativity (*P* < 0.001), and the first two principal components in the profiles explained 48.90 and 19.36% of the co-inertia, respectively (**Figure 3B**). These results indicated that the epigenetic structure shared common units and performed cooperativity with both the genetic structure, and environmental structure.

### Leaf Shape Traits and Linear Correlation, Association Analyses

**FIGURE 4:**
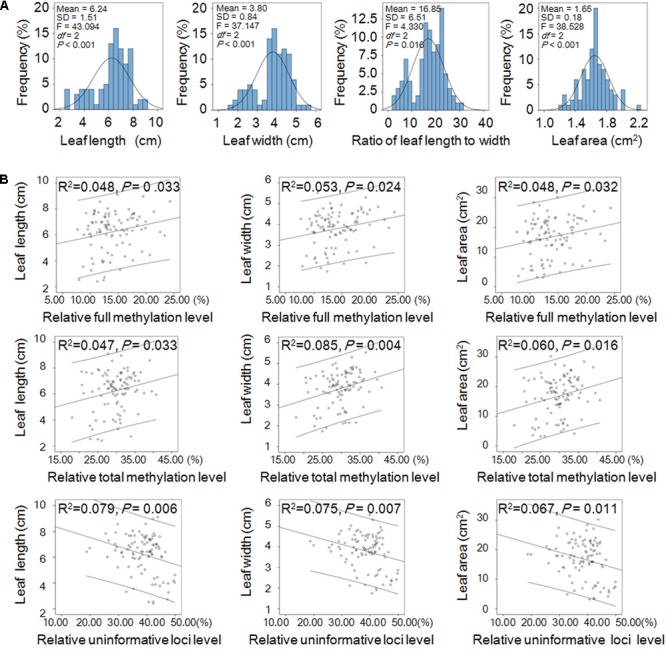
Leaf shape traits and their linear correlations with relative DNA methylation levels in *Prunus mume*. **(A)** Frequency distribution of the shape traits including leaf length, width, area, and ratio of length to width of *P*. *mume* and one-way ANOVA (*P* < 0.05) among the three populations. **(B)** Linear correlations between leaf shape traits and relative DNA methylation levels. Abscissa and ordinate represent relative methylation/uninformative loci levels, and leaf phenotype, respectively. The 95% prediction limits are shown within the hyperbolas in each graph.

The leaf shape traits (including leaf length, 6.24 ± 1.51 cm; width, 3.80 ± 0.84 cm; area, 16.85 ± 6.51 cm^2^; and ratio of length to width, 1.65 ± 0.18) displayed normal distribution and significant differences among the three populations (**Figure 4A**). Linear correlation analysis (two-tailed) showed that the relative full methylation level and relative total methylation level were positively correlated with leaf length, width, and area, but the relative uninformative loci were negatively correlated with leaf length, width, and area (**Figure 4B**). These results suggested that DNA methylation levels were correlated with positive regulation of leaf phenotype.

**FIGURE 5:**
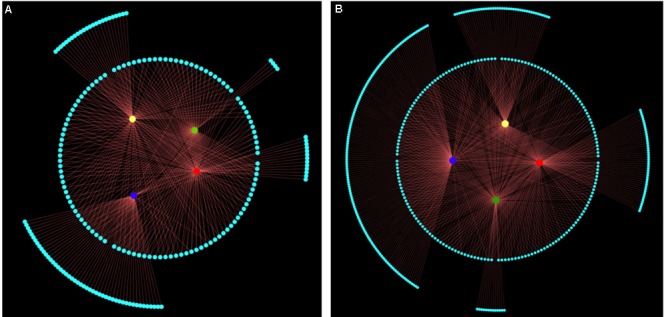
All significant associated markers represented by structural networks (*P* < 0.05). Nodes in the innermost circle represent leaf length (red), width (yellow), area (green), and ratio of leaf length to width (blue). Nodes with gray color in the middle circle represent multi-function associated markers. Nodes with gray color in the outermost circle represent trait-specific associated markers. Edge size represents ratio of variant explained by associated markers, which explained 1.69-15.42% of leaf shape traits variance. **(A)** The 203 genetic markers associated with leaf length (total/trait-specific associated markers, 109/13), width (105/24), area (105/4), and ratio of leaf length to width (62/49). **(B)** The 495 epigenetic markers associated with leaf length (188/46), width (164/43), area (168/13), and ratio of leaf length to width (175/140).

Next, we performed an association analysis to examine the relationship between molecular markers and leaf shape traits based on an MLM model within the Tassel 2.1 software package (Bradbury et al., 2007). In total, 113 multi-function and 90 trait-specific associated genetic markers were detected. Among these 203 AFLP markers, 109, 105, 62, and 105 (explaining 1.69–8.73% of variation) were significantly associated with leaf length, width, area, and ratio of length to width, respectively (**Figure 5A** and Supplementary Table 4). As shown in **Figure 5B**, 423 MASP markers, including 181 multi-function and 242 trait-specific markers, were associated with phenotype. Among them, 188, 164, 168 and 175 MSAP markers (explaining 1.76–15.42% of variance) were associated with leaf length, width, area, and ratio of leaf length to width, respectively. Among these markers, 46, 43, 13, and 140 were trait-specific and associated with leaf length, width, area, and ratio of leaf length to width, respectively (**Figure 5B** and Supplementary Table 5). These associated genetic/epigenetic markers provide important molecular evidence that can be used to reveal the gene regulation of complex quantitative traits of leaf.

### Function Prediction of the Linkage Genes

The candidate markers were sequenced following the PAGE and silver staining processes (Supplementary Figure 2) to predict the functions of the linkage genes. A total of 38 AFLP and 30 MSAP markers associated with leaf traits (explaining 1.70–8.73% and 3.45–6.61% of the traits variation, respectively) were sequenced successfully (Supplementary Data 1). Homology analysis (Supplementary Table 6) showed that one candidate maker-linked gene was origin from chloroplast [*PmcpGENE-1* (MSAP-2258)] and two were from mitochondria [*PmMatR* (AFLP-1128) and *PmmtGENE-1* (MSAP-2046)]. Five maker-linked genes were transcription factor genes [*PmTCP1* (AFLP-1113), *PmMatR* (AFLP-1128), *PmmTERF5* (AFLP-1475), *PmGATA5-LIKE* (MSAP-2025), and *PmTIFY4B-LIKE* (AFLP-2491)]; four maker-linked genes encoded chloroplast proteins [*PmCGS* (AFLP-950), *PmmTERF5* (AFLP-1475), *PmQS* (MSAP-1679), *PmPPR* (MSAP-1954)]; and three makers linked genes encoded mitochondria proteins [*PmLBD1-LIKE* (AFLP-305), *PmSHM2* (AFLP-512), *PmMatR* (AFLP-1128)] (Supplementary Table 6).

Importantly, as displayed in Supplementary Table 6, these marker-linked genes were enriched in the following GO terms: leaf development [*PmLBD1-LIKE*(AFLP-305)], glycine hydroxymethyltransferase activity [*PmSHM2* (AFLP-512)], RNA splicing [*PmMatR*(AFLP-1128)], signal transduction [*PmCaMBPs* (AFLP-543)], transporter activity [*PmNPF6.1* (MSAP-2230)], ATP binding [*PmTCP1* (AFLP-1113)], NAD biosynthetic process [*PmPEPC-LIKE* (MSAP-2760)], *S*-adenosylmethionine-dependent methyltransferase activity [*PmPMT7* (AFLP-1834)], and transcription factor activity. These genes may be essential for plant morphology, development, and energy metabolism in *P*. *mume*.

## Discussion

Many epimutations have been revealed to influence phenotypes in plants (Cubas et al., 1999; Manning et al., 2006; Seymour et al., 2008) and advances have been made in the emerging subfield of population epigenetics, which addresses questions about the prevalence and importance of epigenetic variation in the natural world (Richards, 2008). However, little is known about epialleles at the population level and their mechanism of inheritance in relation to genetics; that is, whether it occurs randomly or non-randomly (Massicotte et al., 2011; Becker and Weigel, 2012), whether epialleles can be retained or revert to their original state in response to changes in the environment or selection, and what substantial functions do they perform. In this study, we aimed to uncover the relationships between epigenetics and genetics, environment, and leaf shape traits, in the ornamental tree *P*. *mume* on basis of: (i) Leaves from 96 accessions were harvested from the 1a branches on 11–21 September 2013, and meteorological data were collected from 01 September 2012 to 31 August 2013 that overlapped with the main growth period of 1a branches and leaves, to minimize statistical errors. (ii) The sample individuals widely distributed in China were separated into three populations by a Bayesian clustering test using AFLP markers within STRUCTURE v.2.3.4. (iii) Leaf traits, important elements for biomass, displayed normal distribution and were examined significant different among the three populations.

### Cooperativity of Epigenetic and Genetic Structures Were Determined

Wild *P*. *mume* is distributed across a wide stretch of mainland China and little is presently known about the epigenetic diversity or structure in the wild population, although the parameters of genetic diversity and structure are well known in cultivated varieties (Yang et al., 2007; Shen et al., 2011; Sun et al., 2014; Zhang J. et al., 2015; Zhang et al., 2017). In this study, we used cultivated plants and wild individuals from southwest and southeast China to reveal the genetic and epigenetic diversity in this species. We showed that the genetic diversity of 96 accessions was lower than the epigenetic diversity, and that the epigenetic diversity differed significantly among the three populations, which suggested that epigenetics might contribute more than genetics to variation among populations, although some information on the epigenetic diversity represented by the pattern of total methylcytosine was missed by summing hemi-methylation and full methylation based on MSAP markers. The gene flow parameter showed strong gene exchange for the cultivated population, as expected, during artificial domestication and selection based on AFLP markers. Our findings indicated that the epigenetic diversity was not quite consistent with the genetic diversity.

We also investigated the genetic and epigenetic structures among the three populations of *P*. *mume* and used statistical analyses to reveal that the genetic *β_ST_* value (0.060) was higher than the epigenetic one (0.025), which suggested that the genetic variation resulted more strongly from population grouping than from epigenetic variation. This result is similar to findings reported previously for populations of two salt marsh perennials (Foust et al., 2016); however, opposite results were obtained in oak populations (Platt et al., 2015). Thus, the controversy over whether epigenetic changes are dependent on genetic variation remains. At a genome-wide scale, genetic and epigenetic variance were found to perform as one synergistic structure based on AFLP and MSAP markers, as revealed using co-inertia analysis. Further, the AFLP and MSAP markers, which provided a genome-wide snapshot of epigenetic and genetic variations, had a similar spatial distribution. The existence of a link between epigenetic and genetic variations is still unclear because information about the heredity of epigenetic loci and their relationship with genetic loci has been obtained only for a few populations. One perspective is that DNA methylation could be a source of random variation in natural populations (Massicotte et al., 2011). In contrast, Becker and Weigel (2012) suggested that DNA methylation patterns do not fluctuate randomly from one generation to the next, but neither are they completely stable. To resolve these issues, we believe that sequencing epiloci and determining of the rules regarding the presence or absence of a link between epigenetic and genetic variations offer the best approach.

### Linear Correlations between Relative Methylcytosine Levels and Leaf Traits

The patterns and levels of methylcytosine are considered to differ among different plants, within a single species at different developmental stages, and in different environments (Messeguer et al., 1991; Xiong et al., 1999; Hauben et al., 2009). The relative methylcytosine levels in the *P*. *mume* genome were calculated as the ratio of the number of patterns to the total bands. The relative total methylation level was 29.80%, and the relative total methylation levels differed significantly among the three populations. In perennial woody plant *Populus tomentosa* genomes, relative methylcytosine levels were 26.57% (natural populations) and 17.86% (F1 population) (Ma et al., 2012, 2013), and in *Populus simonii*, the relative methylcytosine level was 26.61% (Ci et al., 2015). In the model plant *Arabidopsis thaliana*, 32.4% of cytosines were found to be methylated in a genome-wide survey, including 24% CG methylation, 6.7% CHG methylation, and 1.7% CHH methylation (Jacobsen, 2011). Accordingly, a high level of methylcytosine is present in the genomes of higher plants.

Evidence has shown that an increase or decrease of DNA methylation level may affect gene expression and result in various phenotypic changes, and genes methylated in transcribed regions are highly expressed and constitutively active (Zhang et al., 2008). We found that leaf length, width, and area of *P*. *mume* were positively correlated with relative full and total methylation levels, which is similar to the previous finding that DNA methylation was positively correlated with growth traits and photosynthetic characteristics in poplar (Gourcilleau et al., 2010; Ma et al., 2012; Ci et al., 2015).

### Both Alleles and Epialleles Induce Variety of Leaf Traits

Linkage and association analyses are mature statistical techniques that are used to understand genetic markers and their function for molecular-assisted selective breeding in hybridization and natural populations (Ci et al., 2015; Ye et al., 2015). In *P. mume*, 120 simple sequence repeats (SSRs) and 1,484 single nucleotide polymorphisms (SNPs) that explaining 3–12% of the phenotypic variance of growth and leaf traits were detected (Sun et al., 2014). And by using SLAF-seq and whole-genome re-sequencing techniques, Zhang J. et al. (2015) and Zhang et al. (2017) successfully constructed a high-density genetic map for an F1 population and located a region on linkage group 7 that was strongly responsible for the weeping trait, and its functional effect was also analyzed. In poplar, although they ignored actual genetic relationships between individuals or metastable property of epigenetics in populations, Ma et al. (2012) and Ci et al. (2015) provided a new strategy for identifying epimarkers linked or associated with traits. However, the parameters relevant to both of the genetic and epigenetic markers were also ignored. We compared the uniformity of genetic and epigenetic structures and found they gave a cooperative structure that could be used to consider the genetic relationship, but not intergenerational relationships, between individuals of wild and cultivated *P*. *mume*. Therefore, the Q-matrix and K-matrix parameters generated from the AFLP markers were shared by both genetic and epigenetic associations, thereby avoiding errors caused by epigenetic changeability in structured populations for the first time. In total, 203 genetic and 423 epigenetic markers associated with leaf shape traits were detected, and 38 and 30 of them were sequenced, respectively.

Homology analysis and function prediction indicated that the variations in leaf traits were induced by both allelic and epiallelic candidate genes that participate in morphological development, metabolism, stress defense, signal transduction, and molecular transport, and encode transcription factors. Some unannotated sequences were also found. Some of the candidate genes, including some that encode organellar protein and methyltransferase activity protein, are discussed below.

Plant-specific *LATERAL ORGAN BOUNDARIES DOMAIN (LBD)* is a key regulator of plant organ development. *ASYMMETRIC LEAVES1* (*AS1*) and *AS2*, two members of *LBD*, are required for the development of normal leaf polarity formation and shape, and for the repression of *KNOX* genes in the leaf (Semiarti et al., 2001; Lin et al., 2003; Xu et al., 2003). Ectopic expression of *LBD1-LIKE* gene leads to alterations in the size and morphology of leaves (Shuai et al., 2002; Evans, 2007). Thereby, the candidate marker-linked gene *PmLBD1-LIKE* may participate in leaf development. Interestingly, one of the marker-linked genes, *PmPMT7*, was predicted to encode an *S*-adenosylmethionine-dependent methyltransferase activity protein, which has been found to be located in the Golgi, for DNA methylation (Santi et al., 1983; Nikolovski et al., 2012). The detection of *PmPMT7* among the marker-linked genes suggests that the encoded methyltransferase may regulate the variation of genomic methylation producing phenotypic variants of *P. mume*.

Three of the marker-linked genes encode the transcription factors PmmTERF5, PmTIFY4B-LIKE and PmGATA5-LIKE, which respond to stress, perform roles in regulating chloroplast homeostasis, leaf size and shape, and plant height (Bi et al., 2005; White, 2006; Kleine, 2012; Robles et al., 2012; Chung and Sunter, 2014). The transcription termination factor (mTERF) was first detected in human mitochondrial (Kruse et al., 1989) and then characterized in *A. thaliana* in both mitochondria and chloroplasts (Babiychuk et al., 2011). In plant, it plays important roles in affecting communication among chloroplasts, mitochondria, and the nucleus and leading to changes in the steady-state concentration of nuclear gene transcripts (Meskauskiene et al., 2009; Quesada et al., 2011; Romani et al., 2015). The *mda1* (*mTERF defective in Arabidopsis1, mterf5*) mutants exhibited altered chloroplast morphology and plant growth, and reduced pigmentation of cotyledons, leaves, stems and sepals (Robles et al., 2012). Similarly, *mterf4* (*bsm, rug2*) and *mterf9* showed defective chloroplast development, which is likely to cause paleness, stunted growth, reduced mesophyll cell numbers and abnormalities in leaf development (Babiychuk et al., 2011; Quesada et al., 2011; Robles et al., 2015). And *mterf6-1* mutant, a defect in photosynthesis, is associated with reduced levels of photosystem subunits causing seedling lethality (Romani et al., 2015). The plant specific transcription factor TIFY, is classified into four subfamilies as TIFY, PPD, JAZ and ZML according to the different domain architectures (Yan et al., 2007; Melotto et al., 2008; Bai et al., 2011). Ye et al. (2009) identified 20 *TIFY* genes in rice genomes and found most of them were predominantly expressed in leaf and they displayed tempo-spatial expression patterns, suggesting that expression and function vary by stage of plant growth and development. And Zhang Z. et al. (2015) described that transcription of maize genes *ZmTIFY4, 5, 8, 26*, and *28* was induced, while transcription of *ZmTIFY16, 13, 24, 27, 18*, and *30* was suppressed in response to drought stress. In *Arabidopsis*, deletion of the *PEAPOD* (*PPD*) locus increases leaf lamina size and results in dome-shaped rather than flat leaves (White, 2006). While AtTIFY4B with three domains (PPD, TIFY, and CCT_2) conserved between homologs from different plant species takes part in host defense against geminiviruses (Chung and Sunter, 2014). Similarly, *AtGATA5-LIKE*, regulating chlorophyll synthesis carbon and nitrogen metabolism, affects leaf blade extension (Reyes et al., 2004; Bi et al., 2005). Therefore, we considered that the candidate marker-linked genes *PmmTERF5, PmTIFY4B-LIKE* and *PmGATA5-LIKE* may take part in the development of leaf shape traits of *P. mume*.

*TCP1*, encoding a TCP transcription factor and containing a basic helixloop-helix (bHLH) domain, may play roles in regulating flower organ symmetry and dwarfed plants (Cubas et al., 2001; Koyama et al., 2010). It was also found that the activation tagged locus, *tcp1-1D*, can suppress the defective phenotypes of *bri1-5* resulted in dwarfed transgenic plants similar to typical *BR* deficient mutants, or signaling defective mutants (An et al., 2011). And the promoter of *TCP1* is active in the cotyledonary petioles and the distal part of the expanding leaves, as well as the midrib region and the petiole (Guo et al., 2010). It seems that *PmTCP1* may regulate the growth of leaves of *P. mume*.

Other candidate marker-linked genes were also detected. *PmSHM2* and *PmPEPC-LIKE* may encode proteins involved in core metabolic functions of mitochondria (Heazlewood et al., 2004; Engel et al., 2011), and play crucial roles in modulating the balance of carbon and nitrogen metabolism, respectively (Shi et al., 2015). *PmCaMBPS* may play a role in response to environmental stimuli (Snedden and Fromm, 1998; Zielinski, 1998; Bouché et al., 2005) and *PmNPF6.1* may encode a protein that transports a wide variety of substrates (Leran et al., 2014).

### Relationships between Epigenetics and Environment, and Artificial Cultivation

Epigenetics plays an important role in regulating gene expression and can be shaped by the environment, which provides insight into processes that function at the population level. Studies have revealed a correlation of environmental factors with epigenetic variances (Lira-Medeiros et al., 2010; Foust et al., 2016) and an association with adaptive phenotypic plasticity (Nicotra et al., 2010, 2015; Ci et al., 2015). Our results showed there was a cooperative structure between epimarkers and environmental factors, suggesting that epigenetic variance could be induced through environmental shifts affecting the population of *P*. *mume*, a species that has been cultivated for more than 3,000 years. We used the cultivated individuals to detect the variance of epimarkers with the aim of discovering the relationship between epigenetic and breeding. According to recent studies, epigenetic variation could have a major role in improving breeding efficiency and strategies for crop improvement (Manning et al., 2006; Seymour et al., 2008; Mirouze and Paszkowski, 2011; Springer, 2013). It has also been proposed that epimutations associated with beneficial traits could be selected and stably inherited from one generation to the next mitotically and/or meiotically, free from environmental impact, as described by Cubas et al. (1999). With the significant variation between wild and cultivated populations in terms of epigenetic diversity and structure, and candidate epiallele which plays essential roles in the formation of trait variation, we suggest the epigenetic variation could be reconstructed and selected across the domestication and cultivation processes and it has significant meaning for molecular breeding.

## Conclusion

This study was established with the aim of investigating the relationships between epigenetic variance, and genetic variance, environment factors, and traits of *P*. *mume* using molecular markers, multivariable statistics, association analysis, as well as sequencing approaches. We found that epigenetic diversity was greater than genetic diversity in the three populations studied. The epigenetic structure and genetic structure, and environmental factors performed the similarly statistical units, respectively. It suggested that epigenetic variance was affected by both genetics and the environment. Importantly, linear correlation analysis showed that leaf traits were positively correlated with both relative full methylation and total methylation levels. After association analysis, cloning, and sequencing, 68 AFLP and MSAP candidate marker sequences were obtained, and their annotations indicated that some of these marker-linked genes were essential for leaf morphology development and metabolism. Our results imply that these markers may play critical roles in the establishment of leaf length, width, area, and ratio of length to width. These findings are significant for molecular-assisted selection (MAS) for ornamental plant improvement.

## Author Contributions

KM and QZ designed the experiments. LS, TC, HP, and JW collected the plant materials and measured leaf traits. KM performed and analyzed the molecular markers test. KM and QZ wrote the manuscript. LS, TC, HP, and JW provided suggestions for revision.

## Conflict of Interest Statement

The authors declare that the research was conducted in the absence of any commercial or financial relationships that could be construed as a potential conflict of interest.

## Footnotes

1 http://www.cma.gov.cn/2011qxfw/2011qsjgx/

2 https://www.ncbi.nlm.nih.gov/

3 https://phytozome.jgi.doe.gov/pz/portal.html
