# Supplementary material for: Epigenetic Variance, Performing Cooperative Structure with Genetics, Is Associated with Leaf Shape Traits in Widely Distributed Populations of Ornamental Tree Prunus mume
Source: Front Plant Sci. 2018 Jan 30;9:41. doi: 10.3389/fpls.2018.00041 (PMC5797549; doi:10.3389/fpls.2018.00041)
Supplement: Supplementary file 1 [file Table_1.DOC]

Supplementary Material

Epigenetic variance, performing cooperative structure with genetics, is associated with leaf shape traits in widely distributed populations of ornamental tree *Prunus mume*

**Kaifeng Ma, Lidan Sun, Tangren Cheng, Huitang Pan, Jia Wang, Qixiang Zhang***

*** Correspondence:** Qixiang Zhang: [zqxbjfu@126.com](mailto:zqxbjfu@126.com)

## Supplementary Figures


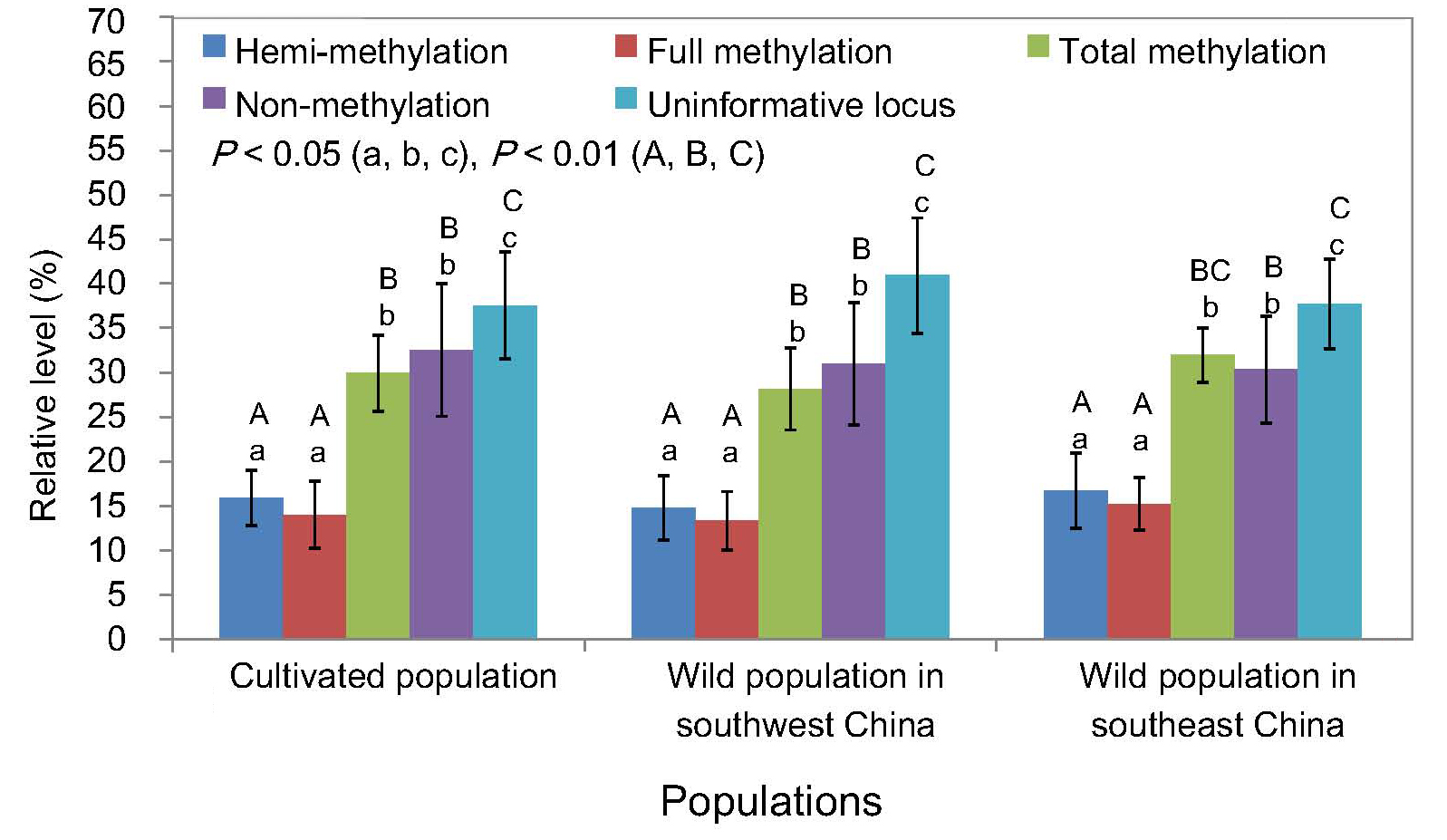


**Supplementary FIGURE 1 | Relative genomic methylation/non-methylation levels within each population of *Prunus mume*.** Pairwise comparison between relative methylation/non-methylation levels was performed by Wilcoxon’s rank-sum test. Within populations of southwest and southeast China, the relative total methylation levels of cultivated populations were higher than relative total methylation levels of wild populations (southwest China, χ^2^ = 6.32, *df* = 1, *P* <0.05; southeast China, χ^2^ = 5.22, *df* = 1, *P* <0.05), respectively.

##
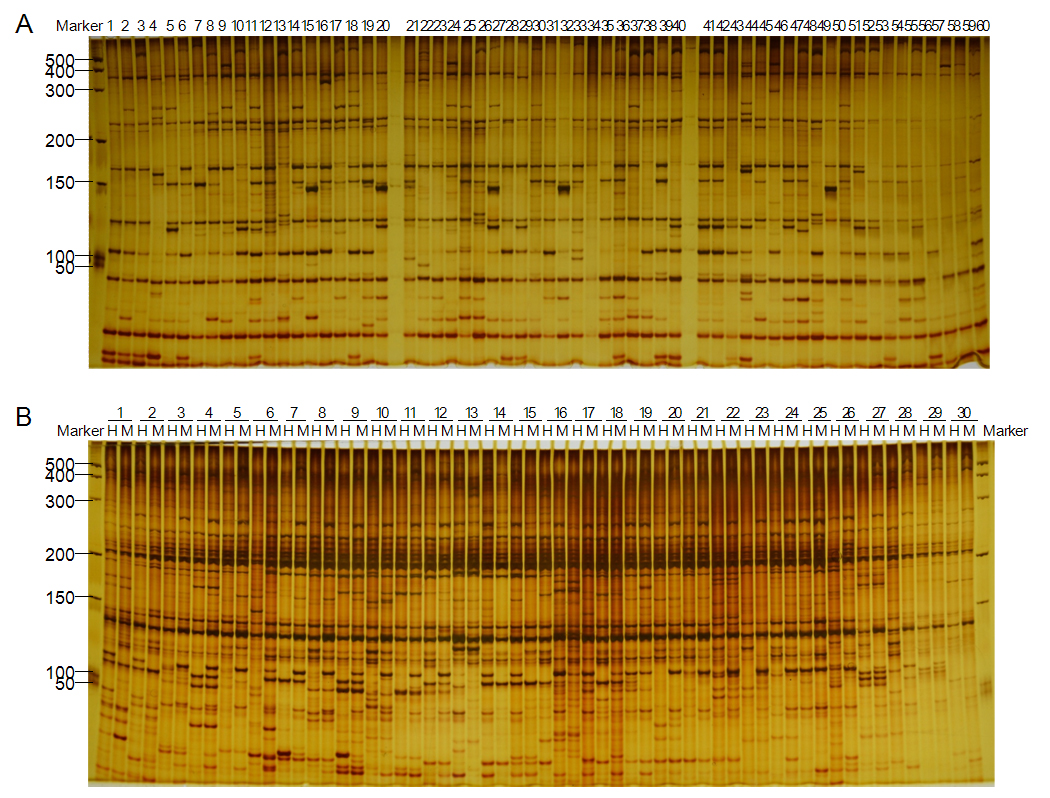


**Supplementary FIGURE 2 | Molecular bands monitored by polyacrylamide gel electrophoresis.** (**A**) AFLP markers generated with primer-pair combination E(AAC)-M(CTG) for selective amplification. The No. 1–60 stands for serial number of the plant sample. (**B**) MSAP markers generated with primer-pair combination E(AAC)-H/M(TCG) for selective amplification. The No. 1–30 stands for serial number of plant sample. H and M represent genomic DNA sample digested by *Eco*RI/*Hpa*II, and *Eco*RI*/Msp*I, respectively.

## Supplementary Tables

**Supplementary TABLE 1 | Environmental factors at the sample collection locations**.

| **No. ^a^** | **Collecting Sites** | **Denomination** | **Geographic coordinates** | | | **Meteorological data^b^** | | | | | | |
| --- | --- | --- | --- | --- | --- | --- | --- | --- | --- | --- | --- | --- |
|  |  |  | **Longitude** | **Latitude** | **Altitude** | **Average temperature** | **Average daily minimum temperature** | **Average daily highest temperature** | **Average relative humidity** | **Average minimum relative humidity** | **Total precipitation** | **Total sunshine time** |
|  |  |  | **(°, E)** | **(°, N)** | **(m)** | **(℃)** | **(℃)** | **(℃)** | **(%)** | **(%)** | **(mm)** | **(h)** |
| 1 | Wuhan, Hubei Province | *P. mume* ‘Shichujin’ | 114.402 | 30.543 | 25 | 17.2 | 12.9 | 22.8 | 77.0 | 49.7 | 1434.2 | 2092.5 |
|  |  |  |  |  |  | 30.6 | 26.6 | 35.7 | 69.2 | 46.4 | 136.0 | 281.7 |
| 2 | Wuhan, Hubei Province | *P. mume* ‘Suanzhu Taige’ | 114.402 | 30.542 | 25 | 17.2 | 12.9 | 22.8 | 77.0 | 49.7 | 1434.2 | 2092.5 |
|  |  |  |  |  |  | 30.6 | 26.6 | 35.7 | 69.2 | 46.4 | 136.0 | 281.7 |
| 3 | Wuhan, Hubei Province | *P. mume* ‘Hongfen Taige’ | 114.402 | 30.543 | 25 | 17.2 | 12.9 | 22.8 | 77.0 | 49.7 | 1434.2 | 2092.5 |
|  |  |  |  |  |  | 30.6 | 26.6 | 35.7 | 69.2 | 46.4 | 136.0 | 281.7 |
| 4 | Wuhan, Hubei Province | *P. mume* ‘Tiegan Zhusha’ | 114.402 | 30.542 | 25 | 17.2 | 12.9 | 22.8 | 77.0 | 49.7 | 1434.2 | 2092.5 |
|  |  |  |  |  |  | 30.6 | 26.6 | 35.7 | 69.2 | 46.4 | 136.0 | 281.7 |
| 5 | Wuhan, Hubei Province | *P. mume* ‘Feifei Zhusha’ | 114.402 | 30.542 | 25 | 17.2 | 12.9 | 22.8 | 77.0 | 49.7 | 1434.2 | 2092.5 |
|  |  |  |  |  |  | 30.6 | 26.6 | 35.7 | 69.2 | 46.4 | 136.0 | 281.7 |
| 6 | Wuhan, Hubei Province | *P. mume* ‘Taohong Taige’ | 114.403 | 30.543 | 25 | 17.2 | 12.9 | 22.8 | 77.0 | 49.7 | 1434.2 | 2092.5 |
|  |  |  |  |  |  | 30.6 | 26.6 | 35.7 | 69.2 | 46.4 | 136.0 | 281.7 |
| 7 | Wuhan, Hubei Province | *P. mume* ‘Fenkou’ | 114.402 | 30.543 | 25 | 17.2 | 12.9 | 22.8 | 77.0 | 49.7 | 1434.2 | 2092.5 |
|  |  |  |  |  |  | 30.6 | 26.6 | 35.7 | 69.2 | 46.4 | 136.0 | 281.7 |
| 8 | Wuhan, Hubei Province | *P. mume* ‘Hongyan Gongfen’ | 114.403 | 30.543 | 25 | 17.2 | 12.9 | 22.8 | 77.0 | 49.7 | 1434.2 | 2092.5 |
|  |  |  |  |  |  | 30.6 | 26.6 | 35.7 | 69.2 | 46.4 | 136.0 | 281.7 |
| 9 | Wuhan, Hubei Province | *P. mume* ‘Fuban Tiaozhi’ | 114.403 | 30.543 | 25 | 17.2 | 12.9 | 22.8 | 77.0 | 49.7 | 1434.2 | 2092.5 |
|  |  |  |  |  |  | 30.6 | 26.6 | 35.7 | 69.2 | 46.4 | 136.0 | 281.7 |
| 10 | Wuhan, Hubei Province | *P. mume* ‘Jinzhi Dongfang’ | 114.402 | 30.542 | 25 | 17.2 | 12.9 | 22.8 | 77.0 | 49.7 | 1434.2 | 2092.5 |
|  |  |  |  |  |  | 30.6 | 26.6 | 35.7 | 69.2 | 46.4 | 136.0 | 281.7 |
| 11 | Wuhan, Hubei Province | *P. mume* ‘Kaidi’ | 114.403 | 30.543 | 25 | 17.2 | 12.9 | 22.8 | 77.0 | 49.7 | 1434.2 | 2092.5 |
|  |  |  |  |  |  | 30.6 | 26.6 | 35.7 | 69.2 | 46.4 | 136.0 | 281.7 |
| 12 | Wuhan, Hubei Province | *P. mume* ‘Zhusha Wanzhaoshui’ | 114.402 | 30.542 | 25 | 17.2 | 12.9 | 22.8 | 77.0 | 49.7 | 1434.2 | 2092.5 |
|  |  |  |  |  |  | 30.6 | 26.6 | 35.7 | 69.2 | 46.4 | 136.0 | 281.7 |
| 13 | Wuhan, Hubei Province | *P. mume* ‘Gengsha’ | 114.402 | 30.542 | 25 | 17.2 | 12.9 | 22.8 | 77.0 | 49.7 | 1434.2 | 2092.5 |
|  |  |  |  |  |  | 30.6 | 26.6 | 35.7 | 69.2 | 46.4 | 136.0 | 281.7 |
| 14 | Wuhan, Hubei Province | *P. mume* ‘Nanjing Chuizhi’ | 114.402 | 30.542 | 25 | 17.2 | 12.9 | 22.8 | 77.0 | 49.7 | 1434.2 | 2092.5 |
|  |  |  |  |  |  | 30.6 | 26.6 | 35.7 | 69.2 | 46.4 | 136.0 | 281.7 |
| 15 | Wuhan, Hubei Province | *P. mume* ‘Yuzhiji’ | 114.402 | 30.542 | 25 | 17.2 | 12.9 | 22.8 | 77.0 | 49.7 | 1434.2 | 2092.5 |
|  |  |  |  |  |  | 30.6 | 26.6 | 35.7 | 69.2 | 46.4 | 136.0 | 281.7 |
| 16 | Wuhan, Hubei Province | *P. mume* ‘Yushan Yudie’ | 114.402 | 30.543 | 25 | 17.2 | 12.9 | 22.8 | 77.0 | 49.7 | 1434.2 | 2092.5 |
|  |  |  |  |  |  | 30.6 | 26.6 | 35.7 | 69.2 | 46.4 | 136.0 | 281.7 |
| 17 | Wuhan, Hubei Province | *P. mume* ‘Xuhe’ | 114.402 | 30.542 | 25 | 17.2 | 12.9 | 22.8 | 77.0 | 49.7 | 1434.2 | 2092.5 |
|  |  |  |  |  |  | 30.6 | 26.6 | 35.7 | 69.2 | 46.4 | 136.0 | 281.7 |
| 18 | Wuhan, Hubei Province | *P. mume* ‘Changrui Lv-e’ | 114.402 | 30.543 | 25 | 17.2 | 12.9 | 22.8 | 77.0 | 49.7 | 1434.2 | 2092.5 |
|  |  |  |  |  |  | 30.6 | 26.6 | 35.7 | 69.2 | 46.4 | 136.0 | 281.7 |
| 19 | Wuhan, Hubei Province | *P. mume* ‘Bingdi Taige Lv-e’ | 114.402 | 30.542 | 25 | 17.2 | 12.9 | 22.8 | 77.0 | 49.7 | 1434.2 | 2092.5 |
|  |  |  |  |  |  | 30.6 | 26.6 | 35.7 | 69.2 | 46.4 | 136.0 | 281.7 |
| 20 | Wuhan, Hubei Province | *P. mume* ‘Jingzhe Mei’ | 114.402 | 30.542 | 25 | 17.2 | 12.9 | 22.8 | 77.0 | 49.7 | 1434.2 | 2092.5 |
|  |  |  |  |  |  | 30.6 | 26.6 | 35.7 | 69.2 | 46.4 | 136.0 | 281.7 |
| 21 | Wuhan, Hubei Province | *P. mume* ‘Moshan Shuizhusha’ | 114.402 | 30.542 | 25 | 17.2 | 12.9 | 22.8 | 77.0 | 49.7 | 1434.2 | 2092.5 |
|  |  |  |  |  |  | 30.6 | 26.6 | 35.7 | 69.2 | 46.4 | 136.0 | 281.7 |
| 22 | Wuhan, Hubei Province | *P. mume* ‘Nanjing Fuhuangxiang’ | 114.402 | 30.541 | 25 | 17.2 | 12.9 | 22.8 | 77.0 | 49.7 | 1434.2 | 2092.5 |
|  |  |  |  |  |  | 30.6 | 26.6 | 35.7 | 69.2 | 46.4 | 136.0 | 281.7 |
| 23 | Kunming, Yunnan Province | *P. mume* ‘Qingxin’ | 102.747 | 25.141 | 1890 | 16.0 | 11.3 | 22.3 | 67.7 | 39.2 | 804.7 | 2512.5 |
|  |  |  |  |  |  | 19.9 | 16.8 | 25.3 | 79.1 | 51.0 | 153.9 | 175.2 |
| 24 | Kunming, Yunnan Province | *P. mume* ‘Fenzhu’ | 102.748 | 25.14 | 1890 | 16.0 | 11.3 | 22.3 | 67.7 | 39.2 | 804.7 | 2512.5 |
|  |  |  |  |  |  | 19.9 | 16.8 | 25.3 | 79.1 | 51.0 | 153.9 | 175.2 |
| 25 | Kunming, Yunnan Province | *P. mume* ‘Taige Gongfen’ | 102.748 | 25.14 | 1890 | 16.0 | 11.3 | 22.3 | 67.7 | 39.2 | 804.7 | 2512.5 |
|  |  |  |  |  |  | 19.9 | 16.8 | 25.3 | 79.1 | 51.0 | 153.9 | 175.2 |
| 26 | Kunming, Yunnan Province | *P. mume* ‘Lijiang Zhaoshuimei’ | 102.748 | 25.14 | 1890 | 16.0 | 11.3 | 22.3 | 67.7 | 39.2 | 804.7 | 2512.5 |
|  |  |  |  |  |  | 19.9 | 16.8 | 25.3 | 79.1 | 51.0 | 153.9 | 175.2 |
| 27 | Kunming, Yunnan Province | *P. mume* ‘Yuefen’ | 102.749 | 25.141 | 1890 | 16.0 | 11.3 | 22.3 | 67.7 | 39.2 | 804.7 | 2512.5 |
|  |  |  |  |  |  | 19.9 | 16.8 | 25.3 | 79.1 | 51.0 | 153.9 | 175.2 |
| 28 | Kunming, Yunnan Province | *P. mume* ‘Danyun Gongfen’ | 102.747 | 25.14 | 1890 | 16.0 | 11.3 | 22.3 | 67.7 | 39.2 | 804.7 | 2512.5 |
|  |  |  |  |  |  | 19.9 | 16.8 | 25.3 | 79.1 | 51.0 | 153.9 | 175.2 |
| 29 | Kunming, Yunnan Province | *P. mume* ‘Longtanfen’ | 102.748 | 25.138 | 1890 | 16.0 | 11.3 | 22.3 | 67.7 | 39.2 | 804.7 | 2512.5 |
|  |  |  |  |  |  | 19.9 | 16.8 | 25.3 | 79.1 | 51.0 | 153.9 | 175.2 |
| 30 | Kunming, Yunnan Province | *P. mume* ‘Qianyin Gongfen’ | 102.747 | 25.139 | 1890 | 16.0 | 11.3 | 22.3 | 67.7 | 39.2 | 804.7 | 2512.5 |
|  |  |  |  |  |  | 19.9 | 16.8 | 25.3 | 79.1 | 51.0 | 153.9 | 175.2 |
| 31 | Kunming, Yunnan Province | *P. mume* ‘Huairou Baozi’ | 102.747 | 25.139 | 1890 | 16.0 | 11.3 | 22.3 | 67.7 | 39.2 | 804.7 | 2512.5 |
|  |  |  |  |  |  | 19.9 | 16.8 | 25.3 | 79.1 | 51.0 | 153.9 | 175.2 |
| 32 | Kunming, Yunnan Province | *P. mume* ‘Caoxi Gongfen’ | 102.748 | 25.141 | 1890 | 16.0 | 11.3 | 22.3 | 67.7 | 39.2 | 804.7 | 2512.5 |
|  |  |  |  |  |  | 19.9 | 16.8 | 25.3 | 79.1 | 51.0 | 153.9 | 175.2 |
| 33 | Zhao'an, Fujian Province | *P. mume* ‘Qingzhu Mei’ | 117.054 | 23.855 | 65 | 22.1 | 18.9 | 26.7 | 67.7 | 47.2 | 1843.5 | 1742.3 |
|  |  |  |  |  |  | 29.0 | 25.6 | 33.8 | 70.9 | 47.8 | 390.0 | 199.6 |
| 34 | Zhao'an, Fujian Province | *P. mume* ‘Baifen Mei’ | 117.054 | 23.855 | 63 | 22.1 | 18.9 | 26.7 | 67.7 | 47.2 | 1843.5 | 1742.3 |
|  |  |  |  |  |  | 29.0 | 25.6 | 33.8 | 70.9 | 47.8 | 390.0 | 199.6 |
| 35 | Yongtai, Fujian Province | *P. mume* ‘Yangshi Mei’ | 118.967 | 25.856 | 44 | 20.4 | 17.4 | 25.2 | 71.6 | 51.9 | 1137.5 | 1578.4 |
|  |  |  |  |  |  | 29.6 | 26.1 | 34.9 | 72.0 | 50.6 | 139.5 | 196.7 |
| 36 | Yongtai, Fujian Province | *P. mume* ‘Longyan Mei’ | 118.967 | 25.856 | 44 | 20.4 | 17.4 | 25.2 | 71.6 | 51.9 | 1137.5 | 1578.4 |
|  |  |  |  |  |  | 29.6 | 26.1 | 34.9 | 72.0 | 50.6 | 139.5 | 196.7 |
| 37 | Yongtai, Fujian Province | *P. mume* ‘Renli’ | 118.967 | 25.857 | 64 | 20.4 | 17.4 | 25.2 | 71.6 | 51.9 | 1137.5 | 1578.4 |
|  |  |  |  |  |  | 29.6 | 26.1 | 34.9 | 72.0 | 50.6 | 139.5 | 196.7 |
| 38 | Yongtai, Fujian Province | *P. mume* ‘Jinchai’ | 118.968 | 25.857 | 71 | 20.4 | 17.4 | 25.2 | 71.6 | 51.9 | 1137.5 | 1578.4 |
|  |  |  |  |  |  | 29.6 | 26.1 | 34.9 | 72.0 | 50.6 | 139.5 | 196.7 |
| 39 | Yongtai, Fujian Province | *P. mume* ‘Dayeqing’ | 118.968 | 25.857 | 67 | 20.4 | 17.4 | 25.2 | 71.6 | 51.9 | 1137.5 | 1578.4 |
|  |  |  |  |  |  | 29.6 | 26.1 | 34.9 | 72.0 | 50.6 | 139.5 | 196.7 |
| 40 | Meizhou, Guangdong Province | *P. mume* ‘Luogang No.1’ | 116.377 | 24.332 | 228 | 21.3 | 17.5 | 26.6 | 73.1 | 49.8 | 1930.2 | 1827.8 |
|  |  |  |  |  |  | 27.8 | 24.3 | 32.8 | 78.0 | 54.2 | 418.7 | 184.1 |
| 41 | Meizhou, Guangdong Province | *P. mume* ‘Luogang No.2’ | 116.377 | 24.332 | 228 | 21.3 | 17.5 | 26.6 | 73.1 | 49.8 | 1930.2 | 1827.8 |
|  |  |  |  |  |  | 27.8 | 24.3 | 32.8 | 78.0 | 54.2 | 418.7 | 184.1 |
| 42 | Meizhou, Guangdong Province | *P. mume* ‘Luogang Texuan’ | 116.377 | 24.332 | 225 | 21.3 | 17.5 | 26.6 | 73.1 | 49.8 | 1930.2 | 1827.8 |
|  |  |  |  |  |  | 27.8 | 24.3 | 32.8 | 78.0 | 54.2 | 418.7 | 184.1 |
| 43 | Meizhou, Guangdong Province | *P. mume* ‘E su’ | 116.377 | 24.332 | 228 | 21.3 | 17.5 | 26.6 | 73.1 | 49.8 | 1930.2 | 1827.8 |
|  |  |  |  |  |  | 27.8 | 24.3 | 32.8 | 78.0 | 54.2 | 418.7 | 184.1 |
| 44 | Meizhou, Guangdong Province | *P. mume* ‘Jichunqing’ | 116.377 | 24.332 | 228 | 21.3 | 17.5 | 26.6 | 73.1 | 49.8 | 1930.2 | 1827.8 |
|  |  |  |  |  |  | 27.8 | 24.3 | 32.8 | 78.0 | 54.2 | 418.7 | 184.1 |
| 45 | Meizhou, Guangdong Province | *P. mume* ‘Chaotang Gongfen’ | 116.169 | 24.362 | 412 | 21.3 | 17.5 | 26.6 | 73.1 | 49.8 | 1930.2 | 1827.8 |
|  |  |  |  |  |  | 27.8 | 24.3 | 32.8 | 78.0 | 54.2 | 418.7 | 184.1 |
| 46 | Meizhou, Guangdong Province | *P. mume* ‘Fanshu mei’ | 116.131 | 24.33 | 111 | 21.3 | 17.5 | 26.6 | 73.1 | 49.8 | 1930.2 | 1827.8 |
|  |  |  |  |  |  | 27.8 | 24.3 | 32.8 | 78.0 | 54.2 | 418.7 | 184.1 |
| 47 | Meizhou, Guangdong Province | *P. mume* ‘Dahe Qing’ | 116.18 | 24.683 | 162 | 21.3 | 17.5 | 26.6 | 73.1 | 49.8 | 1930.2 | 1827.8 |
|  |  |  |  |  |  | 27.8 | 24.3 | 32.8 | 78.0 | 54.2 | 418.7 | 184.1 |
| 48 | Meizhou, Guangdong Province | *P. mume* ‘Zaoshu Baifen Mei’ | 116.182 | 24.682 | 158 | 21.3 | 17.5 | 26.6 | 73.1 | 49.8 | 1930.2 | 1827.8 |
|  |  |  |  |  |  | 27.8 | 24.3 | 32.8 | 78.0 | 54.2 | 418.7 | 184.1 |
| 49 | Meizhou, Guangdong Province | *P. mume* ‘Taohong Gongfen’ | 116.112 | 24.303 | 87 | 21.3 | 17.5 | 26.6 | 73.1 | 49.8 | 1930.2 | 1827.8 |
|  |  |  |  |  |  | 27.8 | 24.3 | 32.8 | 78.0 | 54.2 | 418.7 | 184.1 |
| 50 | Pu'ning, Guangdong Province | *P. mume* ‘Yingzhi Dalimei’ | 115.979 | 23.159 | 39 | 21.3 | 18.0 | 26.5 | 77.6 | 54.7 | 2831.4 | 1855.2 |
|  |  |  |  |  |  | 27.3 | 24.1 | 32.2 | 85.5 | 62.5 | 952.7 | 179.2 |
| 51 | Pu'ning, Guangdong Province | *P. mume* ‘Ruanzhi Dalimei’ | 115.979 | 23.16 | 37 | 21.3 | 18.0 | 26.5 | 77.6 | 54.7 | 2831.4 | 1855.2 |
|  |  |  |  |  |  | 27.3 | 24.1 | 32.2 | 85.5 | 62.5 | 952.7 | 179.2 |
| 52 | Pu'ning, Guangdong Province | *P. mume* ‘Henghe’ | 115.979 | 23.16 | 35 | 21.3 | 18.0 | 26.5 | 77.6 | 54.7 | 2831.4 | 1855.2 |
|  |  |  |  |  |  | 27.3 | 24.1 | 32.2 | 85.5 | 62.5 | 952.7 | 179.2 |
| 53 | Pu'ning, Guangdong Province | *P. mume* ‘Ai-baimei’ | 115.979 | 23.16 | 36 | 21.3 | 18.0 | 26.5 | 77.6 | 54.7 | 2831.4 | 1855.2 |
|  |  |  |  |  |  | 27.3 | 24.1 | 32.2 | 85.5 | 62.5 | 952.7 | 179.2 |
| 54 | Nan'an District, Chongqing | *P. mume* ‘Midan lv’ | 106.629 | 29.557 | 465 | 19.9 | 16.9 | 24.2 | 71.0 | 51.3 | 1026.9 | 1097.3 |
|  |  |  |  |  |  | 30.5 | 26.5 | 36.3 | 59.2 | 38.1 | 62.6 | 209.2 |
| 55 | Nan'an District, Chongqing | *P. mume* ‘Xiao Lv-e’ | 106.629 | 29.557 | 465 | 19.9 | 16.9 | 24.2 | 71.0 | 51.3 | 1026.9 | 1097.3 |
|  |  |  |  |  |  | 30.5 | 26.5 | 36.3 | 59.2 | 38.1 | 62.6 | 209.2 |
| 56 | Nan'an District, Chongqing | *P. mume* ‘Chuanxi Xiaofen’ | 106.629 | 29.557 | 465 | 19.9 | 16.9 | 24.2 | 71.0 | 51.3 | 1026.9 | 1097.3 |
|  |  |  |  |  |  | 30.5 | 26.5 | 36.3 | 59.2 | 38.1 | 62.6 | 209.2 |
| 57 | Nan'an District, Chongqing | *P. mume* ‘Zididan’ | 106.629 | 29.557 | 465 | 19.9 | 16.9 | 24.2 | 71.0 | 51.3 | 1026.9 | 1097.3 |
|  |  |  |  |  |  | 30.5 | 26.5 | 36.3 | 59.2 | 38.1 | 62.6 | 209.2 |
| 58 | Nan'an District, Chongqing | *P. mume* ‘Zhusha’ | 106.63 | 29.557 | 462 | 19.9 | 16.9 | 24.2 | 71.0 | 51.3 | 1026.9 | 1097.3 |
|  |  |  |  |  |  | 30.5 | 26.5 | 36.3 | 59.2 | 38.1 | 62.6 | 209.2 |
| 59 | Shexian, Anhui Province | *P. mume* ‘Lv Mei’ | 118.372 | 30.001 | 320 | 17.5 | 13.2 | 23.3 | 70.0 | 43.7 | 1647.9 | 1931.7 |
|  |  |  |  |  |  | 30.0 | 25.4 | 36.3 | 61.5 | 37.0 | 59.8 | 235.2 |
| 60 | Kunming, Yunnan Province | *P. mume* ‘Kunming 1’ | 102.748 | 25.141 | 1890 | 16.0 | 11.3 | 22.3 | 67.7 | 39.2 | 804.7 | 2512.5 |
|  |  |  |  |  |  | 19.9 | 16.8 | 25.3 | 79.1 | 51.0 | 153.9 | 175.2 |
| 61 | Kunming, Yunnan Province | *P. mume* ‘Kunming 2’ | 102.747 | 25.14 | 1890 | 16.0 | 11.3 | 22.3 | 67.7 | 39.2 | 804.7 | 2512.5 |
|  |  |  |  |  |  | 19.9 | 16.8 | 25.3 | 79.1 | 51.0 | 153.9 | 175.2 |
| 62 | Weining, Guizhou Province | *P. mume* ‘Weining 1’ | 104.279 | 26.795 | 2292 | 11.6 | 8.2 | 17.2 | 75.7 | 50.4 | 762.3 | 1833.1 |
|  |  |  |  |  |  | 17.8 | 15.1 | 22.5 | 79.2 | 55.8 | 120.3 | 154.7 |
| 63 | Weining, Guizhou Province | *P. mume* ‘Weining 2’ | 104.279 | 26.795 | 2292 | 11.6 | 8.2 | 17.2 | 75.7 | 50.4 | 762.3 | 1833.1 |
|  |  |  |  |  |  | 17.8 | 15.1 | 22.5 | 79.2 | 55.8 | 120.3 | 154.7 |
| 64 | Hezhang, Guizhou Province | *P. mume* ‘Hezhang 1’ | 104.708 | 27.235 | 1618 | 14.2 | 10.8 | 19.4 | 75.1 | 52.3 | 782.5 | 1338.6 |
|  |  |  |  |  |  | 22.2 | 18.5 | 28.1 | 73.0 | 46.6 | 153.1 | 155.7 |
| 65 | Hezhang, Guizhou Province | *P. mume* ‘Hezhang 2’ | 104.708 | 27.235 | 1618 | 14.2 | 10.8 | 19.4 | 75.1 | 52.3 | 782.5 | 1338.6 |
|  |  |  |  |  |  | 22.2 | 18.5 | 28.1 | 73.0 | 46.6 | 153.1 | 155.7 |
| 66 | Hezhang, Guizhou Province | *P. mume* ‘Hezhang 3’ | 104.708 | 27.235 | 1618 | 14.2 | 10.8 | 19.4 | 75.1 | 52.3 | 782.5 | 1338.6 |
|  |  |  |  |  |  | 22.2 | 18.5 | 28.1 | 73.0 | 46.6 | 153.1 | 155.7 |
| 67 | Hezhang, Guizhou Province | *P. mume* ‘Hezhang 4’ | 104.708 | 27.235 | 1618 | 14.2 | 10.8 | 19.4 | 75.1 | 52.3 | 782.5 | 1338.6 |
|  |  |  |  |  |  | 22.2 | 18.5 | 28.1 | 73.0 | 46.6 | 153.1 | 155.7 |
| 68 | Hezhang, Guizhou Province | *P. mume* ‘Hezhang 5’ | 104.708 | 27.235 | 1618 | 14.2 | 10.8 | 19.4 | 75.1 | 52.3 | 782.5 | 1338.6 |
|  |  |  |  |  |  | 22.2 | 18.5 | 28.1 | 73.0 | 46.6 | 153.1 | 155.7 |
| 69 | Hezhang, Guizhou Province | *P. mume* ‘Hezhang 6’ | 104.708 | 27.235 | 1618 | 14.2 | 10.8 | 19.4 | 75.1 | 52.3 | 782.5 | 1338.6 |
|  |  |  |  |  |  | 22.2 | 18.5 | 28.1 | 73.0 | 46.6 | 153.1 | 155.7 |
| 70 | Libo, Guizhou Province | *P. mume* ‘Libo 1’ | 108.013 | 25.293 | 572 | 19.0 | 15.7 | 24.3 | 75.4 | 52.0 | 1027.1 | 1218.6 |
|  |  |  |  |  |  | 26.4 | 23.1 | 32.1 | 79.1 | 52.9 | 143.5 | 147.0 |
| 71 | Libo, Guizhou Province | *P. mume* ‘Libo 2’ | 108.005 | 25.293 | 616 | 19.0 | 15.7 | 24.3 | 75.4 | 52.0 | 1027.1 | 1218.6 |
|  |  |  |  |  |  | 26.4 | 23.1 | 32.1 | 79.1 | 52.9 | 143.5 | 147.0 |
| 72 | Muli, Sichuan Province | *P. mume* ‘Muli 1’ | 101.276 | 27.919 | 2238 | 14.1 | 8.5 | 21.5 | 50.2 | 26.9 | 823.5 | 2464.0 |
|  |  |  |  |  |  | 18.9 | 14.3 | 25.7 | 64.4 | 35.8 | 88.7 | 191.8 |
| 73 | Muli, Sichuan Province | *P. mume* ‘Muli 2’ | 101.277 | 27.942 | 2211 | 14.1 | 8.5 | 21.5 | 50.2 | 26.9 | 823.5 | 2464.0 |
|  |  |  |  |  |  | 18.9 | 14.3 | 25.7 | 64.4 | 35.8 | 88.7 | 191.8 |
| 74 | Muli, Sichuan Province | *P. mume* ‘Muli 3’ | 101.278 | 27.943 | 2224 | 14.1 | 8.5 | 21.5 | 50.2 | 26.9 | 823.5 | 2464.0 |
|  |  |  |  |  |  | 18.9 | 14.3 | 25.7 | 64.4 | 35.8 | 88.7 | 191.8 |
| 75 | Muli, Sichuan Province | *P. mume* ‘Muli 4’ | 101.274 | 27.932 | 2375 | 14.1 | 8.5 | 21.5 | 50.2 | 26.9 | 823.5 | 2464.0 |
|  |  |  |  |  |  | 18.9 | 14.3 | 25.7 | 64.4 | 35.8 | 88.7 | 191.8 |
| 76 | Muli, Sichuan Province | *P. mume* ‘Muli 5’ | 101.275 | 27.932 | 2370 | 14.1 | 8.5 | 21.5 | 50.2 | 26.9 | 823.5 | 2464.0 |
|  |  |  |  |  |  | 18.9 | 14.3 | 25.7 | 64.4 | 35.8 | 88.7 | 191.8 |
| 77 | Mianning, Sichuan Province | *P. mume* ‘Mianning 1’ | 101.935 | 28.332 | 2022 | 14.0 | 9.3 | 21.3 | 70.8 | 40.8 | 1021.8 | 1841.4 |
|  |  |  |  |  |  | 21.8 | 16.9 | 29.4 | 74.6 | 39.0 | 106.2 | 189.3 |
| 78 | Mianning, Sichuan Province | *P. mume* ‘Mianning 2’ | 101.898 | 28.296 | 1701 | 14.0 | 9.3 | 21.3 | 70.8 | 40.8 | 1021.8 | 1841.4 |
|  |  |  |  |  |  | 21.8 | 16.9 | 29.4 | 74.6 | 39.0 | 106.2 | 189.3 |
| 79 | Mianning, Sichuan Province | *P. mume* ‘Mianning 3’ | 101.937 | 28.284 | 2356 | 14.0 | 9.3 | 21.3 | 70.8 | 40.8 | 1021.8 | 1841.4 |
|  |  |  |  |  |  | 21.8 | 16.9 | 29.4 | 74.6 | 39.0 | 106.2 | 189.3 |
| 80 | Mianning, Sichuan Province | *P. mume* ‘Mianning 4’ | 101.936 | 28.285 | 2325 | 14.0 | 9.3 | 21.3 | 70.8 | 40.8 | 1021.8 | 1841.4 |
|  |  |  |  |  |  | 21.8 | 16.9 | 29.4 | 74.6 | 39.0 | 106.2 | 189.3 |
| 81 | Bomi, Tibet | *P. mume* ‘Tongmai 1’ | 116.447 | 40.152 | 50 | 12.9 | 8.2 | 18.1 | 55.2 | 33.9 | 579.1 | 2371.1 |
|  |  |  |  |  |  | 17.5 | 12.3 | 26.3 | 71.6 | 34.9 | 75.2 | 188.6 |
| 82 | Bomi, Tibet | *P. mume* ‘Tongmai 2’ | 116.447 | 40.152 | 50 | 12.9 | 8.2 | 18.1 | 55.2 | 33.9 | 579.1 | 2371.1 |
|  |  |  |  |  |  | 17.5 | 12.3 | 26.3 | 71.6 | 34.9 | 75.2 | 188.6 |
| 83 | Yongtai, Fujian Province | *P. mume* ‘Yongtai 1’ | 118.961 | 25.753 | 76 | 20.4 | 17.4 | 25.2 | 71.6 | 51.9 | 1137.5 | 1578.4 |
|  |  |  |  |  |  | 29.6 | 26.1 | 34.9 | 72.0 | 50.6 | 139.5 | 196.7 |
| 84 | Meizhou, Guangdong Province | *P. mume* ‘Meizhou 1’ | 116.115 | 24.289 | 85 | 21.3 | 17.5 | 26.6 | 73.1 | 49.8 | 1930.2 | 1827.8 |
|  |  |  |  |  |  | 27.8 | 24.3 | 32.8 | 78.0 | 54.2 | 418.7 | 184.1 |
| 85 | Meizhou, Guangdong Province | *P. mume* ‘Meizhou 2’ | 116.131 | 24.33 | 112 | 21.3 | 17.5 | 26.6 | 73.1 | 49.8 | 1930.2 | 1827.8 |
|  |  |  |  |  |  | 27.8 | 24.3 | 32.8 | 78.0 | 54.2 | 418.7 | 184.1 |
| 86 | Huangshan, Anhui Province | *P. mume* ‘Huangshan 1’ | 118.168 | 30.108 | 855 | 9.3 | 6.6 | 12.7 | 73.1 | 52.2 | 2365.0 | 1933.0 |
|  |  |  |  |  |  | 19.4 | 17.3 | 23.0 | 85.0 | 67.8 | 187.7 | 167.9 |
| 87 | Huangshan, Anhui Province | *P. mume* ‘Huangshan 2’ | 118.168 | 30.108 | 845 | 9.3 | 6.6 | 12.7 | 73.1 | 52.2 | 2365.0 | 1933.0 |
|  |  |  |  |  |  | 19.4 | 17.3 | 23.0 | 85.0 | 67.8 | 187.7 | 167.9 |
| 88 | Huangshan, Anhui Province | *P. mume* ‘Huangshan 3’ | 118.167 | 30.108 | 836 | 9.3 | 6.6 | 12.7 | 73.1 | 52.2 | 2365.0 | 1933.0 |
|  |  |  |  |  |  | 19.4 | 17.3 | 23.0 | 85.0 | 67.8 | 187.7 | 167.9 |
| 89 | Huangshan, Anhui Province | *P. mume* ‘Huangshan 4’ | 118.167 | 30.108 | 825 | 9.3 | 6.6 | 12.7 | 73.1 | 52.2 | 2365.0 | 1933.0 |
|  |  |  |  |  |  | 19.4 | 17.3 | 23.0 | 85.0 | 67.8 | 187.7 | 167.9 |
| 90 | Huangshan, Anhui Province | *P. mume* ‘Huangshan 5’ | 118.167 | 30.108 | 828 | 9.3 | 6.6 | 12.7 | 73.1 | 52.2 | 2365.0 | 1933.0 |
|  |  |  |  |  |  | 19.4 | 17.3 | 23.0 | 85.0 | 67.8 | 187.7 | 167.9 |
| 91 | Huangshan, Anhui Province | *P. mume* ‘Huangshan 6’ | 118.167 | 30.108 | 835 | 9.3 | 6.6 | 12.7 | 73.1 | 52.2 | 2365.0 | 1933.0 |
|  |  |  |  |  |  | 19.4 | 17.3 | 23.0 | 85.0 | 67.8 | 187.7 | 167.9 |
| 92 | Huangshan, Anhui Province | *P. mume* ‘Huangshan 7’ | 118.151 | 30.077 | 465 | 9.3 | 6.6 | 12.7 | 73.1 | 52.2 | 2365.0 | 1933.0 |
|  |  |  |  |  |  | 19.4 | 17.3 | 23.0 | 85.0 | 67.8 | 187.7 | 167.9 |
| 93 | Huangshan, Anhui Province | *P. mume* ‘Huangshan 8’ | 118.155 | 30.079 | 649 | 9.3 | 6.6 | 12.7 | 73.1 | 52.2 | 2365.0 | 1933.0 |
|  |  |  |  |  |  | 19.4 | 17.3 | 23.0 | 85.0 | 67.8 | 187.7 | 167.9 |
| 94 | Huangshan, Anhui Province | *P. mume* ‘Huangshan 9’ | 118.155 | 30.079 | 637 | 9.3 | 6.6 | 12.7 | 73.1 | 52.2 | 2365.0 | 1933.0 |
|  |  |  |  |  |  | 19.4 | 17.3 | 23.0 | 85.0 | 67.8 | 187.7 | 167.9 |
| 95 | Jingdezhen, Jiangxi Province | *P. mume* ‘Jingdezhen 1’ | 117.131 | 29.484 | 77 | 18.9 | 14.9 | 24.4 | 68.5 | 44.6 | 1505.5 | 1908.7 |
|  |  |  |  |  |  | 31.7 | 27.5 | 36.7 | 59.0 | 38.7 | 28.1 | 256.3 |
| 96 | Jingdezhen, Jiangxi Province | *P. mume* ‘Jingdezhen 2’ | 117.129 | 29.491 | 86 | 18.9 | 14.9 | 24.4 | 68.5 | 44.6 | 1505.5 | 1908.7 |
|  |  |  |  |  |  | 31.7 | 27.5 | 36.7 | 59.0 | 38.7 | 28.1 | 256.3 |

^a^No. stands for serial number. No. 1 − No. 59 were cultivars, No. 60 − No. 82 were wild types from southwest China, and No.83 − No. 96 were wild types from southeast China, respectively.

^b^The meteorological data include annual (upper; September 1st, 2012–August 31st, 2013) and monthly profiles (lower; August 1st, 2013–August 31st, 2013).

**Supplementary TABLE 2 | Bands generated by 15 primer-pair combinations for AFLP selective amplification^a^**.

| **Primer-pair combinations** | **Number of markers** | **Range of fragment length (bp)** | **Number of polymorphic markers** |
| --- | --- | --- | --- |
| E(AAC)–M(CTG) | 115 | 59–612 | 91 |
| E(AAG)–M(CTA) | 209 | 64–746 | 183 |
| E(ACA)–M(CAA) | 192 | 66–732 | 163 |
| E(ACA)–M(CAT) | 181 | 59–759 | 154 |
| E(ACC)–M(CAC) | 102 | 63–543 | 73 |
| E(ACC)–M(CAG) | 112 | 54–600 | 84 |
| E(ACG)–M(CTA) | 168 | 63–706 | 145 |
| E(ACG)–M(CTT) | 212 | 61–764 | 190 |
| E(ACT)–M(CAC) | 120 | 77–766 | 97 |
| E(ACT)–M(CAT) | 118 | 59–707 | 94 |
| E(AGC)–M(CTC) | 181 | 70–713 | 160 |
| E(AGC)–M(CTG) | 112 | 70–674 | 87 |
| E(AGG)–M(CAA) | 169 | 66–740 | 143 |
| E(AGG)–M(CAG) | 132 | 60–642 | 96 |
| E(AGG)–M(CTC) | 131 | 58–654 | 104 |
| Total | 2254 | 54–766 | 1864 |

^a^The primers of E(×××) were modified with 5'-FAM.

**Supplementary TABLE 3 | Bands generated by 15 primer-pair combinations for MSAP selective amplification^a^.**

^a^The primers of E(×××) were modified with 5'-FAM.

| **Primer-pair combinations** | **Number of markers** | **Range of fragment length (bp)** | **Number of polymorphic markers** |
| --- | --- | --- | --- |
| E(AAC)–H/M(TCG) | 157 | 61–712 | 154 |
| E(AAC)–H/M(TTC) | 196 | 61–586 | 193 |
| E(AAG)–H/M(TTG) | 221 | 65–618 | 220 |
| E(AAG)–H/M(TTA) | 146 | 62–614 | 143 |
| E(ACA)–H/M(TCG) | 214 | 61–628 | 210 |
| E(ACA)–H/M(TTG) | 214 | 61–573 | 211 |
| E(ACC)–H/M(TCC) | 182 | 59–597 | 179 |
| E(ACC)–H/M(TTC) | 225 | 61–703 | 223 |
| E(ACG)–H/M(TCT) | 179 | 60–585 | 176 |
| E(ACG)–H/M(TGT) | 213 | 61–601 | 212 |
| E(ACT)–H/M(TTC) | 171 | 63–629 | 170 |
| E(ACT)–H/M(TTA) | 179 | 61–548 | 174 |
| E(AGC)–H/M(TCT) | 187 | 62–607 | 185 |
| E(AGC)–H/M(TCC) | 196 | 64–607 | 194 |
| E(AGG)–H/M(TGA) | 169 | 63–556 | 166 |
| Total | 2849 | 59–712 | 2810 |

**Supplementary TABLE 4 | AFLP markers associated with leaf shape traits in populations of *Prunus* *mume***.

| **Number^a^** | **AFLP marker^b^** | **Traits^c^** | | | | **Variation explained (%)** | ***P*-value** |
| --- | --- | --- | --- | --- | --- | --- | --- |
| 74 | E(AAC)-M(CTG)-371 |  |  | Ratio |  | 3.46 | 0.021 |
| 92 | E(AAG)-M(CTA)-064 | Leaf length |  |  |  | 3.62 | 0.004 |
|  | E(AAG)-M(CTA)-064 |  | Leaf width |  |  | 3.97 | 0.003 |
|  | E(AAG)-M(CTA)-064 |  |  |  | Leaf area | 3.35 | 0.006 |
| 94 | E(AAG)-M(CTA)-067 | Leaf length |  |  |  | 3.83 | 0.003 |
|  | E(AAG)-M(CTA)-067 |  | Leaf width |  |  | 4.12 | 0.003 |
|  | E(AAG)-M(CTA)-067 |  |  |  | Leaf area | 3.35 | 0.006 |
| 95 | E(AAG)-M(CTA)-069 | Leaf length |  |  |  | 4.76 | 0.001 |
|  | E(AAG)-M(CTA)-069 |  | Leaf width |  |  | 4.14 | 0.003 |
|  | E(AAG)-M(CTA)-069 |  |  |  | Leaf area | 4.22 | 0.002 |
| 99 | E(AAG)-M(CTA)-078 | Leaf length |  |  |  | 3.08 | 0.007 |
|  | E(AAG)-M(CTA)-078 |  | Leaf width |  |  | 2.77 | 0.014 |
|  | E(AAG)-M(CTA)-078 |  |  |  | Leaf area | 3.09 | 0.008 |
| 100 | E(AAG)-M(CTA)-080 | Leaf length |  |  |  | 2.89 | 0.009 |
|  | E(AAG)-M(CTA)-080 |  | Leaf width |  |  | 2.46 | 0.021 |
|  | E(AAG)-M(CTA)-080 |  |  |  | Leaf area | 2.89 | 0.010 |
| 103 | E(AAG)-M(CTA)-088 | Leaf length |  |  |  | 2.94 | 0.009 |
|  | E(AAG)-M(CTA)-088 |  | Leaf width |  |  | 3.29 | 0.008 |
|  | E(AAG)-M(CTA)-088 |  |  |  | Leaf area | 3.25 | 0.006 |
| 108 | E(AAG)-M(CTA)-098 | Leaf length |  |  |  | 3.62 | 0.004 |
|  | E(AAG)-M(CTA)-098 |  | Leaf width |  |  | 3.97 | 0.003 |
|  | E(AAG)-M(CTA)-098 |  |  |  | Leaf area | 3.35 | 0.006 |
| 116 | E(AAG)-M(CTA)-117 | Leaf length |  |  |  | 2.69 | 0.012 |
|  | E(AAG)-M(CTA)-117 |  |  | Ratio |  | 2.60 | 0.046 |
| 125 | E(AAG)-M(CTA)-144 | Leaf length |  |  |  | 2.34 | 0.020 |
|  | E(AAG)-M(CTA)-144 |  | Leaf width |  |  | 2.79 | 0.014 |
|  | E(AAG)-M(CTA)-144 |  |  |  | Leaf area | 2.21 | 0.026 |
| 126 | E(AAG)-M(CTA)-148 | Leaf length |  |  |  | 3.79 | 0.003 |
|  | E(AAG)-M(CTA)-148 |  | Leaf width |  |  | 2.39 | 0.024 |
|  | E(AAG)-M(CTA)-148 |  |  | Ratio |  | 3.40 | 0.022 |
|  | E(AAG)-M(CTA)-148 |  |  |  | Leaf area | 3.08 | 0.008 |
| 127 | E(AAG)-M(CTA)-152 | Leaf length |  |  |  | 2.90 | 0.009 |
|  | E(AAG)-M(CTA)-152 |  | Leaf width |  |  | 3.88 | 0.004 |
|  | E(AAG)-M(CTA)-152 |  |  |  | Leaf area | 2.73 | 0.013 |
| 131 | E(AAG)-M(CTA)-168 | Leaf length |  |  |  | 3.64 | 0.003 |
|  | E(AAG)-M(CTA)-168 |  | Leaf width |  |  | 3.81 | 0.004 |
|  | E(AAG)-M(CTA)-168 |  |  |  | Leaf area | 3.29 | 0.006 |
| 140 | E(AAG)-M(CTA)-189 | Leaf length |  |  |  | 3.86 | 0.003 |
|  | E(AAG)-M(CTA)-189 |  | Leaf width |  |  | 3.13 | 0.009 |
|  | E(AAG)-M(CTA)-189 |  |  |  | Leaf area | 3.54 | 0.004 |
| 151 | E(AAG)-M(CTA)-218 | Leaf length |  |  |  | 2.71 | 0.012 |
|  | E(AAG)-M(CTA)-218 |  | Leaf width |  |  | 2.50 | 0.020 |
|  | E(AAG)-M(CTA)-218 |  |  |  | Leaf area | 2.25 | 0.024 |
| 156 | E(AAG)-M(CTA)-227 | Leaf length |  |  |  | 2.92 | 0.009 |
|  | E(AAG)-M(CTA)-227 |  | Leaf width |  |  | 2.83 | 0.013 |
|  | E(AAG)-M(CTA)-227 |  |  |  | Leaf area | 2.82 | 0.011 |
| 161 | E(AAG)-M(CTA)-236 | Leaf length |  |  |  | 2.43 | 0.018 |
|  | E(AAG)-M(CTA)-236 |  | Leaf width |  |  | 3.00 | 0.011 |
|  | E(AAG)-M(CTA)-236 |  |  |  | Leaf area | 2.57 | 0.016 |
| 174 | E(AAG)-M(CTA)-260 | Leaf length |  |  |  | 2.62 | 0.014 |
|  | E(AAG)-M(CTA)-260 |  | Leaf width |  |  | 3.39 | 0.007 |
|  | E(AAG)-M(CTA)-260 |  |  |  | Leaf area | 2.78 | 0.012 |
| 177 | E(AAG)-M(CTA)-265 | Leaf length |  |  |  | 2.61 | 0.014 |
|  | E(AAG)-M(CTA)-265 |  |  |  | Leaf area | 2.01 | 0.033 |
| 180 | E(AAG)-M(CTA)-270 | Leaf length |  |  |  | 1.97 | 0.033 |
| 182 | E(AAG)-M(CTA)-273 |  | Leaf width |  |  | 2.04 | 0.037 |
| 185 | E(AAG)-M(CTA)-281 |  | Leaf width |  |  | 2.03 | 0.037 |
| 189 | E(AAG)-M(CTA)-293 | Leaf length |  |  |  | 1.76 | 0.044 |
| 192 | E(AAG)-M(CTA)-305 |  | Leaf width |  |  | 2.51 | 0.020 |
|  | E(AAG)-M(CTA)-305 |  |  |  | Leaf area | 1.72 | 0.049 |
| 201 | E(AAG)-M(CTA)-330 |  |  |  | Leaf area | 2.25 | 0.024 |
| 205 | E(AAG)-M(CTA)-347 | Leaf length |  |  |  | 1.91 | 0.036 |
| 211 | E(AAG)-M(CTA)-362 |  | Leaf width |  |  | 1.94 | 0.042 |
| 213 | E(AAG)-M(CTA)-367 |  | Leaf width |  |  | 1.92 | 0.043 |
|  | E(AAG)-M(CTA)-367 |  |  |  | Leaf area | 1.94 | 0.037 |
| 218 | E(AAG)-M(CTA)-390 | Leaf length |  |  |  | 1.92 | 0.035 |
|  | E(AAG)-M(CTA)-390 |  |  |  | Leaf area | 1.85 | 0.042 |
| 221 | E(AAG)-M(CTA)-403 | Leaf length |  |  |  | 2.18 | 0.025 |
|  | E(AAG)-M(CTA)-403 |  | Leaf width |  |  | 1.87 | 0.046 |
| 228 | E(AAG)-M(CTA)-426 |  | Leaf width |  |  | 2.00 | 0.038 |
|  | E(AAG)-M(CTA)-426 |  |  |  | Leaf area | 1.79 | 0.045 |
| 237 | E(AAG)-M(CTA)-455 | Leaf length |  |  |  | 4.14 | 0.002 |
|  | E(AAG)-M(CTA)-455 |  | Leaf width |  |  | 4.79 | 0.001 |
|  | E(AAG)-M(CTA)-455 |  |  |  | Leaf area | 4.38 | 0.001 |
| 241 | E(AAG)-M(CTA)-464 | Leaf length |  |  |  | 2.76 | 0.011 |
|  | E(AAG)-M(CTA)-464 |  | Leaf width |  |  | 2.75 | 0.015 |
|  | E(AAG)-M(CTA)-464 |  |  |  | Leaf area | 2.92 | 0.010 |
| 242 | E(AAG)-M(CTA)-465 | Leaf length |  |  |  | 6.60 | 0.000 |
|  | E(AAG)-M(CTA)-465 |  | Leaf width |  |  | 7.00 | 0.000 |
|  | E(AAG)-M(CTA)-465 |  |  |  | Leaf area | 6.42 | 0.000 |
| 245 | E(AAG)-M(CTA)-474 |  | Leaf width |  |  | 2.20 | 0.030 |
| 248 | E(AAG)-M(CTA)-487 | Leaf length |  |  |  | 2.06 | 0.029 |
|  | E(AAG)-M(CTA)-487 |  | Leaf width |  |  | 2.75 | 0.015 |
|  | E(AAG)-M(CTA)-487 |  |  |  | Leaf area | 1.84 | 0.042 |
| 251 | E(AAG)-M(CTA)-496 | Leaf length |  |  |  | 1.93 | 0.035 |
|  | E(AAG)-M(CTA)-496 |  | Leaf width |  |  | 1.88 | 0.045 |
| 252 | E(AAG)-M(CTA)-498 | Leaf length |  |  |  | 2.65 | 0.013 |
|  | E(AAG)-M(CTA)-498 |  | Leaf width |  |  | 3.25 | 0.008 |
|  | E(AAG)-M(CTA)-498 |  |  |  | Leaf area | 2.47 | 0.018 |
| 253 | E(AAG)-M(CTA)-503 | Leaf length |  |  |  | 2.79 | 0.011 |
|  | E(AAG)-M(CTA)-503 |  | Leaf width |  |  | 3.72 | 0.004 |
|  | E(AAG)-M(CTA)-503 |  |  |  | Leaf area | 2.91 | 0.010 |
| 256 | E(AAG)-M(CTA)-512 | Leaf length |  |  |  | 2.85 | 0.010 |
|  | E(AAG)-M(CTA)-512 |  | Leaf width |  |  | 4.24 | 0.002 |
|  | E(AAG)-M(CTA)-512 |  |  |  | Leaf area | 3.29 | 0.006 |
| 257 | E(AAG)-M(CTA)-525 | Leaf length |  |  |  | 2.64 | 0.013 |
|  | E(AAG)-M(CTA)-525 |  | Leaf width |  |  | 3.65 | 0.005 |
|  | E(AAG)-M(CTA)-525 |  |  |  | Leaf area | 3.03 | 0.009 |
| 258 | E(AAG)-M(CTA)-527 | Leaf length |  |  |  | 2.57 | 0.015 |
|  | E(AAG)-M(CTA)-527 |  | Leaf width |  |  | 4.71 | 0.001 |
|  | E(AAG)-M(CTA)-527 |  |  |  | Leaf area | 3.41 | 0.005 |
| 260 | E(AAG)-M(CTA)-530 |  | Leaf width |  |  | 2.41 | 0.023 |
| 263 | E(AAG)-M(CTA)-544 |  | Leaf width |  |  | 1.89 | 0.045 |
| 264 | E(AAG)-M(CTA)-546 |  |  | Ratio |  | 3.77 | 0.016 |
| 266 | E(AAG)-M(CTA)-559 | Leaf length |  |  |  | 2.53 | 0.015 |
|  | E(AAG)-M(CTA)-559 |  | Leaf width |  |  | 2.57 | 0.019 |
|  | E(AAG)-M(CTA)-559 |  |  |  | Leaf area | 2.25 | 0.024 |
| 267 | E(AAG)-M(CTA)-560 | Leaf length |  |  |  | 2.12 | 0.027 |
|  | E(AAG)-M(CTA)-560 |  | Leaf width |  |  | 2.73 | 0.015 |
|  | E(AAG)-M(CTA)-560 |  |  |  | Leaf area | 1.85 | 0.042 |
| 300 | E(ACA)-M(CAA)-120 |  |  | Ratio |  | 3.05 | 0.030 |
| 305 | E(ACA)-M(CAA)-133 | Leaf length |  |  |  | 2.02 | 0.031 |
|  | E(ACA)-M(CAA)-133 |  |  |  | Leaf area | 1.95 | 0.036 |
| 317 | E(ACA)-M(CAA)-166 | Leaf length |  |  |  | 1.80 | 0.042 |
|  | E(ACA)-M(CAA)-166 |  | Leaf width |  |  | 3.10 | 0.010 |
|  | E(ACA)-M(CAA)-166 |  |  |  | Leaf area | 2.89 | 0.010 |
| 327 | E(ACA)-M(CAA)-186 | Leaf length |  |  |  | 2.85 | 0.010 |
|  | E(ACA)-M(CAA)-186 |  | Leaf width |  |  | 2.39 | 0.023 |
|  | E(ACA)-M(CAA)-186 |  |  |  | Leaf area | 3.00 | 0.009 |
| 341 | E(ACA)-M(CAA)-222 | Leaf length |  |  |  | 3.39 | 0.005 |
|  | E(ACA)-M(CAA)-222 |  | Leaf width |  |  | 2.15 | 0.032 |
|  | E(ACA)-M(CAA)-222 |  |  |  | Leaf area | 3.46 | 0.005 |
| 345 | E(ACA)-M(CAA)-230 |  |  | Ratio |  | 4.89 | 0.006 |
| 347 | E(ACA)-M(CAA)-237 |  |  | Ratio |  | 4.87 | 0.006 |
| 354 | E(ACA)-M(CAA)-257 |  | Leaf width |  |  | 2.77 | 0.015 |
|  | E(ACA)-M(CAA)-257 |  |  |  | Leaf area | 2.12 | 0.029 |
| 361 | E(ACA)-M(CAA)-269 | Leaf length |  |  |  | 2.52 | 0.016 |
|  | E(ACA)-M(CAA)-269 |  | Leaf width |  |  | 1.97 | 0.040 |
|  | E(ACA)-M(CAA)-269 |  |  |  | Leaf area | 2.39 | 0.020 |
| 363 | E(ACA)-M(CAA)-279 |  | Leaf width |  |  | 1.98 | 0.040 |
| 403 | E(ACA)-M(CAA)-444 |  | Leaf width |  |  | 1.98 | 0.040 |
| 418 | E(ACA)-M(CAA)-490 | Leaf length |  |  |  | 2.08 | 0.028 |
|  | E(ACA)-M(CAA)-490 |  | Leaf width |  |  | 2.30 | 0.026 |
| 432 | E(ACA)-M(CAA)-622 |  |  | Ratio |  | 2.72 | 0.041 |
| 435 | E(ACA)-M(CAA)-691 |  |  | Ratio |  | 3.64 | 0.017 |
| 457 | E(ACA)-M(CAT)-103 | Leaf length |  |  |  | 2.79 | 0.011 |
|  | E(ACA)-M(CAT)-103 |  | Leaf width |  |  | 2.17 | 0.031 |
|  | E(ACA)-M(CAT)-103 |  |  |  | Leaf area | 2.60 | 0.015 |
| 491 | E(ACA)-M(CAT)-191 | Leaf length |  |  |  | 2.84 | 0.010 |
|  | E(ACA)-M(CAT)-191 |  | Leaf width |  |  | 3.82 | 0.004 |
|  | E(ACA)-M(CAT)-191 |  |  |  | Leaf area | 3.48 | 0.005 |
| 505 | E(ACA)-M(CAT)-225 |  |  | Ratio |  | 3.93 | 0.013 |
| 512 | E(ACA)-M(CAT)-262 |  | Leaf width |  |  | 2.12 | 0.033 |
|  | E(ACA)-M(CAT)-262 |  |  |  | Leaf area | 1.83 | 0.043 |
| 525 | E(ACA)-M(CAT)-320 |  |  | Ratio |  | 3.49 | 0.020 |
| 543 | E(ACA)-M(CAT)-393 |  | Leaf width |  |  | 2.63 | 0.017 |
|  | E(ACA)-M(CAT)-393 |  |  |  | Leaf area | 1.96 | 0.036 |
| 548 | E(ACA)-M(CAT)-410 | Leaf length |  |  |  | 2.19 | 0.024 |
| 599 | E(ACC)-M(CAC)-087 | Leaf length |  |  |  | 1.94 | 0.034 |
|  | E(ACC)-M(CAC)-087 |  |  |  | Leaf area | 2.74 | 0.013 |
| 618 | E(ACC)-M(CAC)-147 |  |  | Ratio |  | 2.87 | 0.035 |
| 622 | E(ACC)-M(CAC)-160 | Leaf length |  |  |  | 1.99 | 0.032 |
|  | E(ACC)-M(CAC)-160 |  | Leaf width |  |  | 2.74 | 0.015 |
|  | E(ACC)-M(CAC)-160 |  |  |  | Leaf area | 2.75 | 0.012 |
| 626 | E(ACC)-M(CAC)-167 | Leaf length |  |  |  | 2.74 | 0.012 |
|  | E(ACC)-M(CAC)-167 |  |  |  | Leaf area | 1.98 | 0.035 |
| 659 | E(ACC)-M(CAC)-334 | Leaf length |  |  |  | 2.05 | 0.030 |
|  | E(ACC)-M(CAC)-334 |  | Leaf width |  |  | 2.38 | 0.024 |
|  | E(ACC)-M(CAC)-334 |  |  |  | Leaf area | 2.34 | 0.022 |
| 660 | E(ACC)-M(CAC)-357 | Leaf length |  |  |  | 1.98 | 0.033 |
|  | E(ACC)-M(CAC)-357 |  |  |  | Leaf area | 1.82 | 0.043 |
| 683 | E(ACC)-M(CAG)-110 | Leaf length |  |  |  | 2.52 | 0.016 |
|  | E(ACC)-M(CAG)-110 |  |  |  | Leaf area | 1.72 | 0.049 |
| 688 | E(ACC)-M(CAG)-129 |  | Leaf width |  |  | 2.22 | 0.029 |
|  | E(ACC)-M(CAG)-129 |  |  |  | Leaf area | 1.90 | 0.039 |
| 690 | E(ACC)-M(CAG)-135 |  |  | Ratio |  | 2.58 | 0.047 |
| 694 | E(ACC)-M(CAG)-146 |  |  | Ratio |  | 2.91 | 0.034 |
| 695 | E(ACC)-M(CAG)-147 | Leaf length |  |  |  | 2.25 | 0.023 |
| 716 | E(ACC)-M(CAG)-238 |  |  | Ratio |  | 3.95 | 0.013 |
| 719 | E(ACC)-M(CAG)-254 |  |  | Ratio |  | 5.28 | 0.004 |
| 722 | E(ACC)-M(CAG)-292 |  | Leaf width |  |  | 2.95 | 0.011 |
|  | E(ACC)-M(CAG)-292 |  |  | Ratio |  | 3.40 | 0.022 |
| 728 | E(ACC)-M(CAG)-329 | Leaf length |  |  |  | 2.25 | 0.022 |
|  | E(ACC)-M(CAG)-329 |  |  | Ratio |  | 4.58 | 0.007 |
| 738 | E(ACC)-M(CAG)-406 |  |  | Ratio |  | 2.93 | 0.033 |
| 755 | E(ACG)-M(CTA)-073 |  |  | Ratio |  | 3.59 | 0.018 |
| 764 | E(ACG)-M(CTA)-093 | Leaf length |  |  |  | 1.70 | 0.048 |
|  | E(ACG)-M(CTA)-093 |  |  |  | Leaf area | 1.75 | 0.048 |
| 773 | E(ACG)-M(CTA)-108 |  | Leaf width |  |  | 3.33 | 0.007 |
|  | E(ACG)-M(CTA)-108 |  |  |  | Leaf area | 2.31 | 0.022 |
| 776 | E(ACG)-M(CTA)-114 |  |  | Ratio |  | 4.94 | 0.005 |
| 788 | E(ACG)-M(CTA)-144 | Leaf length |  |  |  | 2.63 | 0.013 |
|  | E(ACG)-M(CTA)-144 |  | Leaf width |  |  | 2.12 | 0.033 |
|  | E(ACG)-M(CTA)-144 |  |  |  | Leaf area | 2.08 | 0.031 |
| 795 | E(ACG)-M(CTA)-160 |  | Leaf width |  |  | 1.82 | 0.048 |
| 816 | E(ACG)-M(CTA)-217 |  |  | Ratio |  | 3.29 | 0.024 |
| 832 | E(ACG)-M(CTA)-265 |  |  | Ratio |  | 4.82 | 0.006 |
| 844 | E(ACG)-M(CTA)-309 |  |  | Ratio |  | 2.89 | 0.035 |
| 845 | E(ACG)-M(CTA)-309 | Leaf length |  |  |  | 2.08 | 0.028 |
|  | E(ACG)-M(CTA)-309 |  | Leaf width |  |  | 2.29 | 0.027 |
|  | E(ACG)-M(CTA)-309 |  |  |  | Leaf area | 2.23 | 0.025 |
| 847 | E(ACG)-M(CTA)-319 |  |  | Ratio |  | 4.27 | 0.010 |
| 852 | E(ACG)-M(CTA)-343 |  | Leaf width |  |  | 1.97 | 0.040 |
|  | E(ACG)-M(CTA)-343 |  |  |  | Leaf area | 1.81 | 0.044 |
| 853 | E(ACG)-M(CTA)-349 |  |  | Ratio |  | 3.48 | 0.020 |
| 860 | E(ACG)-M(CTA)-391 |  |  | Ratio |  | 3.11 | 0.028 |
| 876 | E(ACG)-M(CTA)-475 |  | Leaf width |  |  | 2.81 | 0.014 |
|  | E(ACG)-M(CTA)-475 |  |  |  | Leaf area | 2.58 | 0.016 |
| 884 | E(ACG)-M(CTA)-534 | Leaf length |  |  |  | 2.32 | 0.020 |
|  | E(ACG)-M(CTA)-534 |  | Leaf width |  |  | 3.04 | 0.010 |
|  | E(ACG)-M(CTA)-534 |  |  |  | Leaf area | 2.98 | 0.009 |
| 885 | E(ACG)-M(CTA)-538 |  | Leaf width |  |  | 2.17 | 0.031 |
|  | E(ACG)-M(CTA)-538 |  |  |  | Leaf area | 1.97 | 0.035 |
| 886 | E(ACG)-M(CTA)-539 | Leaf length |  |  |  | 1.73 | 0.046 |
|  | E(ACG)-M(CTA)-539 |  | Leaf width |  |  | 2.09 | 0.034 |
|  | E(ACG)-M(CTA)-539 |  |  |  | Leaf area | 2.04 | 0.032 |
| 887 | E(ACG)-M(CTA)-541 | Leaf length |  |  |  | 2.53 | 0.015 |
|  | E(ACG)-M(CTA)-541 |  | Leaf width |  |  | 3.58 | 0.005 |
|  | E(ACG)-M(CTA)-541 |  |  |  | Leaf area | 3.15 | 0.007 |
| 902 | E(ACG)-M(CTT)-075 | Leaf length |  |  |  | 2.41 | 0.018 |
|  | E(ACG)-M(CTT)-075 |  | Leaf width |  |  | 2.03 | 0.037 |
|  | E(ACG)-M(CTT)-075 |  |  |  | Leaf area | 2.32 | 0.022 |
| 905 | E(ACG)-M(CTT)-083 |  | Leaf width |  |  | 2.51 | 0.020 |
|  | E(ACG)-M(CTT)-083 |  |  |  | Leaf area | 1.90 | 0.039 |
| 909 | E(ACG)-M(CTT)-090 |  | Leaf width |  |  | 2.47 | 0.021 |
| 916 | E(ACG)-M(CTT)-104 |  | Leaf width |  |  | 1.84 | 0.047 |
| 917 | E(ACG)-M(CTT)-107 | Leaf length |  |  |  | 1.73 | 0.046 |
|  | E(ACG)-M(CTT)-107 |  | Leaf width |  |  | 3.84 | 0.004 |
|  | E(ACG)-M(CTT)-107 |  |  |  | Leaf area | 2.35 | 0.021 |
| 927 | E(ACG)-M(CTT)-128 |  |  | Ratio |  | 5.28 | 0.004 |
| 928 | E(ACG)-M(CTT)-130 | Leaf length |  |  |  | 2.73 | 0.012 |
|  | E(ACG)-M(CTT)-130 |  |  |  | Leaf area | 2.04 | 0.032 |
| 934 | E(ACG)-M(CTT)-144 |  | Leaf width |  |  | 2.21 | 0.029 |
| 938 | E(ACG)-M(CTT)-161 |  | Leaf width |  |  | 1.87 | 0.046 |
| 939 | E(ACG)-M(CTT)-167 | Leaf length |  |  |  | 2.21 | 0.024 |
|  | E(ACG)-M(CTT)-167 |  | Leaf width |  |  | 2.08 | 0.035 |
|  | E(ACG)-M(CTT)-167 |  |  |  | Leaf area | 2.24 | 0.024 |
| 943 | E(ACG)-M(CTT)-175 | Leaf length |  |  |  | 3.41 | 0.005 |
|  | E(ACG)-M(CTT)-175 |  | Leaf width |  |  | 4.90 | 0.001 |
|  | E(ACG)-M(CTT)-175 |  |  |  | Leaf area | 4.11 | 0.002 |
| 944 | E(ACG)-M(CTT)-177 |  | Leaf width |  |  | 2.08 | 0.035 |
| 946 | E(ACG)-M(CTT)-183 | Leaf length |  |  |  | 4.02 | 0.002 |
|  | E(ACG)-M(CTT)-183 |  | Leaf width |  |  | 3.75 | 0.004 |
|  | E(ACG)-M(CTT)-183 |  |  |  | Leaf area | 3.73 | 0.003 |
| 949 | E(ACG)-M(CTT)-189 | Leaf length |  |  |  | 2.97 | 0.008 |
|  | E(ACG)-M(CTT)-189 |  |  | Ratio |  | 2.65 | 0.043 |
|  | E(ACG)-M(CTT)-189 |  |  |  | Leaf area | 1.92 | 0.038 |
| 950 | E(ACG)-M(CTT)-190 | Leaf length |  |  |  | 3.21 | 0.006 |
|  | E(ACG)-M(CTT)-190 |  | Leaf width |  |  | 3.64 | 0.005 |
|  | E(ACG)-M(CTT)-190 |  |  |  | Leaf area | 3.17 | 0.007 |
| 960 | E(ACG)-M(CTT)-221 | Leaf length |  |  |  | 3.71 | 0.003 |
|  | E(ACG)-M(CTT)-221 |  | Leaf width |  |  | 4.52 | 0.002 |
|  | E(ACG)-M(CTT)-221 |  |  |  | Leaf area | 4.35 | 0.002 |
| 966 | E(ACG)-M(CTT)-236 |  |  | Ratio |  | 2.89 | 0.035 |
| 968 | E(ACG)-M(CTT)-239 |  | Leaf width |  |  | 2.15 | 0.032 |
| 974 | E(ACG)-M(CTT)-256 |  |  | Ratio |  | 5.12 | 0.005 |
| 985 | E(ACG)-M(CTT)-291 | Leaf length |  |  |  | 1.83 | 0.040 |
|  | E(ACG)-M(CTT)-291 |  | Leaf width |  |  | 1.92 | 0.043 |
| 993 | E(ACG)-M(CTT)-309 | Leaf length |  |  |  | 2.71 | 0.012 |
|  | E(ACG)-M(CTT)-309 |  | Leaf width |  |  | 3.42 | 0.006 |
|  | E(ACG)-M(CTT)-309 |  |  |  | Leaf area | 2.86 | 0.011 |
| 996 | E(ACG)-M(CTT)-316 |  | Leaf width |  |  | 1.95 | 0.041 |
| 1002 | E(ACG)-M(CTT)-330 |  | Leaf width |  |  | 2.10 | 0.034 |
| 1005 | E(ACG)-M(CTT)-344 | Leaf length |  |  |  | 2.12 | 0.027 |
|  | E(ACG)-M(CTT)-344 |  | Leaf width |  |  | 1.87 | 0.046 |
|  | E(ACG)-M(CTT)-344 |  |  |  | Leaf area | 1.77 | 0.046 |
| 1015 | E(ACG)-M(CTT)-381 |  |  | Ratio |  | 2.56 | 0.047 |
| 1017 | E(ACG)-M(CTT)-392 | Leaf length |  |  |  | 2.05 | 0.030 |
|  | E(ACG)-M(CTT)-392 |  |  |  | Leaf area | 1.84 | 0.042 |
| 1043 | E(ACG)-M(CTT)-491 |  |  | Ratio |  | 2.82 | 0.037 |
| 1055 | E(ACG)-M(CTT)-533 | Leaf length |  |  |  | 1.87 | 0.038 |
| 1058 | E(ACG)-M(CTT)-547 | Leaf length |  |  |  | 2.39 | 0.018 |
| 1093 | E(ACT)-M(CAC)-095 | Leaf length |  |  |  | 1.92 | 0.035 |
|  | E(ACT)-M(CAC)-095 |  | Leaf width |  |  | 3.30 | 0.007 |
|  | E(ACT)-M(CAC)-095 |  |  |  | Leaf area | 2.40 | 0.020 |
| 1098 | E(ACT)-M(CAC)-105 | Leaf length |  |  |  | 3.96 | 0.002 |
|  | E(ACT)-M(CAC)-105 |  | Leaf width |  |  | 4.23 | 0.002 |
|  | E(ACT)-M(CAC)-105 |  |  |  | Leaf area | 4.82 | 0.001 |
| 1109 | E(ACT)-M(CAC)-136 |  |  | Ratio |  | 3.14 | 0.028 |
| 1112 | E(ACT)-M(CAC)-142 | Leaf length |  |  |  | 2.98 | 0.008 |
|  | E(ACT)-M(CAC)-142 |  | Leaf width |  |  | 3.59 | 0.005 |
|  | E(ACT)-M(CAC)-142 |  |  |  | Leaf area | 4.60 | 0.001 |
| 1113 | E(ACT)-M(CAC)-144 |  | Leaf width |  |  | 2.46 | 0.021 |
|  | E(ACT)-M(CAC)-144 |  |  |  | Leaf area | 1.79 | 0.045 |
| 1118 | E(ACT)-M(CAC)-162 |  |  | Ratio |  | 2.70 | 0.042 |
| 1125 | E(ACT)-M(CAC)-191 | Leaf length |  |  |  | 1.86 | 0.039 |
|  | E(ACT)-M(CAC)-191 |  | Leaf width |  |  | 3.00 | 0.011 |
|  | E(ACT)-M(CAC)-191 |  |  |  | Leaf area | 2.34 | 0.021 |
| 1128 | E(ACT)-M(CAC)-208 |  | Leaf width |  |  | 2.30 | 0.026 |
| 1144 | E(ACT)-M(CAC)-291 | Leaf length |  |  |  | 1.81 | 0.041 |
|  | E(ACT)-M(CAC)-291 |  |  | Ratio |  | 5.35 | 0.004 |
| 1170 | E(ACT)-M(CAC)-515 |  | Leaf width |  |  | 2.18 | 0.030 |
| 1192 | E(ACT)-M(CAT)-082 | Leaf length |  |  |  | 2.89 | 0.009 |
|  | E(ACT)-M(CAT)-082 |  |  |  | Leaf area | 2.18 | 0.027 |
| 1193 | E(ACT)-M(CAT)-084 |  |  | Ratio |  | 2.66 | 0.043 |
| 1196 | E(ACT)-M(CAT)-090 | Leaf length |  |  |  | 1.70 | 0.048 |
| 1206 | E(ACT)-M(CAT)-114 |  |  | Ratio |  | 3.04 | 0.030 |
| 1224 | E(ACT)-M(CAT)-158 | Leaf length |  |  |  | 4.13 | 0.002 |
|  | E(ACT)-M(CAT)-158 |  |  | Ratio |  | 8.73 | 0.000 |
|  | E(ACT)-M(CAT)-158 |  |  |  | Leaf area | 2.15 | 0.028 |
| 1258 | E(ACT)-M(CAT)-320 |  |  | Ratio |  | 2.56 | 0.047 |
| 1259 | E(ACT)-M(CAT)-341 |  |  | Ratio |  | 4.33 | 0.009 |
| 1282 | E(AGC)-M(CTC)-089 | Leaf length |  |  |  | 1.76 | 0.044 |
|  | E(AGC)-M(CTC)-089 |  |  |  | Leaf area | 2.16 | 0.027 |
| 1287 | E(AGC)-M(CTC)-098 | Leaf length |  |  |  | 2.24 | 0.023 |
|  | E(AGC)-M(CTC)-098 |  |  |  | Leaf area | 2.13 | 0.028 |
| 1306 | E(AGC)-M(CTC)-134 | Leaf length |  |  |  | 1.69 | 0.049 |
|  | E(AGC)-M(CTC)-134 |  | Leaf width |  |  | 1.86 | 0.046 |
|  | E(AGC)-M(CTC)-134 |  |  |  | Leaf area | 2.22 | 0.025 |
| 1308 | E(AGC)-M(CTC)-137 |  |  | Ratio |  | 3.65 | 0.017 |
| 1309 | E(AGC)-M(CTC)-139 |  | Leaf width |  |  | 2.65 | 0.017 |
|  | E(AGC)-M(CTC)-139 |  |  | Ratio |  | 3.64 | 0.017 |
|  | E(AGC)-M(CTC)-139 |  |  |  | Leaf area | 1.73 | 0.049 |
| 1318 | E(AGC)-M(CTC)-162 | Leaf length |  |  |  | 1.90 | 0.036 |
|  | E(AGC)-M(CTC)-162 |  |  |  | Leaf area | 1.84 | 0.042 |
| 1325 | E(AGC)-M(CTC)-184 | Leaf length |  |  |  | 1.82 | 0.041 |
|  | E(AGC)-M(CTC)-184 |  | Leaf width |  |  | 2.91 | 0.012 |
|  | E(AGC)-M(CTC)-184 |  |  |  | Leaf area | 2.09 | 0.030 |
| 1338 | E(AGC)-M(CTC)-218 | Leaf length |  |  |  | 2.09 | 0.028 |
|  | E(AGC)-M(CTC)-218 |  |  | Ratio |  | 4.47 | 0.008 |
| 1341 | E(AGC)-M(CTC)-226 |  | Leaf width |  |  | 1.92 | 0.043 |
| 1342 | E(AGC)-M(CTC)-230 |  |  |  | Leaf area | 2.00 | 0.034 |
| 1343 | E(AGC)-M(CTC)-235 |  |  |  | Leaf area | 1.73 | 0.049 |
| 1355 | E(AGC)-M(CTC)-267 | Leaf length |  |  |  | 1.70 | 0.048 |
|  | E(AGC)-M(CTC)-267 |  |  |  | Leaf area | 2.00 | 0.034 |
| 1357 | E(AGC)-M(CTC)-270 | Leaf length |  |  |  | 1.97 | 0.033 |
| 1376 | E(AGC)-M(CTC)-320 |  |  | Ratio |  | 3.04 | 0.030 |
| 1392 | E(AGC)-M(CTC)-426 | Leaf length |  |  |  | 1.94 | 0.034 |
| 1404 | E(AGC)-M(CTC)-462 |  |  | Ratio |  | 5.10 | 0.005 |
| 1408 | E(AGC)-M(CTC)-475 | Leaf length |  |  |  | 2.31 | 0.021 |
|  | E(AGC)-M(CTC)-475 |  |  | Ratio |  | 5.77 | 0.003 |
| 1412 | E(AGC)-M(CTC)-484 | Leaf length |  |  |  | 2.71 | 0.012 |
|  | E(AGC)-M(CTC)-484 |  |  |  | Leaf area | 2.65 | 0.014 |
| 1413 | E(AGC)-M(CTC)-519 |  |  | Ratio |  | 4.67 | 0.007 |
| 1418 | E(AGC)-M(CTC)-553 |  |  | Ratio |  | 3.28 | 0.024 |
| 1423 | E(AGC)-M(CTC)-599 | Leaf length |  |  |  | 2.02 | 0.031 |
| 1433 | E(AGC)-M(CTC)-709 | Leaf length |  |  |  | 1.86 | 0.039 |
|  | E(AGC)-M(CTC)-709 |  |  |  | Leaf area | 1.97 | 0.036 |
| 1434 | E(AGC)-M(CTC)-713 | Leaf length |  |  |  | 1.86 | 0.039 |
|  | E(AGC)-M(CTC)-713 |  |  |  | Leaf area | 1.97 | 0.036 |
| 1475 | E(AGC)-M(CTG)-216 |  | Leaf width |  |  | 1.92 | 0.043 |
|  | E(AGC)-M(CTG)-216 |  |  |  | Leaf area | 1.91 | 0.038 |
| 1538 | E(AGG)-M(CAA)-106 |  |  | Ratio |  | 4.20 | 0.011 |
| 1553 | E(AGG)-M(CAA)-135 | Leaf length |  |  |  | 2.08 | 0.028 |
|  | E(AGG)-M(CAA)-135 |  |  |  | Leaf area | 2.00 | 0.034 |
| 1555 | E(AGG)-M(CAA)-143 | Leaf length |  |  |  | 2.70 | 0.012 |
|  | E(AGG)-M(CAA)-143 |  |  |  | Leaf area | 2.73 | 0.013 |
| 1570 | E(AGG)-M(CAA)-196 |  | Leaf width |  |  | 2.20 | 0.030 |
| 1576 | E(AGG)-M(CAA)-209 | Leaf length |  |  |  | 2.26 | 0.022 |
|  | E(AGG)-M(CAA)-209 |  | Leaf width |  |  | 2.86 | 0.013 |
|  | E(AGG)-M(CAA)-209 |  |  |  | Leaf area | 3.05 | 0.008 |
| 1625 | E(AGG)-M(CAA)-418 | Leaf length |  |  |  | 3.19 | 0.006 |
|  | E(AGG)-M(CAA)-418 |  | Leaf width |  |  | 3.00 | 0.011 |
|  | E(AGG)-M(CAA)-418 |  |  |  | Leaf area | 3.23 | 0.007 |
| 1626 | E(AGG)-M(CAA)-423 | Leaf length |  |  |  | 1.76 | 0.044 |
|  | E(AGG)-M(CAA)-423 |  |  |  | Leaf area | 2.02 | 0.033 |
| 1630 | E(AGG)-M(CAA)-433 | Leaf length |  |  |  | 1.78 | 0.043 |
|  | E(AGG)-M(CAA)-433 |  |  | Ratio |  | 3.64 | 0.017 |
| 1638 | E(AGG)-M(CAA)-454 |  |  | Ratio |  | 2.64 | 0.044 |
| 1667 | E(AGG)-M(CAG)-065 | Leaf length |  |  |  | 2.09 | 0.028 |
|  | E(AGG)-M(CAG)-065 |  | Leaf width |  |  | 2.56 | 0.019 |
|  | E(AGG)-M(CAG)-065 |  |  |  | Leaf area | 2.51 | 0.017 |
| 1669 | E(AGG)-M(CAG)-068 |  | Leaf width |  |  | 1.83 | 0.048 |
|  | E(AGG)-M(CAG)-068 |  |  |  | Leaf area | 2.11 | 0.029 |
| 1675 | E(AGG)-M(CAG)-078 |  |  | Ratio |  | 2.69 | 0.042 |
| 1679 | E(AGG)-M(CAG)-092 | Leaf length |  |  |  | 1.92 | 0.035 |
| 1690 | E(AGG)-M(CAG)-124 | Leaf length |  |  |  | 1.74 | 0.046 |
|  | E(AGG)-M(CAG)-124 |  | Leaf width |  |  | 2.53 | 0.020 |
|  | E(AGG)-M(CAG)-124 |  |  |  | Leaf area | 2.24 | 0.025 |
| 1699 | E(AGG)-M(CAG)-154 | Leaf length |  |  |  | 1.81 | 0.041 |
|  | E(AGG)-M(CAG)-154 |  |  | Ratio |  | 6.14 | 0.002 |
| 1714 | E(AGG)-M(CAG)-204 |  |  |  | Leaf area | 2.12 | 0.029 |
| 1721 | E(AGG)-M(CAG)-240 | Leaf length |  |  |  | 2.42 | 0.018 |
|  | E(AGG)-M(CAG)-240 |  |  |  | Leaf area | 2.09 | 0.030 |
| 1723 | E(AGG)-M(CAG)-257 |  |  | Ratio |  | 3.25 | 0.025 |
| 1727 | E(AGG)-M(CAG)-267 | Leaf length |  |  |  | 1.71 | 0.047 |
|  | E(AGG)-M(CAG)-267 |  | Leaf width |  |  | 1.92 | 0.043 |
|  | E(AGG)-M(CAG)-267 |  |  |  | Leaf area | 2.07 | 0.031 |
| 1728 | E(AGG)-M(CAG)-276 |  |  | Ratio |  | 3.19 | 0.026 |
| 1741 | E(AGG)-M(CAG)-349 |  |  | Ratio |  | 4.51 | 0.008 |
| 1783 | E(AGG)-M(CTC)-113 |  |  | Ratio |  | 2.99 | 0.032 |
| 1793 | E(AGG)-M(CTC)-151 | Leaf length |  |  |  | 1.70 | 0.048 |
| 1799 | E(AGG)-M(CTC)-168 |  | Leaf width |  |  | 2.53 | 0.020 |
| 1802 | E(AGG)-M(CTC)-176 |  |  | Ratio |  | 3.30 | 0.024 |
| 1823 | E(AGG)-M(CTC)-267 | Leaf length |  |  |  | 2.87 | 0.010 |
|  | E(AGG)-M(CTC)-267 |  |  | Ratio |  | 3.59 | 0.018 |
|  | E(AGG)-M(CTC)-267 |  |  |  | Leaf area | 2.21 | 0.026 |
| 1827 | E(AGG)-M(CTC)-291 |  | Leaf width |  |  | 1.91 | 0.043 |
| 1834 | E(AGG)-M(CTC)-317 |  |  | Ratio |  | 2.72 | 0.041 |
| 1840 | E(AGG)-M(CTC)-366 |  | Leaf width |  |  | 1.88 | 0.045 |
| 1857 | E(AGG)-M(CTC)-487 |  |  | Ratio |  | 2.71 | 0.041 |

^a^The number was consistent with the sequential order of reading AFLP bands.

^b^The AFLP-marker was denominated as primer combination and fragment length.

^c^Ratio stood for ratio of leaf length to width.

**Supplementary TABLE 5 | MSAP markers associated with leaf shape traits in populations of *Prunus* *mume*.**

| **Number^a^** | **MSAP marker^b^** | **Traits^c^** | | | | **Variation explained**  **(%)** | ***P*-value** |
| --- | --- | --- | --- | --- | --- | --- | --- |
| 8 | E(AAC)-H/M(TCG)-075 | Leaf length |  |  |  | 6.11 | 0.002 |
|  | E(AAC)-H/M(TCG)-075 |  | Leaf width |  |  | 4.68 | 0.017 |
|  | E(AAC)-H/M(TCG)-075 |  |  |  | Leaf area | 4.84 | 0.011 |
| 14 | E(AAC)-H/M(TCG)-091 |  |  | Ratio |  | 5.93 | 0.026 |
| 15 | E(AAC)-H/M(TCG)-092 |  |  | Ratio |  | 9.13 | 0.001 |
| 18 | E(AAC)-H/M(TCG)-099 |  |  |  | Leaf area | 2.68 | 0.049 |
| 20 | E(AAC)-H/M(TCG)-103 |  |  |  | Leaf area | 3.94 | 0.030 |
| 32 | E(AAC)-H/M(TCG)-121 |  | Leaf width |  |  | 3.80 | 0.042 |
| 36 | E(AAC)-H/M(TCG)-127 |  |  | Ratio |  | 9.60 | 0.001 |
| 37 | E(AAC)-H/M(TCG)-129 |  |  | Ratio |  | 3.97 | 0.046 |
| 38 | E(AAC)-H/M(TCG)-130 | Leaf length |  |  |  | 3.57 | 0.040 |
|  | E(AAC)-H/M(TCG)-130 |  |  | Ratio |  | 7.15 | 0.010 |
| 45 | E(AAC)-H/M(TCG)-142 |  | Leaf width |  |  | 3.37 | 0.026 |
| 49 | E(AAC)-H/M(TCG)-150 | Leaf length |  |  |  | 4.96 | 0.008 |
|  | E(AAC)-H/M(TCG)-150 |  | Leaf width |  |  | 4.67 | 0.017 |
|  | E(AAC)-H/M(TCG)-150 |  |  |  | Leaf area | 5.05 | 0.009 |
| 56 | E(AAC)-H/M(TCG)-161 |  |  | Ratio |  | 5.87 | 0.027 |
| 57 | E(AAC)-H/M(TCG)-162 | Leaf length |  |  |  | 4.77 | 0.010 |
|  | E(AAC)-H/M(TCG)-162 |  | Leaf width |  |  | 4.14 | 0.029 |
|  | E(AAC)-H/M(TCG)-162 |  |  |  | Leaf area | 4.23 | 0.022 |
| 69 | E(AAC)-H/M(TCG)-185 |  | Leaf width |  |  | 3.78 | 0.042 |
| 71 | E(AAC)-H/M(TCG)-190 |  | Leaf width |  |  | 3.68 | 0.047 |
| 80 | E(AAC)-H/M(TCG)-207 |  |  | Ratio |  | 5.14 | 0.018 |
| 81 | E(AAC)-H/M(TCG)-208 | Leaf length |  |  |  | 5.16 | 0.006 |
|  | E(AAC)-H/M(TCG)-208 |  |  | Ratio |  | 8.67 | 0.003 |
| 88 | E(AAC)-H/M(TCG)-220 |  |  | Ratio |  | 5.04 | 0.049 |
| 92 | E(AAC)-H/M(TCG)-225 |  |  | Ratio |  | 4.23 | 0.038 |
| 111 | E(AAC)-H/M(TCG)-254 | Leaf length |  |  |  | 3.85 | 0.029 |
|  | E(AAC)-H/M(TCG)-254 |  |  |  | Leaf area | 4.29 | 0.020 |
| 112 | E(AAC)-H/M(TCG)-258 | Leaf length |  |  |  | 4.83 | 0.010 |
|  | E(AAC)-H/M(TCG)-258 |  |  |  | Leaf area | 4.90 | 0.010 |
| 115 | E(AAC)-H/M(TCG)-266 |  |  |  | Leaf area | 3.47 | 0.049 |
| 132 | E(AAC)-H/M(TCG)-311 | Leaf length |  |  |  | 3.94 | 0.026 |
|  | E(AAC)-H/M(TCG)-311 |  |  |  | Leaf area | 3.64 | 0.041 |
| 137 | E(AAC)-H/M(TCG)-346 | Leaf length |  |  |  | 3.61 | 0.038 |
|  | E(AAC)-H/M(TCG)-346 |  | Leaf width |  |  | 4.51 | 0.020 |
|  | E(AAC)-H/M(TCG)-346 |  |  |  | Leaf area | 4.36 | 0.018 |
| 141 | E(AAC)-H/M(TCG)-379 | Leaf length |  |  |  | 3.57 | 0.040 |
|  | E(AAC)-H/M(TCG)-379 |  | Leaf width |  |  | 3.99 | 0.034 |
|  | E(AAC)-H/M(TCG)-379 |  |  |  | Leaf area | 3.80 | 0.034 |
| 143 | E(AAC)-H/M(TCG)-389 | Leaf length |  |  |  | 3.69 | 0.013 |
|  | E(AAC)-H/M(TCG)-389 |  |  |  | Leaf area | 3.99 | 0.010 |
| 144 | E(AAC)-H/M(TCG)-427 | Leaf length |  |  |  | 4.69 | 0.011 |
|  | E(AAC)-H/M(TCG)-427 |  | Leaf width |  |  | 5.11 | 0.010 |
|  | E(AAC)-H/M(TCG)-427 |  |  |  | Leaf area | 5.08 | 0.008 |
| 150 | E(AAC)-H/M(TCG)-470 |  |  | Ratio |  | 4.22 | 0.038 |
| 151 | E(AAC)-H/M(TCG)-492 |  |  | Ratio |  | 4.22 | 0.038 |
| 167 | E(AAC)-H/M(TTC)-086 |  |  | Ratio |  | 4.35 | 0.034 |
| 169 | E(AAC)-H/M(TTC)-089 |  |  | Ratio |  | 6.60 | 0.015 |
| 176 | E(AAC)-H/M(TTC)-101 |  |  | Ratio |  | 5.18 | 0.045 |
| 179 | E(AAC)-H/M(TTC)-106 |  |  | Ratio |  | 5.77 | 0.029 |
| 193 | E(AAC)-H/M(TTC)-131 | Leaf length |  |  |  | 3.51 | 0.042 |
| 205 | E(AAC)-H/M(TTC)-150 |  |  | Ratio |  | 5.68 | 0.031 |
| 208 | E(AAC)-H/M(TTC)-155 |  | Leaf width |  |  | 4.57 | 0.019 |
| 248 | E(AAC)-H/M(TTC)-238 |  |  | Ratio |  | 6.51 | 0.017 |
| 319 | E(AAC)-H/M(TTC)-441 |  | Leaf width |  |  | 4.80 | 0.015 |
| 327 | E(AAC)-H/M(TTC)-476 |  | Leaf width |  |  | 3.98 | 0.035 |
| 329 | E(AAC)-H/M(TTC)-478 |  | Leaf width |  |  | 5.12 | 0.010 |
| 334 | E(AAC)-H/M(TTC)-504 |  | Leaf width |  |  | 4.69 | 0.017 |
| 339 | E(AAC)-H/M(TTC)-520 |  | Leaf width |  |  | 4.95 | 0.013 |
| 340 | E(AAC)-H/M(TTC)-525 |  | Leaf width |  |  | 4.95 | 0.013 |
| 341 | E(AAC)-H/M(TTC)-526 |  | Leaf width |  |  | 4.97 | 0.012 |
| 342 | E(AAC)-H/M(TTC)-528 |  | Leaf width |  |  | 6.43 | 0.002 |
|  | E(AAC)-H/M(TTC)-528 |  |  |  | Leaf area | 3.80 | 0.034 |
| 343 | E(AAC)-H/M(TTC)-533 |  |  | Ratio |  | 5.83 | 0.028 |
| 344 | E(AAC)-H/M(TTC)-536 |  |  | Ratio |  | 2.54 | 0.048 |
| 345 | E(AAC)-H/M(TTC)-564 |  |  | Ratio |  | 4.79 | 0.006 |
| 346 | E(AAC)-H/M(TTC)-578 |  |  | Ratio |  | 2.68 | 0.042 |
| 347 | E(AAC)-H/M(TTC)-586 |  |  | Ratio |  | 4.79 | 0.006 |
| 350 | E(AAG)-H/M(TTG)-068 | Leaf length |  |  |  | 5.10 | 0.007 |
|  | E(AAG)-H/M(TTG)-068 |  | Leaf width |  |  | 5.85 | 0.005 |
|  | E(AAG)-H/M(TTG)-068 |  |  | Ratio |  | 7.05 | 0.011 |
|  | E(AAG)-H/M(TTG)-068 |  |  |  | Leaf area | 4.14 | 0.024 |
| 351 | E(AAG)-H/M(TTG)-069 | Leaf length |  |  |  | 5.04 | 0.007 |
|  | E(AAG)-H/M(TTG)-069 |  | Leaf width |  |  | 3.72 | 0.045 |
|  | E(AAG)-H/M(TTG)-069 |  |  |  | Leaf area | 3.65 | 0.040 |
| 354 | E(AAG)-H/M(TTG)-075 | Leaf length |  |  |  | 4.77 | 0.010 |
| 358 | E(AAG)-H/M(TTG)-082 | Leaf length |  |  |  | 5.19 | 0.006 |
|  | E(AAG)-H/M(TTG)-082 |  |  |  | Leaf area | 3.90 | 0.031 |
| 361 | E(AAG)-H/M(TTG)-087 |  |  | Ratio |  | 5.60 | 0.033 |
| 365 | E(AAG)-H/M(TTG)-094 |  | Leaf width |  |  | 4.38 | 0.023 |
| 372 | E(AAG)-H/M(TTG)-103 |  |  | Ratio |  | 5.79 | 0.028 |
| 374 | E(AAG)-H/M(TTG)-106 |  |  | Ratio |  | 5.10 | 0.048 |
| 385 | E(AAG)-H/M(TTG)-121 | Leaf length |  |  |  | 4.21 | 0.019 |
|  | E(AAG)-H/M(TTG)-121 |  | Leaf width |  |  | 5.01 | 0.012 |
|  | E(AAG)-H/M(TTG)-121 |  |  | Ratio |  | 6.34 | 0.019 |
|  | E(AAG)-H/M(TTG)-121 |  |  |  | Leaf area | 3.98 | 0.028 |
| 391 | E(AAG)-H/M(TTG)-130 |  | Leaf width |  |  | 4.80 | 0.015 |
|  | E(AAG)-H/M(TTG)-130 |  |  |  | Leaf area | 3.83 | 0.033 |
| 400 | E(AAG)-H/M(TTG)-146 |  | Leaf width |  |  | 4.15 | 0.029 |
| 402 | E(AAG)-H/M(TTG)-149 | Leaf length |  |  |  | 4.77 | 0.010 |
|  | E(AAG)-H/M(TTG)-149 |  | Leaf width |  |  | 4.24 | 0.026 |
| 404 | E(AAG)-H/M(TTG)-153 |  |  | Ratio |  | 11.43 | 0.000 |
| 406 | E(AAG)-H/M(TTG)-159 | Leaf length |  |  |  | 3.62 | 0.038 |
| 410 | E(AAG)-H/M(TTG)-165 |  |  | Ratio |  | 8.07 | 0.005 |
| 413 | E(AAG)-H/M(TTG)-172 | Leaf length |  |  |  | 3.37 | 0.049 |
| 429 | E(AAG)-H/M(TTG)-211 | Leaf length |  |  |  | 4.16 | 0.021 |
|  | E(AAG)-H/M(TTG)-211 |  | Leaf width |  |  | 4.93 | 0.013 |
|  | E(AAG)-H/M(TTG)-211 |  |  |  | Leaf area | 4.65 | 0.013 |
| 430 | E(AAG)-H/M(TTG)-212 | Leaf length |  |  |  | 3.37 | 0.049 |
| 432 | E(AAG)-H/M(TTG)-216 |  |  | Ratio |  | 6.82 | 0.013 |
| 434 | E(AAG)-H/M(TTG)-220 |  | Leaf width |  |  | 3.89 | 0.038 |
|  | E(AAG)-H/M(TTG)-220 |  |  |  | Leaf area | 3.48 | 0.048 |
| 435 | E(AAG)-H/M(TTG)-222 |  |  | Ratio |  | 6.84 | 0.013 |
| 439 | E(AAG)-H/M(TTG)-229 |  |  | Ratio |  | 5.19 | 0.044 |
| 440 | E(AAG)-H/M(TTG)-231 |  | Leaf width |  |  | 4.06 | 0.032 |
|  | E(AAG)-H/M(TTG)-231 |  |  | Ratio |  | 7.50 | 0.008 |
| 448 | E(AAG)-H/M(TTG)-249 |  |  | Ratio |  | 6.14 | 0.022 |
| 465 | E(AAG)-H/M(TTG)-294 |  | Leaf width |  |  | 4.06 | 0.032 |
|  | E(AAG)-H/M(TTG)-294 |  |  | Ratio |  | 6.44 | 0.017 |
| 466 | E(AAG)-H/M(TTG)-297 |  |  | Ratio |  | 6.08 | 0.023 |
| 473 | E(AAG)-H/M(TTG)-315 | Leaf length |  |  |  | 3.45 | 0.046 |
|  | E(AAG)-H/M(TTG)-315 |  |  |  | Leaf area | 3.47 | 0.049 |
| 481 | E(AAG)-H/M(TTG)-334 |  |  | Ratio |  | 6.27 | 0.020 |
| 489 | E(AAG)-H/M(TTG)-349 |  |  | Ratio |  | 8.47 | 0.004 |
| 491 | E(AAG)-H/M(TTG)-356 |  |  | Ratio |  | 5.94 | 0.025 |
| 499 | E(AAG)-H/M(TTG)-372 | Leaf length |  |  |  | 3.38 | 0.049 |
| 501 | E(AAG)-H/M(TTG)-381 | Leaf length |  |  |  | 5.76 | 0.003 |
|  | E(AAG)-H/M(TTG)-381 |  | Leaf width |  |  | 5.10 | 0.011 |
|  | E(AAG)-H/M(TTG)-381 |  |  |  | Leaf area | 4.20 | 0.022 |
| 507 | E(AAG)-H/M(TTG)-392 |  | Leaf width |  |  | 3.89 | 0.038 |
| 508 | E(AAG)-H/M(TTG)-394 |  | Leaf width |  |  | 4.53 | 0.019 |
| 510 | E(AAG)-H/M(TTG)-397 |  | Leaf width |  |  | 4.40 | 0.022 |
|  | E(AAG)-H/M(TTG)-397 |  |  | Ratio |  | 7.42 | 0.008 |
| 513 | E(AAG)-H/M(TTG)-408 | Leaf length |  |  |  | 3.84 | 0.029 |
| 518 | E(AAG)-H/M(TTG)-424 |  |  | Ratio |  | 5.21 | 0.044 |
| 519 | E(AAG)-H/M(TTG)-426 | Leaf length |  |  |  | 3.72 | 0.034 |
|  | E(AAG)-H/M(TTG)-426 |  | Leaf width |  |  | 4.67 | 0.017 |
|  | E(AAG)-H/M(TTG)-426 |  |  | Ratio |  | 6.03 | 0.024 |
|  | E(AAG)-H/M(TTG)-426 |  |  |  | Leaf area | 3.69 | 0.038 |
| 525 | E(AAG)-H/M(TTG)-440 |  | Leaf width |  |  | 4.55 | 0.019 |
|  | E(AAG)-H/M(TTG)-440 |  |  |  | Leaf area | 4.40 | 0.018 |
| 534 | E(AAG)-H/M(TTG)-460 | Leaf length |  |  |  | 3.86 | 0.029 |
|  | E(AAG)-H/M(TTG)-460 |  |  | Ratio |  | 5.85 | 0.027 |
| 540 | E(AAG)-H/M(TTG)-477 | Leaf length |  |  |  | 4.03 | 0.024 |
| 541 | E(AAG)-H/M(TTG)-479 | Leaf length |  |  |  | 3.61 | 0.038 |
| 572 | E(AAG)-H/M(TTA)-070 |  |  | Ratio |  | 5.33 | 0.040 |
| 584 | E(AAG)-H/M(TTA)-094 |  |  | Ratio |  | 6.96 | 0.012 |
| 588 | E(AAG)-H/M(TTA)-101 |  |  | Ratio |  | 6.41 | 0.018 |
| 610 | E(AAG)-H/M(TTA)-136 | Leaf length |  |  |  | 4.19 | 0.020 |
|  | E(AAG)-H/M(TTA)-136 |  | Leaf width |  |  | 4.73 | 0.016 |
|  | E(AAG)-H/M(TTA)-136 |  |  |  | Leaf area | 4.14 | 0.024 |
| 619 | E(AAG)-H/M(TTA)-154 |  |  | Ratio |  | 8.28 | 0.004 |
| 621 | E(AAG)-H/M(TTA)-157 | Leaf length |  |  |  | 3.65 | 0.037 |
|  | E(AAG)-H/M(TTA)-157 |  | Leaf width |  |  | 5.00 | 0.012 |
|  | E(AAG)-H/M(TTA)-157 |  |  |  | Leaf area | 4.70 | 0.013 |
| 658 | E(AAG)-H/M(TTA)-242 | Leaf length |  |  |  | 3.93 | 0.027 |
|  | E(AAG)-H/M(TTA)-242 |  |  |  | Leaf area | 3.50 | 0.047 |
| 662 | E(AAG)-H/M(TTA)-247 |  |  | Ratio |  | 4.45 | 0.031 |
| 671 | E(AAG)-H/M(TTA)-270 | Leaf length |  |  |  | 3.38 | 0.049 |
|  | E(AAG)-H/M(TTA)-270 |  | Leaf width |  |  | 4.76 | 0.015 |
|  | E(AAG)-H/M(TTA)-270 |  |  |  | Leaf area | 5.13 | 0.008 |
| 707 | E(AAG)-H/M(TTA)-575 |  | Leaf width |  |  | 1.83 | 0.048 |
|  | E(AAG)-H/M(TTA)-575 |  |  |  | Leaf area | 1.76 | 0.047 |
| 709 | E(AAG)-H/M(TTA)-587 |  | Leaf width |  |  | 2.37 | 0.024 |
|  | E(AAG)-H/M(TTA)-587 |  |  |  | Leaf area | 1.89 | 0.039 |
| 723 | E(ACA)-H/M(TCG)-081 | Leaf length |  |  |  | 3.71 | 0.034 |
| 725 | E(ACA)-H/M(TCG)-084 |  |  | Ratio |  | 5.97 | 0.025 |
| 728 | E(ACA)-H/M(TCG)-088 | Leaf length |  |  |  | 3.46 | 0.045 |
|  | E(ACA)-H/M(TCG)-088 |  |  |  | Leaf area | 3.90 | 0.031 |
| 729 | E(ACA)-H/M(TCG)-090 |  |  | Ratio |  | 15.42 | 0.000 |
| 733 | E(ACA)-H/M(TCG)-096 |  |  | Ratio |  | 7.56 | 0.007 |
| 749 | E(ACA)-H/M(TCG)-118 |  |  | Ratio |  | 5.14 | 0.046 |
| 760 | E(ACA)-H/M(TCG)-135 | Leaf length |  |  |  | 4.41 | 0.015 |
|  | E(ACA)-H/M(TCG)-135 |  | Leaf width |  |  | 4.35 | 0.024 |
|  | E(ACA)-H/M(TCG)-135 |  |  |  | Leaf area | 4.41 | 0.018 |
| 764 | E(ACA)-H/M(TCG)-142 |  |  | Ratio |  | 7.59 | 0.007 |
| 767 | E(ACA)-H/M(TCG)-147 |  |  | Ratio |  | 5.43 | 0.037 |
| 769 | E(ACA)-H/M(TCG)-149 | Leaf length |  |  |  | 4.18 | 0.020 |
|  | E(ACA)-H/M(TCG)-149 |  | Leaf width |  |  | 4.81 | 0.014 |
|  | E(ACA)-H/M(TCG)-149 |  |  |  | Leaf area | 3.90 | 0.031 |
| 772 | E(ACA)-H/M(TCG)-154 |  |  | Ratio |  | 5.18 | 0.045 |
| 773 | E(ACA)-H/M(TCG)-156 |  |  | Ratio |  | 6.61 | 0.015 |
| 796 | E(ACA)-H/M(TCG)-193 | Leaf length |  |  |  | 5.76 | 0.003 |
|  | E(ACA)-H/M(TCG)-193 |  | Leaf width |  |  | 4.48 | 0.021 |
|  | E(ACA)-H/M(TCG)-193 |  |  |  | Leaf area | 4.83 | 0.011 |
| 798 | E(ACA)-H/M(TCG)-196 |  |  |  | Leaf area | 3.90 | 0.031 |
| 814 | E(ACA)-H/M(TCG)-223 | Leaf length |  |  |  | 3.37 | 0.049 |
| 820 | E(ACA)-H/M(TCG)-231 |  |  | Ratio |  | 5.62 | 0.032 |
| 824 | E(ACA)-H/M(TCG)-237 | Leaf length |  |  |  | 5.68 | 0.003 |
|  | E(ACA)-H/M(TCG)-237 |  | Leaf width |  |  | 4.31 | 0.024 |
|  | E(ACA)-H/M(TCG)-237 |  |  |  | Leaf area | 4.78 | 0.012 |
| 825 | E(ACA)-H/M(TCG)-238 | Leaf length |  |  |  | 3.93 | 0.027 |
|  | E(ACA)-H/M(TCG)-238 |  |  |  | Leaf area | 3.67 | 0.039 |
| 826 | E(ACA)-H/M(TCG)-239 | Leaf length |  |  |  | 5.94 | 0.003 |
|  | E(ACA)-H/M(TCG)-239 |  | Leaf width |  |  | 5.79 | 0.005 |
|  | E(ACA)-H/M(TCG)-239 |  |  |  | Leaf area | 5.89 | 0.003 |
| 827 | E(ACA)-H/M(TCG)-240 | Leaf length |  |  |  | 8.05 | 0.000 |
|  | E(ACA)-H/M(TCG)-240 |  | Leaf width |  |  | 8.06 | 0.000 |
|  | E(ACA)-H/M(TCG)-240 |  |  |  | Leaf area | 7.65 | 0.000 |
| 830 | E(ACA)-H/M(TCG)-245 |  |  | Ratio |  | 5.09 | 0.048 |
| 832 | E(ACA)-H/M(TCG)-251 | Leaf length |  |  |  | 7.37 | 0.000 |
|  | E(ACA)-H/M(TCG)-251 |  |  | Ratio |  | 8.61 | 0.003 |
|  | E(ACA)-H/M(TCG)-251 |  |  |  | Leaf area | 5.22 | 0.007 |
| 850 | E(ACA)-H/M(TCG)-283 | Leaf length |  |  |  | 3.75 | 0.033 |
| 852 | E(ACA)-H/M(TCG)-285 | Leaf length |  |  |  | 3.86 | 0.029 |
|  | E(ACA)-H/M(TCG)-285 |  | Leaf width |  |  | 4.50 | 0.020 |
|  | E(ACA)-H/M(TCG)-285 |  |  |  | Leaf area | 4.06 | 0.026 |
| 853 | E(ACA)-H/M(TCG)-286 | Leaf length |  |  |  | 4.96 | 0.008 |
|  | E(ACA)-H/M(TCG)-286 |  | Leaf width |  |  | 5.41 | 0.008 |
|  | E(ACA)-H/M(TCG)-286 |  |  | Ratio |  | 5.24 | 0.043 |
|  | E(ACA)-H/M(TCG)-286 |  |  |  | Leaf area | 3.89 | 0.031 |
| 854 | E(ACA)-H/M(TCG)-287 | Leaf length |  |  |  | 3.42 | 0.047 |
| 860 | E(ACA)-H/M(TCG)-305 | Leaf length |  |  |  | 4.72 | 0.011 |
|  | E(ACA)-H/M(TCG)-305 |  | Leaf width |  |  | 4.73 | 0.016 |
|  | E(ACA)-H/M(TCG)-305 |  |  |  | Leaf area | 4.67 | 0.013 |
| 864 | E(ACA)-H/M(TCG)-319 | Leaf length |  |  |  | 3.86 | 0.029 |
|  | E(ACA)-H/M(TCG)-319 |  | Leaf width |  |  | 3.87 | 0.039 |
|  | E(ACA)-H/M(TCG)-319 |  |  |  | Leaf area | 3.91 | 0.030 |
| 879 | E(ACA)-H/M(TCG)-369 |  |  | Ratio |  | 5.64 | 0.032 |
| 926 | E(ACA)-H/M(TTG)-068 | Leaf length |  |  |  | 6.19 | 0.002 |
|  | E(ACA)-H/M(TTG)-068 |  | Leaf width |  |  | 4.46 | 0.021 |
|  | E(ACA)-H/M(TTG)-068 |  |  |  | Leaf area | 4.72 | 0.012 |
| 927 | E(ACA)-H/M(TTG)-069 |  |  |  | Leaf area | 3.62 | 0.042 |
| 934 | E(ACA)-H/M(TTG)-082 |  |  | Ratio |  | 5.48 | 0.036 |
| 939 | E(ACA)-H/M(TTG)-090 | Leaf length |  |  |  | 5.70 | 0.003 |
|  | E(ACA)-H/M(TTG)-090 |  | Leaf width |  |  | 5.58 | 0.006 |
|  | E(ACA)-H/M(TTG)-090 |  |  |  | Leaf area | 6.24 | 0.002 |
| 941 | E(ACA)-H/M(TTG)-094 |  |  | Ratio |  | 5.05 | 0.049 |
| 943 | E(ACA)-H/M(TTG)-096 |  | Leaf width |  |  | 5.38 | 0.008 |
|  | E(ACA)-H/M(TTG)-096 |  |  |  | Leaf area | 3.76 | 0.036 |
| 950 | E(ACA)-H/M(TTG)-106 |  |  | Ratio |  | 6.78 | 0.013 |
| 988 | E(ACA)-H/M(TTG)-168 |  |  | Ratio |  | 5.22 | 0.043 |
| 992 | E(ACA)-H/M(TTG)-173 |  |  | Ratio |  | 7.44 | 0.008 |
| 993 | E(ACA)-H/M(TTG)-174 | Leaf length |  |  |  | 3.92 | 0.027 |
| 998 | E(ACA)-H/M(TTG)-183 | Leaf length |  |  |  | 4.12 | 0.022 |
|  | E(ACA)-H/M(TTG)-183 |  |  |  | Leaf area | 4.73 | 0.012 |
| 999 | E(ACA)-H/M(TTG)-185 | Leaf length |  |  |  | 4.46 | 0.015 |
|  | E(ACA)-H/M(TTG)-185 |  | Leaf width |  |  | 3.83 | 0.041 |
|  | E(ACA)-H/M(TTG)-185 |  |  |  | Leaf area | 4.13 | 0.024 |
| 1010 | E(ACA)-H/M(TTG)-204 |  |  | Ratio |  | 5.48 | 0.036 |
| 1014 | E(ACA)-H/M(TTG)-211 | Leaf length |  |  |  | 3.45 | 0.045 |
| 1020 | E(ACA)-H/M(TTG)-222 |  |  | Ratio |  | 5.24 | 0.043 |
| 1021 | E(ACA)-H/M(TTG)-223 |  | Leaf width |  |  | 4.42 | 0.022 |
| 1025 | E(ACA)-H/M(TTG)-232 | Leaf length |  |  |  | 4.13 | 0.021 |
|  | E(ACA)-H/M(TTG)-232 |  | Leaf width |  |  | 3.90 | 0.038 |
|  | E(ACA)-H/M(TTG)-232 |  |  |  | Leaf area | 3.55 | 0.045 |
| 1027 | E(ACA)-H/M(TTG)-235 | Leaf length |  |  |  | 6.91 | 0.001 |
|  | E(ACA)-H/M(TTG)-235 |  | Leaf width |  |  | 6.69 | 0.002 |
|  | E(ACA)-H/M(TTG)-235 |  |  |  | Leaf area | 5.69 | 0.004 |
| 1028 | E(ACA)-H/M(TTG)-236 | Leaf length |  |  |  | 3.45 | 0.046 |
| 1033 | E(ACA)-H/M(TTG)-251 | Leaf length |  |  |  | 4.07 | 0.023 |
|  | E(ACA)-H/M(TTG)-251 |  | Leaf width |  |  | 6.32 | 0.003 |
|  | E(ACA)-H/M(TTG)-251 |  |  |  | Leaf area | 4.95 | 0.010 |
| 1042 | E(ACA)-H/M(TTG)-270 | Leaf length |  |  |  | 3.85 | 0.029 |
| 1046 | E(ACA)-H/M(TTG)-290 |  |  | Ratio |  | 5.33 | 0.040 |
| 1047 | E(ACA)-H/M(TTG)-293 | Leaf length |  |  |  | 3.67 | 0.036 |
|  | E(ACA)-H/M(TTG)-293 |  |  | Ratio |  | 6.71 | 0.014 |
| 1050 | E(ACA)-H/M(TTG)-298 |  | Leaf width |  |  | 5.16 | 0.010 |
|  | E(ACA)-H/M(TTG)-298 |  |  |  | Leaf area | 4.35 | 0.019 |
| 1053 | E(ACA)-H/M(TTG)-304 | Leaf length |  |  |  | 4.63 | 0.012 |
|  | E(ACA)-H/M(TTG)-304 |  | Leaf width |  |  | 4.90 | 0.013 |
|  | E(ACA)-H/M(TTG)-304 |  |  |  | Leaf area | 4.96 | 0.009 |
| 1066 | E(ACA)-H/M(TTG)-336 | Leaf length |  |  |  | 3.42 | 0.047 |
| 1068 | E(ACA)-H/M(TTG)-339 |  |  | Ratio |  | 6.42 | 0.018 |
| 1070 | E(ACA)-H/M(TTG)-342 |  |  | Ratio |  | 8.41 | 0.004 |
| 1071 | E(ACA)-H/M(TTG)-345 |  |  | Ratio |  | 7.43 | 0.008 |
| 1073 | E(ACA)-H/M(TTG)-347 |  |  | Ratio |  | 7.51 | 0.008 |
| 1074 | E(ACA)-H/M(TTG)-350 |  |  | Ratio |  | 11.14 | 0.000 |
| 1075 | E(ACA)-H/M(TTG)-353 |  |  | Ratio |  | 7.12 | 0.010 |
| 1076 | E(ACA)-H/M(TTG)-355 |  |  | Ratio |  | 7.54 | 0.007 |
| 1077 | E(ACA)-H/M(TTG)-358 |  |  | Ratio |  | 11.02 | 0.000 |
| 1078 | E(ACA)-H/M(TTG)-360 |  |  | Ratio |  | 5.59 | 0.033 |
| 1079 | E(ACA)-H/M(TTG)-362 |  |  | Ratio |  | 9.93 | 0.001 |
| 1080 | E(ACA)-H/M(TTG)-366 |  |  | Ratio |  | 6.61 | 0.015 |
| 1081 | E(ACA)-H/M(TTG)-368 |  |  | Ratio |  | 7.88 | 0.006 |
| 1082 | E(ACA)-H/M(TTG)-369 |  |  | Ratio |  | 5.18 | 0.045 |
| 1084 | E(ACA)-H/M(TTG)-373 |  |  | Ratio |  | 10.05 | 0.001 |
| 1085 | E(ACA)-H/M(TTG)-376 |  |  | Ratio |  | 5.76 | 0.029 |
| 1095 | E(ACA)-H/M(TTG)-415 |  |  | Ratio |  | 5.91 | 0.010 |
| 1096 | E(ACA)-H/M(TTG)-421 |  | Leaf width |  |  | 3.75 | 0.044 |
| 1105 | E(ACA)-H/M(TTG)-466 |  |  | Ratio |  | 5.21 | 0.044 |
| 1109 | E(ACA)-H/M(TTG)-503 |  |  | Ratio |  | 5.09 | 0.048 |
| 1110 | E(ACA)-H/M(TTG)-504 |  |  | Ratio |  | 4.01 | 0.045 |
| 1122 | E(ACA)-H/M(TTG)-550 | Leaf length |  |  |  | 3.77 | 0.012 |
|  | E(ACA)-H/M(TTG)-550 |  |  |  | Leaf area | 2.98 | 0.034 |
| 1123 | E(ACA)-H/M(TTG)-555 | Leaf length |  |  |  | 2.30 | 0.021 |
|  | E(ACA)-H/M(TTG)-555 |  | Leaf width |  |  | 2.74 | 0.015 |
|  | E(ACA)-H/M(TTG)-555 |  |  |  | Leaf area | 2.41 | 0.019 |
| 1128 | E(ACA)-H/M(TTG)-565 | Leaf length |  |  |  | 2.30 | 0.021 |
|  | E(ACA)-H/M(TTG)-565 |  | Leaf width |  |  | 2.74 | 0.015 |
|  | E(ACA)-H/M(TTG)-565 |  |  |  | Leaf area | 2.41 | 0.019 |
| 1136 | E(ACC)-H/M(TCC)-073 | Leaf length |  |  |  | 4.83 | 0.010 |
|  | E(ACC)-H/M(TCC)-073 |  |  |  | Leaf area | 4.18 | 0.023 |
| 1151 | E(ACC)-H/M(TCC)-099 | Leaf length |  |  |  | 4.13 | 0.021 |
|  | E(ACC)-H/M(TCC)-099 |  | Leaf width |  |  | 4.06 | 0.032 |
|  | E(ACC)-H/M(TCC)-099 |  |  |  | Leaf area | 4.44 | 0.017 |
| 1156 | E(ACC)-H/M(TCC)-107 | Leaf length |  |  |  | 4.85 | 0.009 |
|  | E(ACC)-H/M(TCC)-107 |  |  |  | Leaf area | 3.77 | 0.036 |
| 1163 | E(ACC)-H/M(TCC)-119 |  |  | Ratio |  | 7.43 | 0.008 |
| 1166 | E(ACC)-H/M(TCC)-123 |  | Leaf width |  |  | 3.78 | 0.042 |
| 1174 | E(ACC)-H/M(TCC)-136 |  |  | Ratio |  | 5.93 | 0.026 |
| 1177 | E(ACC)-H/M(TCC)-142 |  | Leaf width |  |  | 4.62 | 0.018 |
| 1184 | E(ACC)-H/M(TCC)-154 |  | Leaf width |  |  | 3.96 | 0.035 |
|  | E(ACC)-H/M(TCC)-154 |  |  |  | Leaf area | 3.48 | 0.049 |
| 1205 | E(ACC)-H/M(TCC)-186 |  | Leaf width |  |  | 6.11 | 0.004 |
|  | E(ACC)-H/M(TCC)-186 |  |  |  | Leaf area | 4.46 | 0.017 |
| 1206 | E(ACC)-H/M(TCC)-188 | Leaf length |  |  |  | 3.99 | 0.025 |
|  | E(ACC)-H/M(TCC)-188 |  | Leaf width |  |  | 6.42 | 0.002 |
|  | E(ACC)-H/M(TCC)-188 |  |  |  | Leaf area | 5.81 | 0.004 |
| 1209 | E(ACC)-H/M(TCC)-192 |  |  | Ratio |  | 5.08 | 0.048 |
| 1221 | E(ACC)-H/M(TCC)-210 |  | Leaf width |  |  | 3.73 | 0.045 |
|  | E(ACC)-H/M(TCC)-210 |  |  |  | Leaf area | 4.15 | 0.023 |
| 1232 | E(ACC)-H/M(TCC)-231 | Leaf length |  |  |  | 3.45 | 0.045 |
| 1234 | E(ACC)-H/M(TCC)-239 |  |  | Ratio |  | 6.03 | 0.024 |
| 1235 | E(ACC)-H/M(TCC)-242 | Leaf length |  |  |  | 3.60 | 0.038 |
|  | E(ACC)-H/M(TCC)-242 |  |  |  | Leaf area | 3.99 | 0.028 |
| 1254 | E(ACC)-H/M(TCC)-291 |  |  |  | Leaf area | 3.66 | 0.040 |
| 1255 | E(ACC)-H/M(TCC)-293 |  |  | Ratio |  | 8.05 | 0.005 |
| 1260 | E(ACC)-H/M(TCC)-315 | Leaf length |  |  |  | 3.48 | 0.044 |
| 1262 | E(ACC)-H/M(TCC)-322 |  |  |  | Leaf area | 3.47 | 0.049 |
| 1270 | E(ACC)-H/M(TCC)-340 |  |  | Ratio |  | 6.52 | 0.016 |
| 1272 | E(ACC)-H/M(TCC)-347 | Leaf length |  |  |  | 4.41 | 0.016 |
|  | E(ACC)-H/M(TCC)-347 |  |  | Ratio |  | 6.62 | 0.015 |
|  | E(ACC)-H/M(TCC)-347 |  |  |  | Leaf area | 3.58 | 0.044 |
| 1274 | E(ACC)-H/M(TCC)-358 |  | Leaf width |  |  | 4.15 | 0.029 |
|  | E(ACC)-H/M(TCC)-358 |  |  |  | Leaf area | 3.61 | 0.042 |
| 1277 | E(ACC)-H/M(TCC)-370 | Leaf length |  |  |  | 3.62 | 0.038 |
|  | E(ACC)-H/M(TCC)-370 |  |  |  | Leaf area | 3.57 | 0.044 |
| 1280 | E(ACC)-H/M(TCC)-376 |  |  | Ratio |  | 6.45 | 0.017 |
| 1281 | E(ACC)-H/M(TCC)-381 | Leaf length |  |  |  | 4.28 | 0.018 |
|  | E(ACC)-H/M(TCC)-381 |  |  |  | Leaf area | 4.09 | 0.025 |
| 1282 | E(ACC)-H/M(TCC)-384 | Leaf length |  |  |  | 3.63 | 0.037 |
|  | E(ACC)-H/M(TCC)-384 |  |  | Ratio |  | 6.09 | 0.023 |
| 1284 | E(ACC)-H/M(TCC)-388 | Leaf length |  |  |  | 3.63 | 0.037 |
|  | E(ACC)-H/M(TCC)-388 |  |  | Ratio |  | 5.69 | 0.031 |
| 1285 | E(ACC)-H/M(TCC)-393 | Leaf length |  |  |  | 3.98 | 0.025 |
|  | E(ACC)-H/M(TCC)-393 |  |  | Ratio |  | 5.80 | 0.028 |
| 1299 | E(ACC)-H/M(TCC)-491 |  | Leaf width |  |  | 2.89 | 0.045 |
|  | E(ACC)-H/M(TCC)-491 |  |  |  | Leaf area | 2.69 | 0.048 |
| 1310 | E(ACC)-H/M(TCC)-597 |  | Leaf width |  |  | 1.91 | 0.043 |
| 1343 | E(ACC)-H/M(TTC)-113 | Leaf length |  |  |  | 3.47 | 0.044 |
|  | E(ACC)-H/M(TTC)-113 |  | Leaf width |  |  | 4.95 | 0.012 |
|  | E(ACC)-H/M(TTC)-113 |  |  | Ratio |  | 6.82 | 0.013 |
|  | E(ACC)-H/M(TTC)-113 |  |  |  | Leaf area | 5.18 | 0.007 |
| 1344 | E(ACC)-H/M(TTC)-114 | Leaf length |  |  |  | 3.67 | 0.036 |
|  | E(ACC)-H/M(TTC)-114 |  |  |  | Leaf area | 3.73 | 0.037 |
| 1358 | E(ACC)-H/M(TTC)-135 |  | Leaf width |  |  | 3.90 | 0.038 |
| 1362 | E(ACC)-H/M(TTC)-141 | Leaf length |  |  |  | 4.34 | 0.017 |
| 1365 | E(ACC)-H/M(TTC)-144 | Leaf length |  |  |  | 3.45 | 0.045 |
| 1378 | E(ACC)-H/M(TTC)-164 | Leaf length |  |  |  | 5.39 | 0.005 |
|  | E(ACC)-H/M(TTC)-164 |  | Leaf width |  |  | 5.23 | 0.009 |
|  | E(ACC)-H/M(TTC)-164 |  |  |  | Leaf area | 4.94 | 0.010 |
| 1397 | E(ACC)-H/M(TTC)-195 |  |  | Ratio |  | 5.48 | 0.036 |
| 1401 | E(ACC)-H/M(TTC)-200 | Leaf length |  |  |  | 3.50 | 0.043 |
|  | E(ACC)-H/M(TTC)-200 |  |  |  | Leaf area | 3.75 | 0.036 |
| 1402 | E(ACC)-H/M(TTC)-201 | Leaf length |  |  |  | 4.25 | 0.019 |
|  | E(ACC)-H/M(TTC)-201 |  |  |  | Leaf area | 3.85 | 0.032 |
| 1436 | E(ACC)-H/M(TTC)-274 |  | Leaf width |  |  | 3.83 | 0.040 |
| 1438 | E(ACC)-H/M(TTC)-279 | Leaf length |  |  |  | 3.64 | 0.037 |
|  | E(ACC)-H/M(TTC)-279 |  |  |  | Leaf area | 3.73 | 0.037 |
| 1466 | E(ACC)-H/M(TTC)-350 | Leaf length |  |  |  | 3.87 | 0.029 |
|  | E(ACC)-H/M(TTC)-350 |  | Leaf width |  |  | 4.70 | 0.016 |
|  | E(ACC)-H/M(TTC)-350 |  |  |  | Leaf area | 4.11 | 0.025 |
| 1501 | E(ACC)-H/M(TTC)-466 | Leaf length |  |  |  | 3.70 | 0.034 |
|  | E(ACC)-H/M(TTC)-466 |  | Leaf width |  |  | 4.01 | 0.033 |
| 1542 | E(ACG)-H/M(TCT)-072 |  | Leaf width |  |  | 2.80 | 0.049 |
| 1550 | E(ACG)-H/M(TCT)-090 |  |  | Ratio |  | 5.20 | 0.044 |
| 1563 | E(ACG)-H/M(TCT)-114 | Leaf length |  |  |  | 3.78 | 0.032 |
| 1568 | E(ACG)-H/M(TCT)-122 |  |  | Ratio |  | 7.58 | 0.007 |
| 1572 | E(ACG)-H/M(TCT)-129 | Leaf length |  |  |  | 3.51 | 0.043 |
|  | E(ACG)-H/M(TCT)-129 |  |  | Ratio |  | 5.53 | 0.034 |
| 1578 | E(ACG)-H/M(TCT)-141 |  | Leaf width |  |  | 4.35 | 0.024 |
|  | E(ACG)-H/M(TCT)-141 |  |  |  | Leaf area | 3.77 | 0.035 |
| 1590 | E(ACG)-H/M(TCT)-160 | Leaf length |  |  |  | 3.44 | 0.046 |
| 1602 | E(ACG)-H/M(TCT)-178 |  | Leaf width |  |  | 4.18 | 0.028 |
|  | E(ACG)-H/M(TCT)-178 |  |  |  | Leaf area | 3.95 | 0.029 |
| 1603 | E(ACG)-H/M(TCT)-179 | Leaf length |  |  |  | 3.97 | 0.025 |
| 1609 | E(ACG)-H/M(TCT)-189 | Leaf length |  |  |  | 4.23 | 0.019 |
|  | E(ACG)-H/M(TCT)-189 |  |  | Ratio |  | 5.92 | 0.026 |
| 1624 | E(ACG)-H/M(TCT)-228 |  |  | Ratio |  | 5.39 | 0.038 |
| 1640 | E(ACG)-H/M(TCT)-267 | Leaf length |  |  |  | 3.64 | 0.037 |
| 1645 | E(ACG)-H/M(TCT)-281 | Leaf length |  |  |  | 4.86 | 0.009 |
|  | E(ACG)-H/M(TCT)-281 |  | Leaf width |  |  | 4.81 | 0.014 |
|  | E(ACG)-H/M(TCT)-281 |  |  |  | Leaf area | 4.74 | 0.012 |
| 1679 | E(ACG)-H/M(TCT)-377 | Leaf length |  |  |  | 4.75 | 0.010 |
|  | E(ACG)-H/M(TCT)-377 |  |  | Ratio |  | 5.06 | 0.049 |
| 1680 | E(ACG)-H/M(TCT)-385 | Leaf length |  |  |  | 4.20 | 0.020 |
| 1714 | E(ACG)-H/M(TGT)-068 | Leaf length |  |  |  | 3.60 | 0.039 |
|  | E(ACG)-H/M(TGT)-068 |  | Leaf width |  |  | 3.86 | 0.039 |
|  | E(ACG)-H/M(TGT)-068 |  |  |  | Leaf area | 3.51 | 0.047 |
| 1716 | E(ACG)-H/M(TGT)-070 | Leaf length |  |  |  | 4.20 | 0.020 |
|  | E(ACG)-H/M(TGT)-070 |  |  |  | Leaf area | 3.90 | 0.031 |
| 1723 | E(ACG)-H/M(TGT)-081 |  |  |  | Leaf area | 4.23 | 0.021 |
| 1728 | E(ACG)-H/M(TGT)-088 |  |  | Ratio |  | 4.96 | 0.021 |
| 1737 | E(ACG)-H/M(TGT)-103 |  | Leaf width |  |  | 3.98 | 0.035 |
| 1738 | E(ACG)-H/M(TGT)-105 | Leaf length |  |  |  | 4.85 | 0.009 |
|  | E(ACG)-H/M(TGT)-105 |  | Leaf width |  |  | 3.78 | 0.043 |
|  | E(ACG)-H/M(TGT)-105 |  |  |  | Leaf area | 5.01 | 0.009 |
| 1739 | E(ACG)-H/M(TGT)-106 |  |  | Ratio |  | 5.85 | 0.027 |
| 1740 | E(ACG)-H/M(TGT)-107 |  |  | Ratio |  | 6.19 | 0.021 |
| 1750 | E(ACG)-H/M(TGT)-120 | Leaf length |  |  |  | 3.63 | 0.037 |
| 1760 | E(ACG)-H/M(TGT)-133 | Leaf length |  |  |  | 5.35 | 0.005 |
|  | E(ACG)-H/M(TGT)-133 |  | Leaf width |  |  | 4.99 | 0.012 |
|  | E(ACG)-H/M(TGT)-133 |  |  |  | Leaf area | 5.34 | 0.006 |
| 1761 | E(ACG)-H/M(TGT)-134 |  |  | Ratio |  | 5.70 | 0.030 |
| 1770 | E(ACG)-H/M(TGT)-151 | Leaf length |  |  |  | 4.84 | 0.009 |
|  | E(ACG)-H/M(TGT)-151 |  | Leaf width |  |  | 3.88 | 0.038 |
|  | E(ACG)-H/M(TGT)-151 |  |  |  | Leaf area | 4.36 | 0.019 |
| 1775 | E(ACG)-H/M(TGT)-159 | Leaf length |  |  |  | 3.38 | 0.049 |
| 1779 | E(ACG)-H/M(TGT)-165 |  |  | Ratio |  | 6.21 | 0.021 |
| 1803 | E(ACG)-H/M(TGT)-205 | Leaf length |  |  |  | 4.40 | 0.016 |
|  | E(ACG)-H/M(TGT)-205 |  | Leaf width |  |  | 4.06 | 0.032 |
|  | E(ACG)-H/M(TGT)-205 |  |  |  | Leaf area | 4.26 | 0.021 |
| 1805 | E(ACG)-H/M(TGT)-209 |  |  | Ratio |  | 5.48 | 0.036 |
| 1811 | E(ACG)-H/M(TGT)-219 | Leaf length |  |  |  | 3.83 | 0.030 |
|  | E(ACG)-H/M(TGT)-219 |  |  |  | Leaf area | 3.76 | 0.036 |
| 1821 | E(ACG)-H/M(TGT)-243 | Leaf length |  |  |  | 4.65 | 0.012 |
|  | E(ACG)-H/M(TGT)-243 |  | Leaf width |  |  | 5.36 | 0.008 |
|  | E(ACG)-H/M(TGT)-243 |  |  |  | Leaf area | 5.46 | 0.005 |
| 1823 | E(ACG)-H/M(TGT)-248 | Leaf length |  |  |  | 5.78 | 0.003 |
|  | E(ACG)-H/M(TGT)-248 |  |  |  | Leaf area | 4.28 | 0.020 |
| 1826 | E(ACG)-H/M(TGT)-253 | Leaf length |  |  |  | 4.12 | 0.022 |
|  | E(ACG)-H/M(TGT)-253 |  | Leaf width |  |  | 4.83 | 0.014 |
|  | E(ACG)-H/M(TGT)-253 |  |  |  | Leaf area | 5.05 | 0.009 |
| 1831 | E(ACG)-H/M(TGT)-261 | Leaf length |  |  |  | 3.44 | 0.046 |
|  | E(ACG)-H/M(TGT)-261 |  |  |  | Leaf area | 3.63 | 0.041 |
| 1837 | E(ACG)-H/M(TGT)-278 |  |  | Ratio |  | 5.21 | 0.044 |
| 1895 | E(ACG)-H/M(TGT)-439 |  | Leaf width |  |  | 3.73 | 0.044 |
| 1899 | E(ACG)-H/M(TGT)-454 | Leaf length |  |  |  | 2.65 | 0.046 |
| 1904 | E(ACG)-H/M(TGT)-468 |  | Leaf width |  |  | 3.76 | 0.043 |
| 1911 | E(ACG)-H/M(TGT)-483 |  |  | Ratio |  | 6.41 | 0.018 |
| 1913 | E(ACG)-H/M(TGT)-488 |  | Leaf width |  |  | 4.08 | 0.031 |
| 1914 | E(ACG)-H/M(TGT)-489 |  | Leaf width |  |  | 5.04 | 0.011 |
|  | E(ACG)-H/M(TGT)-489 |  |  | Ratio |  | 5.17 | 0.045 |
| 1915 | E(ACG)-H/M(TGT)-516 |  | Leaf width |  |  | 5.46 | 0.007 |
|  | E(ACG)-H/M(TGT)-516 |  |  |  | Leaf area | 3.57 | 0.044 |
| 1916 | E(ACG)-H/M(TGT)-528 | Leaf length |  |  |  | 4.24 | 0.019 |
|  | E(ACG)-H/M(TGT)-528 |  | Leaf width |  |  | 7.01 | 0.001 |
|  | E(ACG)-H/M(TGT)-528 |  |  |  | Leaf area | 4.59 | 0.014 |
| 1917 | E(ACG)-H/M(TGT)-539 |  | Leaf width |  |  | 4.82 | 0.014 |
| 1918 | E(ACG)-H/M(TGT)-541 | Leaf length |  |  |  | 3.90 | 0.028 |
|  | E(ACG)-H/M(TGT)-541 |  | Leaf width |  |  | 5.83 | 0.005 |
|  | E(ACG)-H/M(TGT)-541 |  |  |  | Leaf area | 4.08 | 0.025 |
| 1922 | E(ACT)-H/M(TTC)-063 |  | Leaf width |  |  | 3.74 | 0.044 |
| 1926 | E(ACT)-H/M(TTC)-070 | Leaf length |  |  |  | 3.88 | 0.028 |
|  | E(ACT)-H/M(TTC)-070 |  |  | Ratio |  | 6.37 | 0.018 |
| 1927 | E(ACT)-H/M(TTC)-072 | Leaf length |  |  |  | 3.43 | 0.046 |
| 1929 | E(ACT)-H/M(TTC)-076 | Leaf length |  |  |  | 3.00 | 0.031 |
|  | E(ACT)-H/M(TTC)-076 |  | Leaf width |  |  | 3.07 | 0.036 |
|  | E(ACT)-H/M(TTC)-076 |  |  |  | Leaf area | 3.44 | 0.020 |
| 1934 | E(ACT)-H/M(TTC)-087 | Leaf length |  |  |  | 7.68 | 0.000 |
|  | E(ACT)-H/M(TTC)-087 |  | Leaf width |  |  | 7.58 | 0.001 |
|  | E(ACT)-H/M(TTC)-087 |  |  |  | Leaf area | 5.98 | 0.003 |
| 1935 | E(ACT)-H/M(TTC)-090 |  |  | Ratio |  | 7.12 | 0.010 |
| 1946 | E(ACT)-H/M(TTC)-106 |  |  | Ratio |  | 5.14 | 0.046 |
| 1954 | E(ACT)-H/M(TTC)-119 |  |  | Ratio |  | 6.06 | 0.023 |
| 1971 | E(ACT)-H/M(TTC)-148 |  |  | Ratio |  | 7.53 | 0.007 |
| 1977 | E(ACT)-H/M(TTC)-158 | Leaf length |  |  |  | 3.69 | 0.035 |
|  | E(ACT)-H/M(TTC)-158 |  |  |  | Leaf area | 3.76 | 0.036 |
| 1983 | E(ACT)-H/M(TTC)-166 |  |  | Ratio |  | 6.38 | 0.018 |
| 2005 | E(ACT)-H/M(TTC)-214 | Leaf length |  |  |  | 3.82 | 0.030 |
|  | E(ACT)-H/M(TTC)-214 |  |  | Ratio |  | 5.94 | 0.025 |
| 2006 | E(ACT)-H/M(TTC)-216 |  |  | Ratio |  | 5.20 | 0.044 |
| 2022 | E(ACT)-H/M(TTC)-263 | Leaf length |  |  |  | 3.50 | 0.043 |
|  | E(ACT)-H/M(TTC)-263 |  |  |  | Leaf area | 3.54 | 0.045 |
| 2025 | E(ACT)-H/M(TTC)-271 | Leaf length |  |  |  | 5.32 | 0.005 |
|  | E(ACT)-H/M(TTC)-271 |  | Leaf width |  |  | 4.46 | 0.021 |
|  | E(ACT)-H/M(TTC)-271 |  |  |  | Leaf area | 5.27 | 0.007 |
| 2046 | E(ACT)-H/M(TTC)-328 |  | Leaf width |  |  | 4.86 | 0.014 |
|  | E(ACT)-H/M(TTC)-328 |  |  |  | Leaf area | 3.56 | 0.045 |
| 2047 | E(ACT)-H/M(TTC)-330 | Leaf length |  |  |  | 3.38 | 0.049 |
|  | E(ACT)-H/M(TTC)-330 |  |  |  | Leaf area | 3.48 | 0.049 |
| 2048 | E(ACT)-H/M(TTC)-339 | Leaf length |  |  |  | 3.64 | 0.037 |
|  | E(ACT)-H/M(TTC)-339 |  |  | Ratio |  | 7.10 | 0.010 |
| 2099 | E(ACT)-H/M(TTA)-073 | Leaf length |  |  |  | 7.49 | 0.000 |
|  | E(ACT)-H/M(TTA)-073 |  | Leaf width |  |  | 3.67 | 0.047 |
|  | E(ACT)-H/M(TTA)-073 |  |  | Ratio |  | 8.61 | 0.003 |
|  | E(ACT)-H/M(TTA)-073 |  |  |  | Leaf area | 4.91 | 0.010 |
| 2104 | E(ACT)-H/M(TTA)-085 | Leaf length |  |  |  | 5.98 | 0.002 |
|  | E(ACT)-H/M(TTA)-085 |  | Leaf width |  |  | 5.15 | 0.010 |
|  | E(ACT)-H/M(TTA)-085 |  |  |  | Leaf area | 5.67 | 0.004 |
| 2108 | E(ACT)-H/M(TTA)-094 | Leaf length |  |  |  | 3.88 | 0.028 |
|  | E(ACT)-H/M(TTA)-094 |  | Leaf width |  |  | 5.38 | 0.008 |
|  | E(ACT)-H/M(TTA)-094 |  |  |  | Leaf area | 4.39 | 0.018 |
| 2113 | E(ACT)-H/M(TTA)-101 |  |  | Ratio |  | 5.15 | 0.046 |
| 2117 | E(ACT)-H/M(TTA)-107 | Leaf length |  |  |  | 3.59 | 0.039 |
|  | E(ACT)-H/M(TTA)-107 |  | Leaf width |  |  | 5.08 | 0.011 |
|  | E(ACT)-H/M(TTA)-107 |  |  |  | Leaf area | 3.98 | 0.028 |
| 2122 | E(ACT)-H/M(TTA)-116 | Leaf length |  |  |  | 3.63 | 0.037 |
| 2124 | E(ACT)-H/M(TTA)-120 |  | Leaf width |  |  | 4.20 | 0.028 |
| 2127 | E(ACT)-H/M(TTA)-124 |  |  |  | Leaf area | 3.47 | 0.049 |
| 2135 | E(ACT)-H/M(TTA)-140 | Leaf length |  |  |  | 3.65 | 0.036 |
|  | E(ACT)-H/M(TTA)-140 |  | Leaf width |  |  | 4.11 | 0.030 |
|  | E(ACT)-H/M(TTA)-140 |  |  |  | Leaf area | 4.63 | 0.014 |
| 2137 | E(ACT)-H/M(TTA)-143 |  | Leaf width |  |  | 3.81 | 0.041 |
| 2138 | E(ACT)-H/M(TTA)-146 |  |  | Ratio |  | 7.81 | 0.006 |
| 2139 | E(ACT)-H/M(TTA)-148 | Leaf length |  |  |  | 4.54 | 0.013 |
|  | E(ACT)-H/M(TTA)-148 |  | Leaf width |  |  | 4.57 | 0.019 |
|  | E(ACT)-H/M(TTA)-148 |  |  |  | Leaf area | 4.41 | 0.018 |
| 2140 | E(ACT)-H/M(TTA)-149 | Leaf length |  |  |  | 4.45 | 0.015 |
| 2141 | E(ACT)-H/M(TTA)-150 | Leaf length |  |  |  | 3.81 | 0.031 |
|  | E(ACT)-H/M(TTA)-150 |  |  | Ratio |  | 8.86 | 0.003 |
| 2146 | E(ACT)-H/M(TTA)-159 | Leaf length |  |  |  | 4.82 | 0.010 |
|  | E(ACT)-H/M(TTA)-159 |  |  |  | Leaf area | 3.82 | 0.034 |
| 2151 | E(ACT)-H/M(TTA)-170 |  | Leaf width |  |  | 4.25 | 0.026 |
|  | E(ACT)-H/M(TTA)-170 |  |  |  | Leaf area | 4.32 | 0.019 |
| 2153 | E(ACT)-H/M(TTA)-174 | Leaf length |  |  |  | 4.27 | 0.018 |
|  | E(ACT)-H/M(TTA)-174 |  | Leaf width |  |  | 4.11 | 0.030 |
|  | E(ACT)-H/M(TTA)-174 |  |  |  | Leaf area | 4.09 | 0.025 |
| 2166 | E(ACT)-H/M(TTA)-198 | Leaf length |  |  |  | 5.26 | 0.006 |
|  | E(ACT)-H/M(TTA)-198 |  |  |  | Leaf area | 4.40 | 0.018 |
| 2173 | E(ACT)-H/M(TTA)-212 | Leaf length |  |  |  | 6.36 | 0.002 |
|  | E(ACT)-H/M(TTA)-212 |  | Leaf width |  |  | 6.28 | 0.003 |
|  | E(ACT)-H/M(TTA)-212 |  |  |  | Leaf area | 6.97 | 0.001 |
| 2178 | E(ACT)-H/M(TTA)-226 |  |  | Ratio |  | 5.19 | 0.044 |
| 2190 | E(ACT)-H/M(TTA)-255 |  |  | Ratio |  | 6.92 | 0.012 |
| 2193 | E(ACT)-H/M(TTA)-259 | Leaf length |  |  |  | 3.70 | 0.034 |
| 2230 | E(ACT)-H/M(TTA)-367 | Leaf length |  |  |  | 3.87 | 0.028 |
|  | E(ACT)-H/M(TTA)-367 |  | Leaf width |  |  | 3.99 | 0.034 |
|  | E(ACT)-H/M(TTA)-367 |  |  |  | Leaf area | 4.54 | 0.015 |
| 2234 | E(ACT)-H/M(TTA)-380 |  | Leaf width |  |  | 3.83 | 0.040 |
| 2236 | E(ACT)-H/M(TTA)-382 |  | Leaf width |  |  | 3.65 | 0.049 |
| 2239 | E(ACT)-H/M(TTA)-398 | Leaf length |  |  |  | 4.12 | 0.022 |
|  | E(ACT)-H/M(TTA)-398 |  | Leaf width |  |  | 3.64 | 0.049 |
|  | E(ACT)-H/M(TTA)-398 |  |  |  | Leaf area | 3.46 | 0.049 |
| 2240 | E(ACT)-H/M(TTA)-399 |  | Leaf width |  |  | 3.78 | 0.043 |
| 2242 | E(ACT)-H/M(TTA)-415 |  | Leaf width |  |  | 4.07 | 0.032 |
|  | E(ACT)-H/M(TTA)-415 |  |  |  | Leaf area | 3.46 | 0.049 |
| 2247 | E(ACT)-H/M(TTA)-441 | Leaf length |  |  |  | 3.54 | 0.041 |
|  | E(ACT)-H/M(TTA)-441 |  |  |  | Leaf area | 3.60 | 0.043 |
| 2248 | E(ACT)-H/M(TTA)-445 |  | Leaf width |  |  | 3.90 | 0.038 |
| 2253 | E(ACT)-H/M(TTA)-469 | Leaf length |  |  |  | 5.13 | 0.007 |
|  | E(ACT)-H/M(TTA)-469 |  |  |  | Leaf area | 3.75 | 0.036 |
| 2254 | E(ACT)-H/M(TTA)-470 | Leaf length |  |  |  | 3.99 | 0.025 |
| 2255 | E(ACT)-H/M(TTA)-472 | Leaf length |  |  |  | 4.09 | 0.022 |
|  | E(ACT)-H/M(TTA)-472 |  | Leaf width |  |  | 3.88 | 0.039 |
|  | E(ACT)-H/M(TTA)-472 |  |  |  | Leaf area | 3.67 | 0.039 |
| 2257 | E(ACT)-H/M(TTA)-475 | Leaf length |  |  |  | 4.40 | 0.016 |
|  | E(ACT)-H/M(TTA)-475 |  |  |  | Leaf area | 3.77 | 0.035 |
| 2258 | E(ACT)-H/M(TTA)-478 | Leaf length |  |  |  | 3.46 | 0.045 |
| 2266 | E(AGC)-H/M(TCT)-062 |  |  | Ratio |  | 9.70 | 0.001 |
| 2272 | E(AGC)-H/M(TCT)-073 |  |  | Ratio |  | 5.84 | 0.027 |
| 2275 | E(AGC)-H/M(TCT)-079 |  |  | Ratio |  | 10.19 | 0.001 |
| 2281 | E(AGC)-H/M(TCT)-090 | Leaf length |  |  |  | 4.14 | 0.021 |
|  | E(AGC)-H/M(TCT)-090 |  |  | Ratio |  | 6.19 | 0.021 |
| 2282 | E(AGC)-H/M(TCT)-092 | Leaf length |  |  |  | 3.46 | 0.045 |
| 2283 | E(AGC)-H/M(TCT)-093 |  |  | Ratio |  | 9.67 | 0.000 |
| 2286 | E(AGC)-H/M(TCT)-098 |  |  | Ratio |  | 5.66 | 0.031 |
| 2287 | E(AGC)-H/M(TCT)-100 |  |  | Ratio |  | 5.06 | 0.049 |
| 2291 | E(AGC)-H/M(TCT)-106 |  |  | Ratio |  | 8.42 | 0.004 |
| 2295 | E(AGC)-H/M(TCT)-111 | Leaf length |  |  |  | 3.67 | 0.036 |
|  | E(AGC)-H/M(TCT)-111 |  | Leaf width |  |  | 5.91 | 0.004 |
|  | E(AGC)-H/M(TCT)-111 |  |  |  | Leaf area | 4.99 | 0.009 |
| 2301 | E(AGC)-H/M(TCT)-120 | Leaf length |  |  |  | 3.40 | 0.048 |
| 2302 | E(AGC)-H/M(TCT)-121 |  |  | Ratio |  | 5.15 | 0.046 |
| 2304 | E(AGC)-H/M(TCT)-125 | Leaf length |  |  |  | 3.65 | 0.036 |
|  | E(AGC)-H/M(TCT)-125 |  | Leaf width |  |  | 4.31 | 0.025 |
|  | E(AGC)-H/M(TCT)-125 |  |  |  | Leaf area | 4.19 | 0.022 |
| 2307 | E(AGC)-H/M(TCT)-129 | Leaf length |  |  |  | 4.49 | 0.014 |
|  | E(AGC)-H/M(TCT)-129 |  |  | Ratio |  | 5.79 | 0.029 |
|  | E(AGC)-H/M(TCT)-129 |  |  |  | Leaf area | 3.92 | 0.030 |
| 2309 | E(AGC)-H/M(TCT)-133 |  |  |  | Leaf area | 4.03 | 0.027 |
| 2310 | E(AGC)-H/M(TCT)-135 |  |  | Ratio |  | 5.06 | 0.049 |
| 2317 | E(AGC)-H/M(TCT)-147 | Leaf length |  |  |  | 3.51 | 0.042 |
| 2320 | E(AGC)-H/M(TCT)-153 |  |  | Ratio |  | 5.96 | 0.025 |
| 2323 | E(AGC)-H/M(TCT)-158 |  |  | Ratio |  | 5.32 | 0.040 |
| 2327 | E(AGC)-H/M(TCT)-165 |  |  | Ratio |  | 5.10 | 0.047 |
| 2338 | E(AGC)-H/M(TCT)-187 |  |  | Ratio |  | 5.35 | 0.040 |
| 2358 | E(AGC)-H/M(TCT)-223 |  | Leaf width |  |  | 3.87 | 0.039 |
| 2361 | E(AGC)-H/M(TCT)-228 |  |  | Ratio |  | 6.21 | 0.021 |
| 2364 | E(AGC)-H/M(TCT)-237 | Leaf length |  |  |  | 5.30 | 0.006 |
|  | E(AGC)-H/M(TCT)-237 |  |  | Ratio |  | 5.32 | 0.040 |
|  | E(AGC)-H/M(TCT)-237 |  |  |  | Leaf area | 3.80 | 0.034 |
| 2365 | E(AGC)-H/M(TCT)-240 |  |  | Ratio |  | 5.65 | 0.032 |
| 2368 | E(AGC)-H/M(TCT)-246 | Leaf length |  |  |  | 3.74 | 0.033 |
| 2387 | E(AGC)-H/M(TCT)-287 |  |  | Ratio |  | 8.14 | 0.005 |
| 2390 | E(AGC)-H/M(TCT)-295 |  | Leaf width |  |  | 4.30 | 0.025 |
| 2397 | E(AGC)-H/M(TCT)-326 | Leaf length |  |  |  | 3.45 | 0.045 |
| 2410 | E(AGC)-H/M(TCT)-374 | Leaf length |  |  |  | 3.41 | 0.047 |
|  | E(AGC)-H/M(TCT)-374 |  | Leaf width |  |  | 3.76 | 0.044 |
|  | E(AGC)-H/M(TCT)-374 |  |  |  | Leaf area | 4.21 | 0.022 |
| 2413 | E(AGC)-H/M(TCT)-385 |  | Leaf width |  |  | 4.84 | 0.014 |
|  | E(AGC)-H/M(TCT)-385 |  |  |  | Leaf area | 4.19 | 0.022 |
| 2415 | E(AGC)-H/M(TCT)-388 | Leaf length |  |  |  | 3.82 | 0.030 |
|  | E(AGC)-H/M(TCT)-388 |  | Leaf width |  |  | 4.94 | 0.013 |
|  | E(AGC)-H/M(TCT)-388 |  |  |  | Leaf area | 4.50 | 0.016 |
| 2416 | E(AGC)-H/M(TCT)-392 | Leaf length |  |  |  | 3.76 | 0.032 |
|  | E(AGC)-H/M(TCT)-392 |  | Leaf width |  |  | 4.66 | 0.017 |
|  | E(AGC)-H/M(TCT)-392 |  |  |  | Leaf area | 4.74 | 0.012 |
| 2421 | E(AGC)-H/M(TCT)-409 |  | Leaf width |  |  | 4.69 | 0.017 |
|  | E(AGC)-H/M(TCT)-409 |  |  |  | Leaf area | 3.91 | 0.030 |
| 2423 | E(AGC)-H/M(TCT)-415 |  | Leaf width |  |  | 3.80 | 0.042 |
|  | E(AGC)-H/M(TCT)-415 |  |  |  | Leaf area | 3.48 | 0.049 |
| 2426 | E(AGC)-H/M(TCT)-427 |  | Leaf width |  |  | 3.75 | 0.044 |
|  | E(AGC)-H/M(TCT)-427 |  |  |  | Leaf area | 3.61 | 0.042 |
| 2427 | E(AGC)-H/M(TCT)-428 | Leaf length |  |  |  | 3.90 | 0.028 |
|  | E(AGC)-H/M(TCT)-428 |  | Leaf width |  |  | 4.79 | 0.015 |
|  | E(AGC)-H/M(TCT)-428 |  |  |  | Leaf area | 4.76 | 0.012 |
| 2430 | E(AGC)-H/M(TCT)-446 |  | Leaf width |  |  | 3.81 | 0.041 |
|  | E(AGC)-H/M(TCT)-446 |  |  |  | Leaf area | 3.62 | 0.042 |
| 2432 | E(AGC)-H/M(TCT)-458 |  | Leaf width |  |  | 3.85 | 0.039 |
|  | E(AGC)-H/M(TCT)-458 |  |  |  | Leaf area | 3.73 | 0.037 |
| 2434 | E(AGC)-H/M(TCT)-470 |  | Leaf width |  |  | 3.92 | 0.037 |
|  | E(AGC)-H/M(TCT)-470 |  |  |  | Leaf area | 3.72 | 0.038 |
| 2435 | E(AGC)-H/M(TCT)-474 |  | Leaf width |  |  | 4.09 | 0.031 |
|  | E(AGC)-H/M(TCT)-474 |  |  |  | Leaf area | 3.84 | 0.033 |
| 2441 | E(AGC)-H/M(TCT)-526 |  |  |  | Leaf area | 3.55 | 0.045 |
| 2444 | E(AGC)-H/M(TCT)-557 |  | Leaf width |  |  | 3.94 | 0.036 |
|  | E(AGC)-H/M(TCT)-557 |  |  |  | Leaf area | 3.82 | 0.034 |
| 2445 | E(AGC)-H/M(TCT)-558 |  | Leaf width |  |  | 3.94 | 0.036 |
|  | E(AGC)-H/M(TCT)-558 |  |  |  | Leaf area | 3.82 | 0.034 |
| 2446 | E(AGC)-H/M(TCT)-566 |  | Leaf width |  |  | 4.51 | 0.020 |
|  | E(AGC)-H/M(TCT)-566 |  |  |  | Leaf area | 4.05 | 0.026 |
| 2448 | E(AGC)-H/M(TCT)-574 |  | Leaf width |  |  | 3.94 | 0.036 |
|  | E(AGC)-H/M(TCT)-574 |  |  |  | Leaf area | 3.82 | 0.034 |
| 2465 | E(AGC)-H/M(TCC)-085 | Leaf length |  |  |  | 3.66 | 0.036 |
|  | E(AGC)-H/M(TCC)-085 |  |  |  | Leaf area | 3.89 | 0.031 |
| 2473 | E(AGC)-H/M(TCC)-097 | Leaf length |  |  |  | 4.00 | 0.025 |
|  | E(AGC)-H/M(TCC)-097 |  |  | Ratio |  | 11.78 | 0.000 |
| 2483 | E(AGC)-H/M(TCC)-113 | Leaf length |  |  |  | 4.93 | 0.008 |
|  | E(AGC)-H/M(TCC)-113 |  | Leaf width |  |  | 5.65 | 0.006 |
|  | E(AGC)-H/M(TCC)-113 |  |  |  | Leaf area | 6.07 | 0.003 |
| 2485 | E(AGC)-H/M(TCC)-116 | Leaf length |  |  |  | 4.03 | 0.024 |
| 2491 | E(AGC)-H/M(TCC)-126 | Leaf length |  |  |  | 4.31 | 0.017 |
|  | E(AGC)-H/M(TCC)-126 |  |  | Ratio |  | 5.68 | 0.031 |
| 2492 | E(AGC)-H/M(TCC)-127 |  |  | Ratio |  | 6.25 | 0.020 |
| 2497 | E(AGC)-H/M(TCC)-136 | Leaf length |  |  |  | 3.65 | 0.036 |
|  | E(AGC)-H/M(TCC)-136 |  |  | Ratio |  | 6.99 | 0.011 |
| 2505 | E(AGC)-H/M(TCC)-148 |  |  | Ratio |  | 5.41 | 0.038 |
| 2515 | E(AGC)-H/M(TCC)-164 | Leaf length |  |  |  | 3.61 | 0.038 |
| 2524 | E(AGC)-H/M(TCC)-180 |  |  |  | Leaf area | 3.52 | 0.046 |
| 2530 | E(AGC)-H/M(TCC)-191 | Leaf length |  |  |  | 3.57 | 0.040 |
|  | E(AGC)-H/M(TCC)-191 |  | Leaf width |  |  | 3.66 | 0.048 |
|  | E(AGC)-H/M(TCC)-191 |  |  |  | Leaf area | 4.07 | 0.026 |
| 2541 | E(AGC)-H/M(TCC)-208 |  |  | Ratio |  | 8.39 | 0.004 |
| 2558 | E(AGC)-H/M(TCC)-238 |  |  | Ratio |  | 7.51 | 0.008 |
| 2562 | E(AGC)-H/M(TCC)-247 |  |  | Ratio |  | 5.30 | 0.041 |
| 2563 | E(AGC)-H/M(TCC)-248 |  | Leaf width |  |  | 3.83 | 0.040 |
|  | E(AGC)-H/M(TCC)-248 |  |  |  | Leaf area | 3.78 | 0.035 |
| 2564 | E(AGC)-H/M(TCC)-249 |  |  | Ratio |  | 5.95 | 0.025 |
| 2590 | E(AGC)-H/M(TCC)-317 | Leaf length |  |  |  | 3.50 | 0.043 |
|  | E(AGC)-H/M(TCC)-317 |  | Leaf width |  |  | 3.69 | 0.046 |
|  | E(AGC)-H/M(TCC)-317 |  |  |  | Leaf area | 3.84 | 0.033 |
| 2592 | E(AGC)-H/M(TCC)-321 | Leaf length |  |  |  | 4.51 | 0.014 |
|  | E(AGC)-H/M(TCC)-321 |  |  |  | Leaf area | 4.39 | 0.018 |
| 2611 | E(AGC)-H/M(TCC)-374 |  |  | Ratio |  | 5.85 | 0.027 |
| 2614 | E(AGC)-H/M(TCC)-380 | Leaf length |  |  |  | 3.46 | 0.045 |
| 2618 | E(AGC)-H/M(TCC)-397 |  |  | Ratio |  | 6.14 | 0.022 |
| 2632 | E(AGC)-H/M(TCC)-458 |  | Leaf width |  |  | 4.00 | 0.034 |
| 2636 | E(AGC)-H/M(TCC)-468 |  | Leaf width |  |  | 3.65 | 0.049 |
| 2646 | E(AGG)-H/M(TGA)-066 | Leaf length |  |  |  | 4.19 | 0.020 |
|  | E(AGG)-H/M(TGA)-066 |  | Leaf width |  |  | 3.78 | 0.043 |
| 2651 | E(AGG)-H/M(TGA)-075 | Leaf length |  |  |  | 5.97 | 0.002 |
|  | E(AGG)-H/M(TGA)-075 |  | Leaf width |  |  | 5.15 | 0.010 |
|  | E(AGG)-H/M(TGA)-075 |  |  |  | Leaf area | 5.51 | 0.005 |
| 2652 | E(AGG)-H/M(TGA)-077 |  |  | Ratio |  | 9.67 | 0.001 |
| 2655 | E(AGG)-H/M(TGA)-083 |  | Leaf width |  |  | 4.41 | 0.022 |
| 2657 | E(AGG)-H/M(TGA)-087 | Leaf length |  |  |  | 5.17 | 0.006 |
|  | E(AGG)-H/M(TGA)-087 |  | Leaf width |  |  | 3.64 | 0.049 |
|  | E(AGG)-H/M(TGA)-087 |  |  | Ratio |  | 6.26 | 0.020 |
|  | E(AGG)-H/M(TGA)-087 |  |  |  | Leaf area | 3.99 | 0.028 |
| 2663 | E(AGG)-H/M(TGA)-101 |  |  | Ratio |  | 7.41 | 0.008 |
| 2664 | E(AGG)-H/M(TGA)-103 |  |  | Ratio |  | 5.05 | 0.049 |
| 2671 | E(AGG)-H/M(TGA)-112 |  |  | Ratio |  | 5.74 | 0.029 |
| 2673 | E(AGG)-H/M(TGA)-114 | Leaf length |  |  |  | 5.74 | 0.003 |
|  | E(AGG)-H/M(TGA)-114 |  | Leaf width |  |  | 3.87 | 0.039 |
|  | E(AGG)-H/M(TGA)-114 |  |  |  | Leaf area | 4.51 | 0.016 |
| 2675 | E(AGG)-H/M(TGA)-116 |  | Leaf width |  |  | 3.64 | 0.049 |
|  | E(AGG)-H/M(TGA)-116 |  |  |  | Leaf area | 3.64 | 0.041 |
| 2683 | E(AGG)-H/M(TGA)-128 | Leaf length |  |  |  | 4.33 | 0.017 |
|  | E(AGG)-H/M(TGA)-128 |  |  |  | Leaf area | 4.06 | 0.026 |
| 2685 | E(AGG)-H/M(TGA)-132 | Leaf length |  |  |  | 4.01 | 0.024 |
| 2692 | E(AGG)-H/M(TGA)-143 |  |  | Ratio |  | 6.24 | 0.020 |
| 2694 | E(AGG)-H/M(TGA)-148 | Leaf length |  |  |  | 4.13 | 0.021 |
|  | E(AGG)-H/M(TGA)-148 |  | Leaf width |  |  | 5.40 | 0.008 |
|  | E(AGG)-H/M(TGA)-148 |  |  |  | Leaf area | 4.96 | 0.009 |
| 2696 | E(AGG)-H/M(TGA)-153 | Leaf length |  |  |  | 4.67 | 0.011 |
|  | E(AGG)-H/M(TGA)-153 |  |  | Ratio |  | 8.78 | 0.003 |
| 2700 | E(AGG)-H/M(TGA)-164 |  |  | Ratio |  | 7.74 | 0.006 |
| 2707 | E(AGG)-H/M(TGA)-176 | Leaf length |  |  |  | 3.60 | 0.038 |
| 2723 | E(AGG)-H/M(TGA)-207 |  |  | Ratio |  | 5.68 | 0.031 |
| 2737 | E(AGG)-H/M(TGA)-240 |  | Leaf width |  |  | 4.78 | 0.015 |
|  | E(AGG)-H/M(TGA)-240 |  |  |  | Leaf area | 4.43 | 0.017 |
| 2751 | E(AGG)-H/M(TGA)-270 | Leaf length |  |  |  | 4.97 | 0.008 |
|  | E(AGG)-H/M(TGA)-270 |  | Leaf width |  |  | 4.57 | 0.019 |
|  | E(AGG)-H/M(TGA)-270 |  |  |  | Leaf area | 5.03 | 0.009 |
| 2753 | E(AGG)-H/M(TGA)-276 | Leaf length |  |  |  | 3.61 | 0.038 |
|  | E(AGG)-H/M(TGA)-276 |  |  |  | Leaf area | 3.50 | 0.047 |
| 2754 | E(AGG)-H/M(TGA)-277 |  | Leaf width |  |  | 4.53 | 0.019 |
|  | E(AGG)-H/M(TGA)-277 |  |  |  | Leaf area | 3.57 | 0.044 |
| 2760 | E(AGG)-H/M(TGA)-294 |  | Leaf width |  |  | 4.40 | 0.022 |
| 2763 | E(AGG)-H/M(TGA)-313 |  |  | Ratio |  | 5.23 | 0.043 |
| 2765 | E(AGG)-H/M(TGA)-318 | Leaf length |  |  |  | 3.50 | 0.043 |
|  | E(AGG)-H/M(TGA)-318 |  | Leaf width |  |  | 4.63 | 0.018 |
|  | E(AGG)-H/M(TGA)-318 |  |  |  | Leaf area | 4.37 | 0.018 |
| 2780 | E(AGG)-H/M(TGA)-371 | Leaf length |  |  |  | 4.51 | 0.014 |
|  | E(AGG)-H/M(TGA)-371 |  | Leaf width |  |  | 5.50 | 0.007 |
|  | E(AGG)-H/M(TGA)-371 |  |  |  | Leaf area | 5.30 | 0.006 |
| 2790 | E(AGG)-H/M(TGA)-421 | Leaf length |  |  |  | 3.38 | 0.049 |
|  | E(AGG)-H/M(TGA)-421 |  |  | Ratio |  | 5.17 | 0.045 |
|  | E(AGG)-H/M(TGA)-421 |  |  |  | Leaf area | 3.58 | 0.044 |
| 2797 | E(AGG)-H/M(TGA)-464 |  |  | Ratio |  | 6.78 | 0.013 |
| 2798 | E(AGG)-H/M(TGA)-469 |  | Leaf width |  |  | 3.67 | 0.048 |
| 2807 | E(AGG)-H/M(TGA)-489 |  |  |  | Leaf area | 3.48 | 0.048 |

^a^The number was consistent with the sequential order of reading MSAP bands.

^b^The MSAP-marker was denominated as primer combination and fragment length.

^c^Ratio stood for ratio of leaf length to width.

**Supplementary TABLE 6 | Sequencing of associated candidate markers referenced to the genome of *P. mume* and other species.**

| **No.** | **Name** | **Accession** | **Score** | **Query**  **cover** | **E_value** | **Ident** | **Describtion** |
| --- | --- | --- | --- | --- | --- | --- | --- |
|
| AFLP | | | | | | | |
| 74 | E(AAC)-M(CTG)-371 | Prupe.1G400300.1 | 441 | - | 1.86E-120 | - | *Prunus persica*, (1 of 5) PF00060//PF00497//PF01094-Ligand-gated ion channel (Lig_chan)//Bacterial extracellular solute-binding proteins, family 3 (SBP_bac_3)//Receptor family ligand binding region (ANF_receptor) |
| 140 | E(AAG)-M(CTA)-189 | Prupe.2G119300.1 | 173 | - | 3.50E-42 | - | *Prunus persica* (1 of 2) PTHR23084:SF156-radial spoke head 10 homolog B |
| 156 | E(AAG)-M(CTA)-227 | XM_007222032.2 | 327 | 96% | 4.00E-86 | 96% | PREDICTED: *Prunus persica* probable cinnamyl alcohol dehydrogenase 6 (LOC18790446) |
| 161 | E(AAG)-M(CTA)-236 | - | 254 | - | 2.83E-65 | - | *Prunus persica*, unkonwn function |
| 201 | E(AAG)-M(CTA)-330 | Prupe.1G464500.1 | 344 | - | 1.90E-93 | - | *Prunus persica*, (1 of 1) K19027-zinc finger FYVE domain-containing protein 26 (ZFYVE26) |
| 253 | E(AAG)-M(CTA)-503 | - | 557 | - | 4.39E-156 | - | *Prunus persica*, unkonwn function |
| 305 | E(ACA)-M(CAA)-133 | XM_008235145.2 | 165 | 95% | 3.00E-37 | 95% | PREDICTED: *Prunus mume* LOB domain-containing protein 1-like (LOC103332406), mitochondrion |
| 361 | E(ACA)-M(CAA)-269 | XM_016794139.1 | 416 | 99% | 8.00E-113 | 97% | PREDICTED: *Prunus mume* uncharacterized LOC103330487 (LOC103330487) |
| 363 | E(ACA)-M(CAA)-279 | Prupe.2G066200.1 | 398 | - | 8.00E-110 | - | *Prunus persica*, (1 of 3) K11838-ubiquitin carboxyl-terminal hydrolase 7 (USP7, UBP15) |
| 512 | E(ACA)-M(CAT)-262 | - | 342 | - | 8.20E-92 | - | *Prunus persica*, Serine hydroxymethyltransferase 2 Serine hydroxymethyltransferase, mitochondrial; by homology to *Arabidopsis* gene (Identifier 023254); found in the flagellar proteome [PMID: 15998802] Cre06.g293950.t1.2 g6806.t1 Cre06.g293950.t1.1 Cre06.g293950 Cre06.g293950.t1.2 |
| 525 | E(ACA)-M(CAT)-320 | Prupe.8G099300.1 | 331 | - | 1.10E-89 | - | *Prunus persica*, RmlC-like cupins superfamily protein, AT5G39160.1 |
| 543 | E(ACA)-M(CAT)-393 | Prupe.3G258800.1 | 429 | - | 7.00E-119 | - | *Prunus persica* SF1-calmodulin-binding protein-like protein |
| 622 | E(ACC)-M(CAC)-160 | - | 202 | - | 5.60E-51 | - | *Prunus persica*, unkonwn function |
| 688 | E(ACC)-M(CAG)-129 | - | 54 | - | 2.20E-05 | - | *Prunus persica*, unkonwn function |
| 722 | E(ACC)-M(CAG)-292 | Phvul.004G112000.1 | 46 | - | 9.90E-03 | - | *Phaseolus vulgaris* (1 of 2) PTHR24361//PTHR24361: SF253-Mitogen-activated kinase kinase//subfamily not named |
| 764 | E(ACG)-M(CTA)-93 | - | 90 | - | 1.10E-17 | - | *Prunus persica*, unkonwn function |
| 816 | E(ACG)-M(CTA)-217 | - | 290 | - | 2.20E-77 | 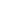- | *Prunus persica*, unkonwn function |
| 832 | E(ACG)-M(CTA)-265 | Prupe.6G147300.1 | 86 | - | 1.10E-10 | - | *Prunus persica* (1 of 4) K11835-ubiquitin carboxyl-terminal hydrolase 4/11/15 [EC:3.4.19.12] (USP4_11_15, UBP12) |
| 845 | E(ACG)-M(CTA)-309 | XM_008220463.2 | 622 | 100% | 2.00E-94 | 99% | PREDICTED: *Prunus mume* farnesyl pyrophosphate synthase 1-like (LOC103318981) |
| 928 | E(ACG)-M(CTT)-130 | - | 117 | - | 6.00E-24 | - | *Prunus persica*, unkonwn function |
| 950 | E(ACG)-M(CTT)-190 | XM_008232574.2 | 84.2 | 30% | 1.00E-12 | 96% | PREDICTED: *Prunus mume* cystathionine gamma-synthase 1, chloroplastic-like (LOC103330036) |
| 985 | E(ACG)-M(CTT)-291 | XM_008220308.2 | 431 | 97% | 3.00E-117 | 97% | PREDICTED: *Prunus mume* coiled-coil domain-containing protein 22 (LOC103318866) |
| 1112 | E(ACT)-M(CAC)-142 | XM_016794170.1 | 115 | 71% | 3.00E-22 | 90% | PREDICTED: *Prunus mume* uncharacterized mitochondrial protein AtMg00810-like (LOC107881096) |
| 1113 | E(ACT)-M(CAC)-144 | Prupe.5G058100.1 | 117 | - | 9.70E-23 | - | PREDICTED: *Prunus persica* (1 of 1) K09493-T-complex protein 1 subunit alpha (CCT1, TCP1) |
| 1125 | E(ACT)-M(CAC)-191 | - | 229 | - | 5.30E-59 | 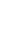- | *Prunus persica*, unkonwn function |
| 1128 | E(ACT)-M(CAC)-208 | KT458993.1 | 294 | 89% | 4.00E-76 | 98% | *Prunus virginiana* voucher Ahrendsen_4 MatR gene, mitochondrial |
| 1224 | E(ACT)-M(CAT)-158 | XM_008227766.2 | 220 | 94% | 5.00E-54 | 98% | PREDICTED: *Prunus mume* uncharacterized LOC103325578 (LOC103325578), mRNA |
| 1258 | E(ACT)-M(CAT)-320 | XM_016795276.1 | 475 | 95% | 1.00E-130 | 97% | PREDICTED: *Prunus mume* protein gigantean (LOC103336221) |
| 1318 | E(AGC)-M(CTC)-162 | XM_008231414.2 | 180 | 99% | 1.00E-41 | 91% | PREDICTED: *Prunus mume* UDP-glycosyltransferase 71A16-like (LOC103328986) |
| 1325 | E(AGC)-M(CTC)-184 | XM_008231519.1 | 276 | 96% | 2.00E-70 | 99% | PREDICTED: *Prunus mume* 7-deoxyloganetin glucosyltransferase-like (LOC103329090) |
| 1475 | E(AGC)-M(CTG)-216 | XM_007215944.2 | 182 | 56% | 3.00E-42 | 96% | PREDICTED: *Prunus persica* transcription termination factor mTERF5, chloroplastic (LOC18782766) |
| 1576 | E(AGG)-M(CAA)-209 | XM_016795232.1 | 215 | 81% | 3.00E-52 | 92% | PREDICTED: *Prunus mume* TMV resistance protein N-like (LOC103336259) |
| 1699 | E(AGG)-M(CAG)-154 | Prupe.2G061100.2 | 156 | - | 2.00E-37 | - | PREDICTED: *Prunus persica* mannose-6-phosphate 6-reductase |
| 1723 | E(AGG)-M(CAG)-257 | XM_021949932.1 | 216 | 50% | 1.00E-21 | 98% | PREDICTED: *Prunus avium* anaphase-promoting complex subunit 1 (LOC110749754) |
| 1783 | E(AGG)-M(CTC)-113 | Prupe.6G048800.1 | 102 | - | 3.80E-20 | - | *Prunus persica*, (1 of 2) Pther11668//Pther11668:SF267-serine/threonine protein phosphatase//Subfamily not named |
| 1802 | E(AGG)-M(CTC)-176 | Prupe.5G122000.1 | 167 | - | 1.30E-40 | - | PREDICTED: *Prunus persica* SF281-SGNH hydrolase-type esterase superfamily protein |
| 1834 | E(AGG)-M(CTC)-317 | XM_008242511.2 | 313 | 65% | 1.00E-81 | 96% | PREDICTED: *Prunus mume* probable methyltransferase PMT7 (LOC103339240), At5g04060 |
| 1840 | E(AGG)-M(CTC)-366 | XM_008248157.2 | 566 | 100% | 8.00E-158 | 97% | PREDICTED: *Prunus mume* vignain-like (LOC103344563) |
| MSAP | | | | | | | |
| 115 | E(AAC)-H/M(TCG)-266 | XM_016793037.1 | 119 | 27% | 4.00E-23 | 99% | PREDICTED: *Prunus mume* DNA polymerase zeta processivity subunit |
| 448 | E(AAG)-H/M(TTG)-249 | XR_001677955.1 | 94 | 24% | 3.00E-15 | 96% | PREDICTED: *Prunus mume* uncharacterized LOC103331672 (LOC103331672) |
| 473 | E(AAG)-H/M(TTG)-315 | - | 131 | - | 3.19E-28 | - | *Prunus persica*, unkonwn function |
| 534 | E(AAG)-H/M(TTG)-460 | XM_008238729.1 | 702 | 98% | 0.00 | 96% | PREDICTED: *Prunus mume* SUMO-activating enzyme subunit 2 (LOC103335702) |
| 662 | E(AAG)-H/M(TTA)-247 | XM_008231070.1 | 219 | 100% | 4.00E-53 | 84% | PREDICTED: *Prunus mume* uncharacterized LOC103328662 (LOC103328662) |
| 864 | E(ACA)-H/M(TCG)-319 | XM_021969597.1 | 100 | 21% | 2.00E-17 | 95% | PREDICTED: *Prunus avium* probable 2-oxoglutarate-dependent dioxygenase AOP1 (LOC110766294) |
| 879 | E(ACA)-H/M(TCG)-369 | AB537563.1 | 169 | 52% | 4.00E-38 | 84% | *Prunus persica* S3-RNase gene for S-ribonuclease 3 |
| 1042 | E(ACA)-H/M(TTG)-270 | Prupe.6G263700.1 | 358 | - | 7.60E-96 | - | *Prunus persica* (1 of 1) PTHR11886//PTHR11886:SF46-dynein light chain// Subfamily not named |
| 1080 | E(ACA)-H/M(TTG)-365 | XM_008240989.1 | 542 | 100% | 2.00E-150 | 95% | PREDICTED: *Prunus mume* zinc finger MYM-type protein 1-like (LOC103337826) |
| 1221 | E(ACC)-H/M(TCC)-210 | XM_020563029.1 | 167 | 72% | 1.00E-37 | 90% | PREDICTED: *Prunus persica* zinc finger MYM-type protein At5g63440 (LOC18780855) |
| 1232 | E(ACC)-H/M(TCC)-231 | FM253563.1 | 189 | 100% | 3.00E-44 | 85% | *Prunus cerasifera* BAC insert containing Ma gene, resistant Ma1 allele |
| 1234 | E(ACC)-H/M(TCC)-239 | XM_008235894.2 | 198 | 77% | 5.00E-47 | 89% | PREDICTED: *Prunus mume* uncharacterized LOC103333102 (LOC103333102) |
| 1438 | E(ACC)-H/M(TTC)-279 | - | 429 | - | 6.80E-119 | 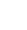- | *Prunus persica*, unkonwn function |
| 1679 | E(ACG)-H/M(TCT)-377 | XM_008241125.2 | 599 | 96% | 1.00E-167 | 98% | PREDICTED: *Prunus mume* quinolinate synthase, chloroplastic (LOC103337950) |
| 1680 | E(ACG)-H/M(TCT)-385 | XM_020562477.1 | 165 | 51% | 5.00E-37 | 83% | PREDICTED: *Prunus persica* uncharacterized LOC109948762 (LOC109948762) |
| 1837 | E(ACG)-H/M(TGT)-278 | XM_008226873.1 | 484 | 100% | 4.00E-133 | 98% | PREDICTED: *Prunus mum*e callose synthase 11 (LOC103324766) |
| 1954 | E(ACT)-H/M(TTC)-119 | XM_008236159.2 | 150 | 100% | 1.00E-32 | 96% | PREDICTED: *Prunus mume* pentatricopeptide repeat-containing protein At4g25270, chloroplastic (LOC103333337) |
| 2025 | E(ACT)-H/M(TTC)-271 | XM_008243558.2 | 466 | 100% | 1.00E-127 | 98% | PREDICTED: *Prunus mume* GATA transcription factor 5-like (LOC103340171), mRNA |
| 2046 | E(ACT)-H/M(TTA)-328 | KR534606.1 | 442 | 85% | 2.00E-120 | 98% | *Malus hupehensis* var. mengshanensis mitochondrion, complete genome |
| 2230 | E(ACT)-H/M(TTA)-367 | XM_008224044.2 | 508 | 87% | 2.00E-140 | 97% | PREDICTED: *Prunus mume* protein NRT1/PTR Family 6.1 (LOC103322156) |
| 2240 | E(ACT)-H/M(TTA)-399 | XM_008234289.1 | 449 | 100% | 1.00E-122 | 97% | PREDICTED: *Prunus mume* uncharacterized LOC103331645 (LOC103331645) |
| 2242 | E(ACT)-H/M(TTA)-415 | XM_008239092.2 | 435 | 62% | 4.00E-118 | 99% | PREDICTED: *Prunus mume* uncharacterized LOC103336056 (LOC103336056) |
| 2258 | E(ACT)-H/M(TTA)-478 | HQ336405.1 | 776 | 94% | 0.00 | 99% | *Prunus persica* chloroplast, mRNA |
| 2364 | E(AGC)-H/M(TCT)-237 | XM_016793577.1 | 172 | 45% | 3.00E-39 | 99% | PREDICTED: *Prunus mume* uncharacterized LOC103327882 (LOC103327882) |
| 2390 | E(AGC)-H/M(TCT)-295 | - | 344 | - | 2.70E-92 | 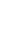- | *Prunus persica*, unkonwn function |
| 2491 | E(AGC)-H/M(TCC)-126 | XM_008226026.2 | 180 | 100% | 2.00E-41 | 99% | PREDICTED: *Prunus mume* protein TIFY 4B-like (LOC103323992) |
| 2618 | E(AGC)-H/M(TCC)-397 | XM_008232237.2 | 366 | 62% | 1.00E-97 | 95% | PREDICTED: *Prunus mume* cystinosin homolog (LOC103329736) |
| 2632 | E(AGC)-H/M(TCC)-458 | XM_008234977.2 | 159 | 32% | 2.00E-35 | 88% | PREDICTED: *Prunus mume* BTB/POZ domain-containing protein At4g08455-like (LOC103332251) |
| 2751 | E(AGG)-H/M(TGA)-270 | XM_007205704.2 | 351 | 97% | 4.00E-93 | 93% | PREDICTED: *Prunus persica* THO complex subunit 4A (LOC18773387) |
| 2760 | E(AGG)-H/M(TGA)-294 | MDP0000266650 | 55.4 | - | 1.90E-05 | - | *Malus domestica* (1 of 1) PTHR21631:SF12-phosphoenolpyruvate carboxylase-like peotein |

**Supplementary DATA 1 | The partial sequences of candidate markers**

**AFLP marker**

>74E(AAC)-M(CTG)-371

GAATTCAACATATATATATACACTAACAATTGAGGTTCCTCTTTTTTTTTTTCTTTTCTTGTACAAGCTAAAGCAATATGAACGTCCCAGTCCCATTGATATAGAAAATCAATTTAGGGATGGAACAAATTATTGAACCAAACTCAAACGTTGACAATTTGACATTATAAAGTGTTGTCCTGTACATAAACAAGATTCCTCGTTACTCGATTCCATCAGTTGCAGAGCAGAACTTTGAAGAATTTCAAACATGGTCAAGAATGCTAGCTGCAACCCTGTTTTTTGTCTTTGCTTCTTGTTCTTACTGTTTTGGTCTTTTCTTGGCCACGGCACAGAACACCCCAGTTAA

>140E(AAG)-M(CTA)-189

GAATTCAAGTGCGTCAAGCATGGCCTTGGTCACTACCATTTCAGGTAAATTTGGCAATGGAGTTTGTGGCATTGTTTATGTTATGATTTTTTGGTTATTTTGATCATGGTTATTTCCACTTCCAAGCACACAAAATGCAACTTATCCTGTATCTCCGGTTGCTAGTTAA

>156E(AAG)-M(CTA)-227

GAATTCAAGGTAGGAGATAGAAAGTGGGGGTGGGATGCTTGGCGACATGTTTGGAGTGTGACTTCTGTAAAGACTCCCAAGAGAACTACTGCGATCAGGTCCAGTTACCTACAATGGCATCTTCTGGGATGGTAGTATTACCTACGGTGGTTACTCCAAAATGCTTGTCGCAGATCATAGGTATGGTGCACAACCAGCGAACTAGTTAA

>161E(AAG)-M(CTA)-236

GAATTCAAGTTGATCACCAAGTAACCGGATGACTAAGTCCAACTCTTTTATTATATTAGGACTCCAATGCTAAATAGAATATCTTACATCACATCCGTATAAGCTTCAGAATACTTGTTCTAATTGATCTTGGTATCTGTCTCAGAGTCCTAGCCAAGTTCAATGAGTGTGTGACCTAAAAACGCTTACATTGGCTTCATAAGTCCATAGTTAA

>201E(AAG)-M(CTA)-330

GAATTCAAGTAAAAGAAAATAAAAATACAATAATGATGAACCTTCATGCAATAACAGGACATTTATAAGGGGATCAAAACTTCCCCTTACAATGTATACTGCAAACAAATTAAACAATCTAAGTGAATAAATAGTGAGAAAAAAAAGATGGAACACTGATCAATATGGTTCGTCCTGAAATTTTAGGTAATAAAACCATGTTGTTCCTTTTTCCAATAACTATTTTGATGAAGTCTTATTGCGGGGTGGTAGACTGTCCGCCTTGGAGAAAACGATCTTTATCTTTTATAACAGTGGATTAGTTAA

>253E(AAG)-M(CTA)-503

GAATTCAAGGCAAATGCCGAGTTCATGTGGGCCGAGTCGCCACTGCTAACTTCTAAGACGCGTTCACGGTTCAGATCATTTCGCAATCAGGAAAAGTCAAAGCAGGAAACTCTGGAGGAATAAAATTTGCTATTTACAAACGACGGAAACAAAAAACTGAACATTTGCACGTTAAAAAAGTCTATTCACACGCAAGGAAATAGTTGCAGAGGATAATACTATTAGTAAGAGAAGACTCTTCTGTGGACGGTCTTATGCAAAGAAACAATCCTGCCCTTATTATAGAAACAAACGGTTCTATAATATTGTAAAAACTAAGGGTCTTTTGGTAATTTAGTTCGTCCTCACTTTCAAGCCATTTACCTTCTTGTGTAACTTGTCATCACACATCTCAACATAAAAAATTTCTCATCACGCATAAAAAAGTATTGTAGGATCGGATAAAAAATAAAAGCATTGTAGATGTTTCTAATTCCATAGTTAA

>305E(ACA)-M(CAA)-133

GAATTCACAACTTCAACTCCCATCTGCTTCTTCCCCTCGCACTTTCAATATCCCAGCTATCTTCTCCGACTGCCTCATTTCCCTCTCCTAATAATCCTCTCAGTTGTTAA

>361E(ACA)-M(CAA)-269

GAATTCACATTCGATTCAGACTGTTTGGAGGCATTTTTTATTGAAGAAAAAGCTCACCACTGCACCAGTCATTATGGCACCTGATTGGGAATTACCATAATCGAGATTCTGTGTGATGCCAGTGATTATGCTATTGGAGCTGTCTTGGGTCAAAGGAAGAATAAATTACTACATGTAATTCACTATGCAAGTCGTACATTGAATGATGCTCAACTGAACTATGCCACTACGGAAAAGGAATTGTTAA

>363E(ACA)-M(CAA)-279

GAATTCACATTTATTACAACGTTGCAATTGCAGGGATATGAATTTGGGATCTCCTACCCTGATACTATGTAAAGTTAGTCAGTTTCACATCTTTCCCAAAATCTATCTGTTATGACGCAAGTGACAATTATTTTATATTCTAAGCGATTTATTATTAGAATTTTGAAATCTTATTTTGATAATCCCTGTGGTAAAGGCTTGGTGAGAGGTTGAAGTATCAGAATGAATGTTCATATAAGTGCTCAAATAGTTTTGTTAA

>512E(ACA)-M(CAT)-262

GAATTCACAATGAAAAACTAAAGAATCCCATGCAGAACAATGTCAAAATCATTTACATAAATGTAGAAAATTTATATAAAAAGAACACTAGATTCAAACAACAAACACAAGTGACTCCTAATACATCTTGAGTACATATTGAATGATATAGTGACTAAGTTATTTGATTTCATACTTGGATGAATTAGACTATTGGATTTTTAATTTTACAAGGATAATTTGGTGTATTTGTTCAAATGTTAA

>525E(ACA)-M(CAT)-320

GAATTCACACAAACAAGTTTTCAAATTTTAGAAGATTCAAAAACAGTTTTCAAGTTGCATACCAAACTACTTTTTCAATCTTCTAAAAATGGATTAGAGAACTCTTTATTTTTCAAGAAAAATCACAGTTTTGTAAGTTTCCTACTAAATAATATACCAAACATGCCTTAACTATCTAAGGATGTTTCGTTCAAATATTTGTATCTATCGATTGATTCAAGTAAAGTTCAAGAGCACCCATTGATATTTGACTAAAGAGTTGTTGGAAATTCAGCCTCCACCTGATTCTCATGTTAA

>543E(ACA)-M(CAT)-393

GAATTCACAAACTGTCTGATGCTGTGGTCTAAACCTTCAAAATTTGCACAATCAACTCGAATGTCTAACTTTGGTGCCCCATCTGGAATAATTTCAAAATGAAGAGAGCATGGTTATTTGATTCCATTACTTATTTTGGTGTCGTGTGTGAGTGTTTTTTACTTTTACATGACGTGTTTTCCATAAGTTTGAAAATGTTTTGGTCTTATCTTTTTAGTTGAAATAAGTATTGCCACTATGATTAGTAGGAGCACAACTAGGAGTATGTGGTTAGGTTTTAGCAGGATGTTTCCCACACCAATATAGTTGCCGGGGTTAGTGGTTCCTTGTTTTTGGATCATTTAGTTGTATGCAATGGCCTATTTTATGTTAA

>622E(ACC)-M(CAC)-160

GAATTCACCATCAACGCTTGTTATATCTTGATAATTTAACTGAAAGGTCAATAATGTTAGCTCTCCTGTATGAGCTAACTGTTTGGCTAATAGCTGACATGGCAAAGTTGCTGATGTGTCAAGACTGTTGTGTTAA

>688E(ACC)-M(CAG)-129

GAATTCACCTTTTGCTGTATTTAACGTTCCCACGCGAAGGCAGGTTTGGACCAGTGTGATCAAAGGGCCGCACTCAATTGTTTACTTATTTTTTGTACTATGCTGTTAA

>722E(ACC)-M(CAG)-292

GAATTCACCAAGGTATGCTTTTTCCCTTGTTTTCTCTTGAATTTATTTGGGAAATTTGCTGGTTCCTTTCATCCATGAATGTTGCCCTTTTGTTATGGCTGTGATACACCAAACCCTCTCTTGACCTATTTTGTTATTGCAGCAACGTGGGTGCTTACAATGTGGGTGCTAGCAAAAGAAGGATTTGCATCCATCTTCCCAGGTTCTCTTTTCTTTGGTTTGCTCGAATTGCTGGAAAGGTACCAAATTTTGTGAGAAAAAAGTGTTAA

>764E(ACG)-M(CTA)-93

GAATTCACGTAAAAGCTTGGAAAACAAAAACTATAATATACTTCATTCATGAATAATCTTTCAGAATAGTTAA

>816E(ACG)-M(CTA)-217

GAATTCACGTTTGTATCGATTTTCTTCTGGCTGATCTTATAGCTGACAGCAAATGAATATACTCCACATACTCTGCTATTCATTTGTGGACCAATTCTCCAGTTGCACTACTAGTTCGGGACAAAACAAGCCAGACAAGACAAGACAAGTCATCCAAATTCCATTTTCTTTTTGCTTGCTCTCACGTTGCTATAGTTAA

>832E(ACG)-M(CTA)-265

GAATTCACGTACATCTAATCTACTAAGTCTTACCAAAACATATAAAAGGGACGTAATTTATGTATAACGAAGAACCATTTATTATTTTTCTAGCTTTACCCATAGCCACTGTGTTTGTGTTGAGAGTATAATCCCATGTCGGAAATAGAAGACTTGCCATAGTAGTTGGTAATAAACCAATTTGGACTTCTCCGCTCTTACCAATTCGTTTTGAGTTGGATGCTTATATTCTAAAAAATAGTTAA

>845E(ACG)-M(CTA)-309

GAATTCACGGACGTTTCTCGTCAATGGGTTGATCGGATGCTGGACTACAATGTGCCAGGAGGAAAGCTGAATCGAGGTTTATCCCTTCAAGCATATATTTCTCGTTTCTTGATGATATCATGGATAGCTCTCATACACGTAGGGGTCAGCCCTGCTGGTTCAGACTACCAAAGATTGGCATGATTGCTGTGAATGATGGCATACTACTGCGCAACCATATCCCGAGAGTTCTCAAAAAGCACTTTAGGGGAAGGCCCTACTATGTGGATCTGCTAGATTTAGTTAA

>928E(ACG)-M(CTT)-130

GAATTCACGTTTGCCTTAGTTAGTCAGGTTTCTTTGTTTTCTGTTTTTCCAGGATTCTAAATAGTTATAACTTGGAACCACGTTACCATTCTCTGTGGGAATTGAAGTTAA

>950E(ACG)-M(CTT)-190

GAATTCACGAGCATCGAGGATGGCCAAAATTTTAGAGGCACATCCTAAGGTATAAATTTCGTAAGTTTTTCATAACAGTAAATAGGTAATATGTGTTAGAAATGATTTTTCCCCAATAATGAGAGGTACTCAATAGAACCAACGTTTCTAATATCAGTGAGAAGTTAA

>985E(ACG)-M(CTT)-291

GAATTCACGAAGGGCGGACAGAATAGTGTATTAGGCAGGAAGCATTTGACTGAAAAACTGCAAAATCAGGAGAAACTGCTCATGGAGAAGGTTACTGTCAAAGGCCTCAGAACTAAAGCACCTTGAAGAAGAGTTGGAATTATAAAAAGAAGCCACAGATATGGCATTTGACATCCATCAGCCCGTTGAATTTTACCTTGCAAAACTTATATGAGCAAGTGGATGTTCGAAAGCATCACCTTGGAGAATTGGAAAGTCAGAGAAGTTAA

>1112E(ACT)-M(CAC)-142

GAATTCACTGCCCTTATCAAATAGGGCATCTGGGGTCCTCCTTCTTCCACACATAAACATTGTTGGGTGAAAATGGGTATTCAAGGTAAAACGTCGATCCGATGATACTGTCGAGTGTTAA

>1113E(ACT)-M(CAC)-144

GAATTCACTTGATTGAAGAAAGTTCCAAAATCCAAAATGACAACCTAACCCAGAAGAGATTTCAAGAAATGGCATCAGCCCCCATACGTGGTCTTAACGACTATGGGTTACACAAAGTGTTAA

>1125E(ACT)-M(CAC)-191

GAATTCACTAATGCCTCTTGCTAATTACTCAAGGTCGGACAGAATCCTAGCAGCACATATGCACTCGAAATGGGGGTTTTTGTTGTTGGAAATGAAAAGAAGAAAGTGAACTGAAAGTGCTCGAAAGTAAACACTGGACAAATTGTGAAACAATAATTGAGAGGTGTTAA

>1128E(ACT)-M(CAC)-208

GAATTCACTCTTTACGATCCCGAGTTTCCTAGACACATCGCACTTCCGCTCGGGTCGAGGCCGCCACTCGGCCCTAAGACGGATCAAAGAAGAGTGGGGAACCTCTCGCTGGATTTTGGATTCGACATCAGGAAGTGTTTTCACACCATCGACCGACATCGACTCATCCCAATCTTTAGGGTGTTAA

>1224E(ACT)-M(CAT)-158

GAATTCACTCGATTCCAAAGCCAAGCAACACCCAAGCTGACAAGGAACAGAAGTAGATGGGGATATGTGCATCTATTACCGCCATGAACCCTTTTTGCCTTCAGGATATCTGTATATTTCTTCAGAACGATGTTAA

>1258E(ACT)-M(CAT)-320

GAATTCACTGGCTGCTACTCTGAAAGATGGATTGATCGTCTGCAATTCTCCTCATTGTTTGGGCCGCCCCCACAAGATGCCCTGCGACGGAAGGCTCAACCGGCTTGCCTATGTGGACTACTTTGGTCAGTTTACATCAGAACAGTTTCCTGAGGACATTGCCGAGCTGATCCGTAACCTTATCCATCGGAGTTGAAGCGTCTCTTTGATGATGTCCTGGCTATGTTTGTCCTTCATCACCCTAAGCATGGGCATGCTGTTATCCTTCCAATTATTTCATGTATCATTGATGTTAA

>1318E(AGC)-M(CTC)-162

GAATTCAGCGCGCTGGAGAGTCTGTGGTGCATGGCTGTGCCGGTTGCCACATGGCCAATGTACGCGGAGCAACAACTAAATGCCTTTCAACTTGTGAGGGATTTGAAGGTTAGGCAGTGGGAAAATAAGATTGAGTTAA

>1325E(AGC)-M(CTC)-184

GAATTCAGCCATTGAGAGTTTGTGTGCAGGAGTGCCTATGCTTTGTTGGCCATTCTTTGCAGACCAACAAACAAACTGTTACTATGTTTGCAATGAATGGGGAGTTGGCTTGGAGATCAACAATGATGTCAAGAGAGACCAAGTAGAGAAGCTTGAGTTAA

>1475E(AGC)-M(CTG)-216

GAATTCAGCATATTACTGCTTCTCTCTGGACCTAGCTTACATATTTATTGTAAGCTCTAAATTGTTTATATCAAATCTTCTTGCAGGACGAGAGCTAACACCTCTTGAGATTAGGGAGGCTCTAATCCCTTACCTCGAATCCCTTCTTGATGAGCATGGAGTTTTTCTGGTAGATTTGGTGGAAAAGCTCAGTTAA

>1576E(AGG)-M(CAA)-209

GAATTCAGGTTTTACTTGAACAAAACAGAAATGTGGACGAAGCAAGCCAACAAGTTTAGCTTAGTGTTACCTCAACACCGAAAAATTTACAGAGCCAGGAGAATTTTAAGACCAGCAAGCCAACCAGAACCTCACAAAATATACAACTTCTCAAGATCAGCCATGAAACCGACAAGAAGAAAATTGTTAA

>1699E(AGG)-M(CAG)-154

GAATTCAGGTCATGGACATGTTCCCGTAGAGGCCTGTAAAGACAGTCTTGAGAAGAAGCTTCGGTTGGATTATCTTGATCTATACCTTGTTCAACTTTCCTGTTGCCAAAACCAAGCATACCGGCTGTTAA

>1723E(AGG)-M(CAG)-257

GAATTCAGGCCAAGATTTGATTTCTTGTATATTCCTCACATTTGGATCCAGATTACCCTTCGTAGAGAAACATTATGTAAAACGCCTCGCCTTACAGGAGGTATAAACACAACATGCTAACAAAGAAAATCTGAACATACAGTTGCATTTTGCTGGGCTAGGCAACCGACCTGCTAAAACAAGCTTTGGGACTACAAAGGCCTAGAGAATGTGGAGTTGGGCGAAGAATCTGTTAA

>1783E(AGG)-M(CTC)-113

GAATTCAGGTTCAATGCATACAGATTAGTAACAAATAGTGACAACCCAACTGCAAAAATGTAAATTCAAATGCAAAGCCAGCAAGAGTTAA

>1802E(AGG)-M(CTC)-176

GAATTCAGGATAAAAGCTAAATTTTGCATGTTGAGAATCTGTGGCGTGTTTCTTATCAATGGCATGTGTGTTTGAGAAACTGGCTTAGTTATGGTAAACTTTCTCTCAGGGTTCTTTTTTTTTTGTGAAATTTTCTATCAGCACTTCGAGTTAA

>1834E(AGG)-M(CTC)-317

GAATTCAGGAGCAACCCAATTGAGGATTTCAGGAAATGGGGGGTGGGAATGTGGTGTTTTCAGCGTTTAGTAATTCCAATCGTGGCAGATGATAGTGGTTCTGCTATTGATGGTCGGGTGCTTCTATGCCGGAACTCTCTTTGGAAATAAGCTCCAATTTATGCATCTCAACTTAGCATCAGGTTCTTCATCTCGTTTGTTCCTCTCTCTCTCTCTCTGAGCTTGTTTTTCAGACCAATGAAGGTTTTATGCATGCATTTTAGAAACATTTTTTTTATTTTGGAAAAAGAGTTAA

>1840E(AGG)-M(CTC)-366

GAATTCAGGGGTGTTGGCATCAATGGCAACGGAAACCGGCTGGTAGGCAACAGCCTTCAACAGAGCATTTTCGTCATTTTCGGGCACATTTTCAAAGTCGGTTATGGTTATGGCACCAGATGCATCAGTCTTGTTGTAATCATCAGCCTTGCAACTGCCTCCATCTACACCTGGTAGGTGTAATCAGCATGGCAGTTGCCAGGCTCCTGTTGCGCTCGATCATGTATTGGAAGGCATAACCAAAGTGACCACCACGGCAACCATCATTGTCCAGACCGACGTTGCAATCAACAAGTTCTTGCTCCGATAGCGTGAGTATAAGTTCCCTGTTTTGAGTGTGTTAA

**MSAP marker**

>115E(AAC)-H/M(TCG)-266

GAATTCAACAGAATCCGAGCTGTCTCACCTGCACCATTTTCAATTTGAATTATTCTCATCTAATAACCAAAACATACCAGAAGTAAAGAGGCTTTGAAACCAAAAAATAAGGAGAGATTATCTTTGCAAACTGTGTTGAATAATGTATAGCCGGACGGCTATATAAAAAAAAAATTTATTTATAGAGTTAGCCGATCGGCTATGCGTTTGGAATATTCTGAATACGGAGTGATAGCCGACCGG

>448E(AAG)-H/M(TTG)-249

GAATTCAAGACCTTTGTTCTTGCAATTATCACATCTACTGTTCATGGCATTTATACTACATTTGTACATGCACTTTTATTACAGATCATGAAAGCTGAATTTAACAACCTCTATCTACAGACATGCAACTTGGAAAGAAGTGTCCAGTATATGCCATACAAAACTTACTTCAAATTTGAAAGTTGGTGAAAGGTAATACAGAGTCATCATCTTCACCAACCGG

>473E(AAG)-H/M(TTG)-315

GAATTCAAGTGAGCTTCTGGTTGATTTTGAGCTTTGGTTGCTTCAGTGGGCTCAATGAGCTTTGGTTGCTTCAGTGTTTTGAAGGTTTTTTTTGAGAGTGTTCAAAGTGATCATTTTGAGTTTTTGTTTTTGATTCCGATTTTTTGGTTGTCGTATTGATTTTTTGGTTGTTTTTTACCCTGTTCACCCCTTTCTTGTATTTATAGTAAACTGTTATATACTAGGACTTTAATATAATATATAATCTTCTCTCCATTAATGTACCGGGAAACAATCCAACCGG

>534E(AAG)-H/M(TTG)-460

GAATTCAAGCCTAATGATCTCAGTCAACAGAATGGAAGTATGGAAAAAAATGGTGCAGCCAATGATATATCTTCAGTATCTGCCATGGCATCTCTAGGAATGAAGAAAATCCACGGATGTATGGAGCCTTAGGAAAATTCCAGAGTCTTTCTTGAGGCTTTGAAATTATTTTTCCTGAAAAGGGAAAAGGAGATTGGAAATCTGACTTTTGATAAAGATGATCAGCTAGGGCAGTGGAATTTGTTACTGCTGCAGCCAATATAAGGGCCGCTTCTTTTGGAATCCCATTGCATAGCCTTTTTGAAGCTAAAGGCATTGCTGGTAATATTGTACATGCTGTAGCAACAACGAATGCTATTATTGTTGATTGTTATTGAGGCAATCAAGGTGCTGAAAAATGATACAAAGAATTATAGGATGACTTATTGTCAACCGG

>662E(AAG)-H/M(TTA)-247

GAATTCAAGAGAATCTCCACATCCATGGTTCTGGATGCACTTTTCTCCAAAAGCTGACTGATCAAGCAGCCGGTATAGATGTCCCAAGAACATGCCGGGGCTAAGGAAAATCGTATCACCTTGAGCTATGGCCAAGGCCAAGAGGAACACACACTCATGCAAGCAGTCTTGAAAAGTCACAAAGCACGAACAGCCAAGCCACAGTGCTATAAACAGCTAACCGG

>864E(ACA)-H/M(TCG)-319

GAATTCACAGAACTACTCTTTTGCCTAATGGCCATGACCTCTTTTTGTAATAATTTTTATTTATTTTTAGCTTATTTTGAGCCTGTTTAATTCAACCTATATATAACATGGTTTTATATATGCATACCCCATTACATTTTATCCTCATGTTTATTGCTGAAGCACTTGTTGGTTGTTCTGTTTCCAACAGCAACACTATACTTACTATGATGAGGCAGCTGGATGAGCTGAAGGACATGTATTGAAATGATGAGCGGCGTAGCCGATCGGCTATAATGGTATTTTTCCCGACCGG

>879E(ACA)-H/M(TCG)-369

GAATTCACATAACCCCAAGCTTCGACCGCCCCAGATCATAACCGAACCGAATCTTCCCTCGAGCCAGGTCCACGTCGCTAGCAACGGCACCCTCGACCTGACCCAGCCCATGTGGACCTTATATGTGACAGGACCCGCCTTGAATCCTCGGAACCCGAGACGAATCCTGTGAAGACATTCCCGACATCACCCCCGATGTCGGGCCCACTTCTGAAATTATATCCCCTTTTGCCAAAAAATGAATAAGGGCACGAGACTTTCTACAGAAATTTCGGCAGAGTCTCCCCTGTAAATTGAACATTTCCCAAAAATGCTTCAACAACACATCTAATATCACCGACCGG

>1042E(ACA)-H/M(TTG)-270

GAATTCACAAACAATGTAGCAGATAAGATGAAGTCAGTTCCCTTGGCATAAAAAACAACTTTTATCCAATACCATAAAGTATGTTACATCAAGGGTTAAAACTTAAAAGGAATTTTTACTGTAGTGTCCCATGACTGGTTGCTTGAATCACTGATAATAAACATGAAAAGTGAAAGTTACTAGGCAGTGAATGACATCTTGCAGCTAAATTCATGTTAGCCAATCTACAAATCCCAAATCAACCGG

>1080E(ACA)-H/M(TTG)-365

GAATTCACAATTTCTTTTTTAATGCAAGGAACTAGTAATTTGAGATTCCCCGGAGCATTTTCCATCACAACTTCTCTAACTTTATCATCATTATCCCTGCAAGGAATTGCAATAGCTCCAAGTAATTTCCTCTATTGCTTGACGTAGCACTTTCATCATGGCCACGAAAAGCAAGACCTTGTCAATAAAAACTTAGTGCACTTGATTGATGCATTCAAGCAAGTGCGATAAGCCTTACGATTGGTCAGAGTGTTTGCTCACTGCAGTTTCAATATGTGTGTTTTAATTCATCAAATTTGTAGCAGTTCTTCTCTAGCTTTGTTATGAACGCTCCCAACCGG

>1221E(ACC)-H/M(TCC)-210

GAATTCACCGAGCATTGAAATTGGAAAATCAATTTATCAGCTCTGTGATTTGGAAGGGTGACTAAAGAAATTTTTATGGGAAGCTATTTCCGAGCGAATGTGTTCTTCCCGTGTCAGAAATTTTTGTTTCTATATGAATTTTTTTTTCCTACTTGTATATATAGGGCCTCGACATCCATGGACCGG

>1232E(ACC)-H/M(TCC)-231

GAATTCACCAAGCATTGAAATTGAAAAATCAATTTTTATCAGCTCTGTGATTTGGAATGGTGACTAAAGAAATTTTCTGGGAAGCTCTTTCCGAATGTGTTCTTCCCGAATGTCAGAAATTTTTGTAACATGGATTTTTTTTTTCCTACTTGTATATATAGGGCCTCGATCCATATTTGCACAGGGCCTCCAAAATAAATGGACCGG

>1234E(ACC)-H/M(TCC)-239

GAATTCACCAAGTATTGAATGGAAAATCAATTTATCAGCTATGTGATTTGGAAGGGTGACTAAAGAAATTTTTCTGGGAAGCTTTCCGAATGGAAGTTCTTCCGTGTCATAAATTTTTGTTTCTATATGGATTTTTTTCTTCCTACTTGTATATATAGGGCATCGACATTCCTATTCCTGCATAGAGCCTCCAAAATCAATGGACCGG

>1438E(ACC)-H/M(TTC)-279

GAATTCACCATTTAGCTTAGAGGCCTAATTACATTCTTATCAGCTCACCACTTACATTTCAACAACCTCTCCCTCTTTAAGATTTGATTTTGTTTTGGTGGGGAAGCTGGGGATTGGATTTTTTGTGGCTGGAGTTGAGATGACAGAGTAGGGAGCTAGGGGAATTGTTTAAAAAGCTGGGGGTTGGATTTTTTGTGGCGGGAGTGGAGATAACAAAGTGGAGAGCTAGGGATTGAATGGGTAGAAGAATGAATCGAGCTGCAGAACAAAGGATTATTATTTGTTCTAGGGGTAAAGTTGTCAATTTAGAAAGTTATTAGGGGTACTATGGGAAAAAGGAAATATCATTTAAAAGAACCGG

>1679E(ACG)-H/M(TCT)-377

GAATTCACGCTTCTTCTGCAACAACCATGGCTTTGAGAGTTTCCTCCTCCTCTTCTTCTTCTTCGTCTTCTTCCTTCTTCTCCATCTCCCCAAACCACAAGCCCACTCACTCCCTTTGAAGTTCAGCACTTTCAGACCTCCTCAACCAACCCTCCTAAAATCTTCAAATGCATCCAAACCCCGCCTTCCAATTCGAACAATTAAAGCAGCCTCCCAAAAACCCTAGCCCTGCCTCCCCCTTCTCCTGCTCTGCCCTACCCTCTCTTCCCCTCAAACCACCGAGCTCGTCCCCTGCAAGCTCCAAACTTTGATCTGAGTTTCAAGCTTTATCTGAGCCGATCGATAGACCGG

>1680E(ACG)-H/M(TCT)-385

GAATTCACGGTGAAAGGATGGAGGATGTGGTGATCATCGAGAAGATCTTGAGATCCATGACTCCTAAGTATGACTATGTTGTATGCTCGATCGAGGAATCAAATGATTTGGATGGGATTTTATCCATAGATGAGCTTCAAAGCAGTCTTTTGGTGCATGAACAACGGATCAGCCGTCACACTGTGGATGAACCCCTTCAGATCACTCACGGACAACCAGGAGGAAGTGGTGGTTCTTTTCGAGGAAGAGGAAGAGGTAGGGGTAGGTTTGATAAATCTACCCTAGAGTGTTATAACTGTCATGAGTTGGGTCATTTTCAATGGGAATGTCCCAAAAGAGCAAGAGATACAAAAGACCGG

>1837E(ACG)-H/M(TGT)-278

GAATTCACGAACGGAAAGTTCACAAGGCCTGCAACAAATTCACTGCCTTACTTTCATCTTTCTTTCGTTGAATCAAAAGCTCAATAAGAGAAATCAACTTTGCATGAATTTGTGGAAGCAGTGACAACTTATATGTAACCGTGACCTTCCACTCTGAATACATTGATCCAGCCTTGAAAATTTTTGAAACAATGGAATTTTCTTCTGTGCCATATTTAACGACCACAAGAAGCAGATACTTGATGCTGTCATAAGCTTCGATGACAGCACACCGG

>1954E(ACT)-H/M(TTC)-119

GAATTCACTTATCTCGGGGTATGCAGAACTGGGTTTGGGTATGAGGATCTATGGCGCTATACTTCACAAATGGAGGAAGAGGGAGTTGAACCGG

>2025E(ACT)-H/M(TTC)-271

GAATTCACTACGTCTCTGCCGGCAGGGTTCATCCCCGAGAAGCCCAAGACCGAGAAACGTCCCGACCCGGCAGCGCCTTTACCCGAAAAACCCTGTTTCAAGACTCCGGTTCCGGCCAAGGCCAGAAGCAAGCGAACCCGAACAGGCCCGGGTCTGGTCACTGGGGTCACCTTCGAATTGACGGAGACATCCTCGAGTTCTAGTTCCTCGTCTTCTTCTTCGTCTCCGTCGAGCCCCTGGCTCATTTATCCCCACCACCCAGAACCGG

>2046E(ACT)-H/M(TTA)-328

GAATTCACTACTGCGTACCAATTCACTCGGTTAATTTAGCAAATGTAGGACCAAGGGGCCTATATAAATGAGAATGGAATTATTGGTAGACATCTTGATGAAGAAAAGTCGGCTTACCGTGCATCGACGTCAGCCGCAGGCTCCCGTCAGGATGAGCAACCTGCACATGCGGGAGTCGCTAGGGTGCCCTTTTTTGGTCGGACTGAATCTCTCCCCACCTTTTCTGCCCGCGGTCTCAATCCCCCGTACTTTGCGAAGACCGTGGTTCATCCTCTAACCGAGCAGGACTCATGATAACCGG

>2230E(ACT)-H/M(TTA)-367

GAATTCACTATCATGAGTCCTGCTCGGTTAGAGCAGCGTAAGCGGAGCCTATACTTTTCATGGCATCTGGGGCTTCTTCATATAATTCTAGCAACAGCCCACAATGCAAAACACTTCAGCTATGCCAATGAGGCAATATTGGATCAACAACCAGAATGCACTGAGGTCTGACATTGACTTAAGAAACTACCCTCATATCCATGCTCTATTGCATAATTTCTTCGGTACCTTTCGAAAATTGCAGCCCATGCCACAGATAGGATTGAGACTGCCAAGCCTATGCCCACTCTCTGAAGCTGAGAAGCTCCATGAGGGTACCGAGTGAATTGGTACGCAGTAACCGG

>2240E(ACT)-H/M(TTA)-399

GAATTCACTCATGAAAGCATGGAAAGCCAGGAGGAAGAAGAACTACATAGGAAGTGCTAGATTTTGCACCAGCCGCACTGGATGACTCTCTGCCAGAGGAAGAAGATCCTTTGCGAGCCACTTAAGAAGGAACTAGTTGCACTTCTGCAAGAGTTCAGATTGTTTTGCTTGGCATTATCATGAGATGCCAGGATTAGACTCAGGAACTTGGTAGAACACAAGCTGCCAATCAAAGAGGGATACCTACCAGTCAAGCAGGCCAGAAGGAGGATGTCCATGGACACAGAACTGAAAGTCAAAGAAGAAATAGAAAGGCTGCTCAAGGCAGGATTCATCAGGCCTGCCATCTATGCTGATTGGCTGCTAACATTAACCGG

>2242E(ACT)-H/M(TTA)-415

GAATTCACTTTATTGTGCAAGTAAATTACAATGTAAAACTAAGACTGAATGAAAGATTTGTATCAGAAAGGAATAAAAATACAAGCTTATCCTTGCAGACTAAAGAAAATGCACTGAAATGAGATCATATGCAAAGCATGACTTGGTCATGAGTGACACCTGATCCTTTTTATTAAACAGACAATAGTCTATATATATTATCAAATTATTTACAATAAATCAGCACTAGTATGATCCTATTCTTTTTTATAAAAAAAAAACAAGAAAGAACCAAAAAAAAAATAAAAATAAAAAATAAAATAAAAAGAGACTGAAGGTAGACAATTTGATCACCATGTAGGTTTCTTGAGTTTGCGATGAGAGTTATGGAGGAAGAACAGCCTTTAACCGG

>2258E(ACT)-H/M(TTA)-478

GAATTCACTATCATGAGTCCTGCTCGGTTACTCGAGATTCCCGACTATTCCATTTCCTGATGTTAGCAATGTATAGCGGTCAAATAGGACCATTTTCTTCTCGAGACCTTTTACTTTTTTTCATCATGTGGGAGTTAGAATTAATTCCAGTTTATTTACTTCTATCCATGTGGGGGGGAAAGAAACGTTTGTATTCAGCTACAAAATTTATTTTGTACACTGCAGGAAGTTCTGTTTTTTTAATAGGAGTTCTGGGTATTGGATTTATACGGCTCCAATGAACCGACATTAAATTTTGAAACATCAGCTAATCAATCGTATCCTGTAGCACTGGAAATAATATTCTATATTGGATTTCTTATTGCTTTTGCTGTCAAATCACCGATCATACCCTTACATACATGGTTACCAGACACTCATGGAGAGGCGCATTACAGTACTTGTATGCTTTTAACCGG

>2364E(AGC)-H/M(TCT)-237

GAATTCAGCAACTGAATTGTTGAGACTTTTTTAATTATTTATAGTTAGCTCAATGAATAAGGTATAGGGGGCAAACAGAGTGTTAGAAGAACTAAAAGAAGAGTTGCGTTCAGTACCTGAAGAGGGACCTGGAAGCTAACATTAGCGGTAGCGGTTATTATGTGGTGTCTCTGAAGAAGAAGAAGAAGCAGAGGTTGGACGGAGGAGACCGG

>2390E(AGC)-H/M(TCT)-295

GAATTCAGCCTCAAAATTTAGTAACCCTATTATACGTTCATTGAAATCTACTTAATCCACAGAGTGTTCAACCGATTGATTGCTCTCACTTGTTTAACTATGATGGCATAGAGACATTCACATTCCATGTTTAACCATACGTATATTTTCCCTCTTTTTGGGGCCCGGATAGCAGGGTGAATGCCCTTAAATTGAGAGATTTCATACAAGAAATTCTTGTGGAACAGGGAAACTAAGCAGGAGATAGGCCACACCCCTTTACTTAGACCGG

>2491E(AGC)-H/M(TCC)-126

GAATTCAGCCGGTTCGGCCAAGGAAGCGAGTGCCGATGTCCAGGTTTCCGTGTCCGCCGACGAACCGGTGCCGTATCAGAAACCGGTTCAGGAGGACCGG

>2618E(AGC)-H/M(TCC)-397

GAATTCAGCAAAAAATCGAGGTGTGAAAAACCAAAGTGTTTGTGGGTTGACATAAATGAATACCTTTTCCTCCGGAAATTCAAGACCGGTGACTTGTGGGTAAGCTGATGGACCAACTTATGAACGCCGCCCATCCCAGAACTTCGTATGTGATTTCCAGGAATCGAATGTCCACGAAGCCATTCCTCCCTTCTCAAAATTGCAATTGCGATTTCCTACTTGTCTGATCAACTTTGAAGAGAGGGGAGAAGAGAAGAGGGAGAAGAAAGGGGCTTGCTTGCAATGACTTTTAAAACTTCAACTGTATCTGTATGTTTGCAATGTAATTGGCCCGTCCATCCAAAGTGGTCCATGGTGATGACGTGGACCGG

>2632E(AGC)-H/M(TCC)-458

GAATTCAGCATTCTACTTAATTAAGGAAGTTCCCAAAACCCAATTGAATAAAAAGAACTCCCAATTAAAACCCAATTAAGTAAGGAAATAGAATACCCTTTTGAAAAGCCCACAAAAAACCCTCGTGATACAGCAGCAGTTTCCCTTTTTTTTCTTTTTCTTTTCTTAATTTAAAAACCTTGGTCCAAAAGACGTCATTTTGGCCAATTTTTTTTTTTTTTTTTTTTAAAGGGGCATCGCACTTTAAAAAGCCTGGTCCAAAATGAGGTCGTTTTAGCCAGGCTTTGTTAAAAAAATAGAGGCCCGCGAGCGCAACACCAGTAGCTTCCCTAGCCCAGTGGTTTGCGCATTTCATCGAACACATTGTGTGCATCGCTGGTTTGAAAGTACGAGGTTTGCAACTCCAGCGTCTGTAATGGATATTTCCGTGGACCGG

>2751E(AGG)-H/M(TGA)-270

GAATTCAGGACTCATACCATTGCAATCTAGCCAACAGAGGGGAAATTCACAATCAAACAGCAACTCACTCACTCTTACCTAAAAATACATTACCTTATTTAACAGTCACCCCGCTGTCTTTAGAAGAATAGACTGTCAAAAAGCAGGCATATCTGGAACTCGGCCCAGGACTATGAAGCCCGAAGGCAACGCGTATCTCTGAACATCATCATTGTGACTCCTGTTACCCAATTCTACATGTATCACCGG

>2760E(AGG)-H/M(TGA)-294

GAATTCAGGTCCAGCTAAGCCCTATCATATATTTCGTTATGCATTCGTGGCATGAAAATAAAGTTAAGAATCTCTGCAAAGAATCTTGTTCATTCTAAGAAATATCTCATACAATACCCGCGTGGTAACTAACCTAAACAGAAATTAAAACGTCAAAATAATATGCGAATAAAAAAAATGAAATTAAAATTCAGTTAAAAAAAGAAAACGAAAAAGGTCCATAAGATCCTGAAAATCAAACACTGCACCCCAAATAAAAATGGGGTCACCGG
